# Parallel Pathways and Alternative Macrocyclizations in the Biosynthesis of Largimycins by Cytochrome P450 Minus Mutants of *Streptomyces argillaceus*

**DOI:** 10.1021/acs.jnatprod.6c00240

**Published:** 2026-05-07

**Authors:** Adriana Becerril, Ignacio Pérez-Victoria, Jesús Martín, José A. Salas, Fernando Reyes, Carmen Méndez

**Affiliations:** † Departamento de Biología Funcional, 16763 Universidad de Oviedo, Oviedo 33006, Spain; ‡ Instituto Universitario de Oncología del Principado de Asturias (I.U.O.P.A), Universidad de Oviedo, Oviedo 33006, Spain; § Instituto de Investigación Sanitaria de Asturias (ISPA), Oviedo 33011, Spain; ∥ Department of Biotechnology and Environmental Protection, Estación Experimental del Zaidín, Consejo Superior de Investigaciones Científicas, Granada 18008, Spain; ⊥ Fundación MEDINA, 328560 Centro de Excelencia en Investigación de Medicamentos Innovadores en Andalucía, Armilla, Granada 18016, Spain

## Abstract

Largimycins are a recently discovered group of hybrid nonribosomal peptide-polyketide compounds belonging to the leinamycin family of natural products. They are produced in *Streptomyces argillaceus* by the *lrg* biosynthetic gene cluster, which includes three cytochrome P450 encoding genes. By characterizing the compounds accumulated by mutants lacking these three genes, we have determined their essential roles in largimycin biosynthesis. Specifically, LrgC2 is involved in converting an olefinic exomethylene group into an oxo (ketone) group at position C-9, while LrgC1 is responsible for forming an epoxide on the C-3 side chain. Also, it is proposed that LrgC3 acts at an early stage of the biosynthetic pathway, probably hydroxylating an alanine residue during peptide assembly. The chemical structure of the compounds produced by *lrgC1*- and *lrgC2*-minus mutants suggests that a hydrolytic dehalogenation step proceeds before the peptide–polyketide chain is off-loaded from the polyketide synthase LrgJ. Remarkably, some compounds generated by these mutants bear a C-3 side chain that is cyclized into a unique β-thiolactone ring, a structural feature not seen before in natural products. Furthermore, this work has revealed alternative ways of peptide–polyketide chain off-loading by macrocyclization, unveiling the unprecedented flexibility of the thioesterase domain of LrgJ for selecting different intramolecular nucleophiles.

## Graphical abstract

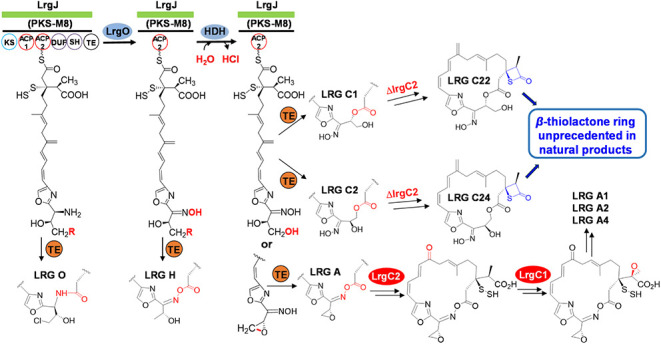

## Introduction

Largimycins (LRGs) (Figure 1A) are a recently discovered group of hybrid nonribosomal peptide (NRP)-polyketide (PK) natural products produced by *Streptomyces argillaceus* (LRG A1 and LRG A2) and *S. canus* (LRG A4).^1^ They belong to the scarcely represented leinamycin (LNM) family of antitumor natural products, named after the first compound discovered in this group (Figure 1B).^2^ Other members of this family include guangnanmycins (GNMs) and weishanmycins (WSMs) (Figure 1B).^3^ LRGs differ from other LNM-family members by several distinctive structural features (Figure 1): a 19-membered macrolactone ring cyclized through an unprecedented oxime ester bond instead of an 18-membered macrolactam ring; the presence of an oxazole rather than a thiazole ring; an epoxide group at the C-3 side chain; and either an *S*-conjugated *N*-acetyl-cysteine moiety or an additional epoxide at the C-17 side chain.

**Figure 1:**
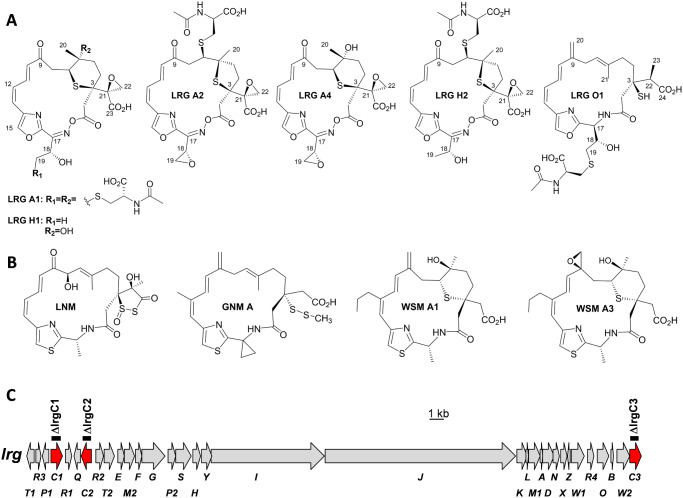
*lrg* biosynthetic gene cluster: (A) Chemical structures of Largimycins (LRGs): LRG A1, LRG A2, and LRG A4, encoded by the *lrg* biosynthetic gene cluster (BGC); LRG H1 and LRG H2, produced by the ΔlrgH mutant; and LRG O1, produced by the ΔlrgO mutant. (B) Chemical structures of some compounds from the leinamycin family of natural products: Leinamycin (LNM), guangnanmycin A (GNM A), weishanmycin A1 (WSM A1), and weishanmycin A3 (WSM A3). (C) Genetic organization of the *lrg* BGC: *lrg* genes encoding cytochrome P450s are in red. Other genes are light gray. Black bars indicate those genes deleted. Genes are shown to scale.

The *S. argillaceus* *lrg* biosynthetic gene cluster (BGC) has been identified and sequenced (MIBiG accession number BGC0001853) (Figure 1C).^1^ By generating mutants in selected *lrg* genes and characterizing their accumulated intermediates, we previously demonstrated that (i) the oxidoreductase LrgO is responsible for forming the oxime group, which participates in the oxime ester bond that closes the macrolactone ring; (ii) the bifunctional acyltransferase (AT)/decarboxylase (DC) LrgK, the acyl carrier protein (ACP) LrgL, and the hydroxymethylglutaryl-CoA synthase (HMGS) LrgM1 participate in the formation of the methylmalonyl-CoA-derived β*-*branch at C-3; (iii) the HMGS LrgM2 and the enoyl-CoA dehydratase (ECH1) LrgF are involved in the installation of the malonyl-CoA-derived olefinic exomethylene group at C-9; (iv) a cryptic chlorination step occurs during LRG biosynthesis, catalyzed by the halogenase LrgH; and (v) LRG biosynthesis proceeds through intermediates that are chlorinated at the threonyl residue and contain an olefinic exomethylene group at C-9, although these structural features are absent in the final native compounds (Figure 2).^1,4^ Although incorporation of the olefinic exomethylene group at C-9 is essential for the biosynthetic pathway to progress, the final LRG compounds lack it and contain a ketone (oxo) functionality instead (Figure 1A). Similarly, while LRGs lack a chlorine atom at C-19, the chlorination step is nevertheless crucial for the formation of the C-18/C-19 epoxide present in LRG A2 and LRG A4 (Figure 1A).^1,4^

**Figure 2:**
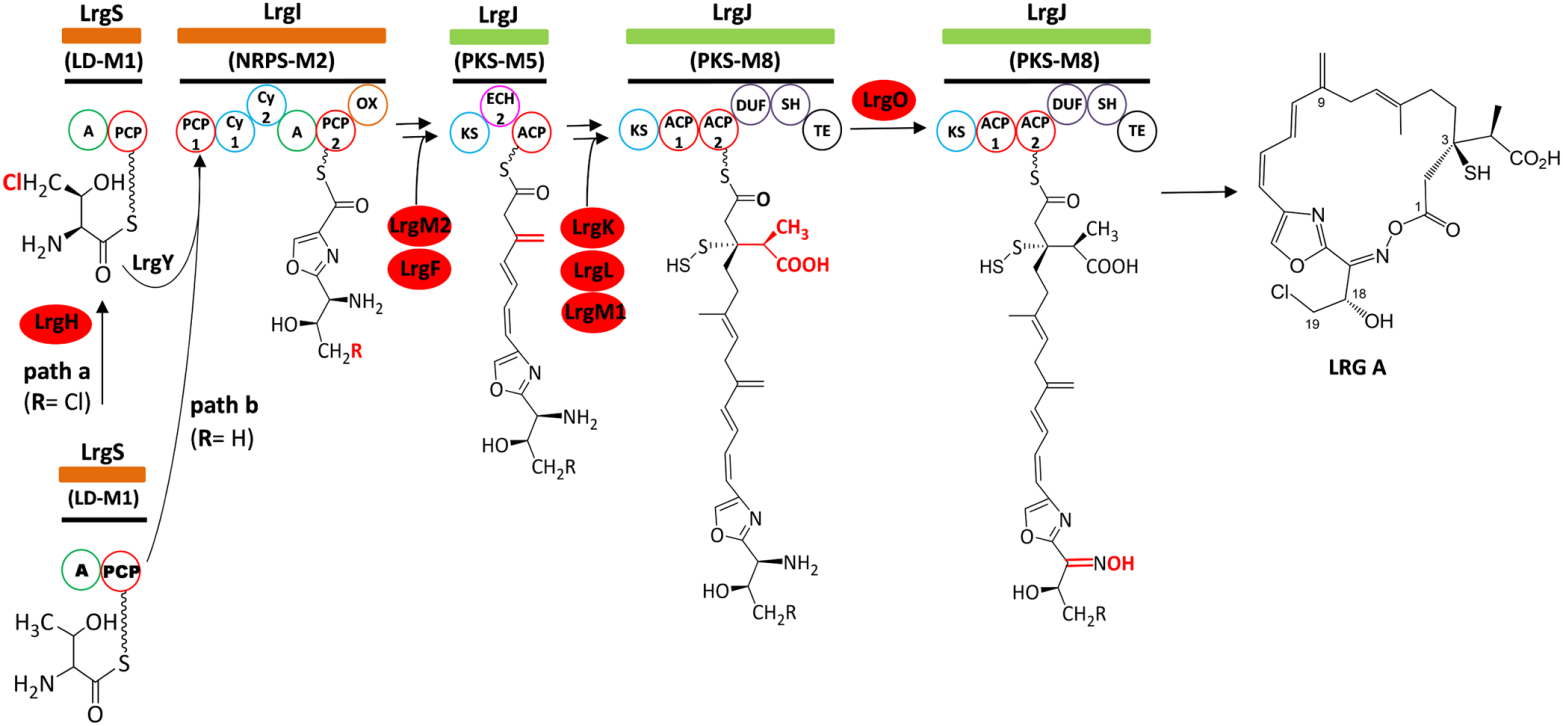
Proposed steps previously characterized in largimycins (LRGs) biosynthetic pathway. Red circles indicate those enzymes unambiguously identified to be involved in the transformations highlighted in red color.^1,4^

Despite the significant amount of information generated about the biosynthesis of compounds of this structural class, several steps in the LRG biosynthetic pathway still remain uncharacterized. These include, among others, the conversion of the C-9 olefinic exomethylene group into a C-9 oxo (ketone) group and the formation of the epoxide at the C-3 side chain. Candidate enzymes for these transformations are cytochromes P450 (P450/CYP), a family of heme-dependent monooxygenases frequently involved in natural product biosynthesis. They catalyze a wide range of oxidative reactions, including hydroxylations, oxygenations, dealkylations, dehydrogenations, biaryl ring couplings, rearrangements, epoxidations, sulfoxidation, and C–C bond cleavage.^5–7^ The *lrg* BGC harbors three P450 encoding genes—*lrgC1*, *lrgC2*, and *lrgC3*—that could catalyze the above-mentioned steps in LRG biosynthesis. Herein, we report that all three *lrg* P450-encoding genes are essential for LRGs production. Specifically, LrgC1 is responsible for introducing the epoxide at the C-3 side chain, while LrgC2 is involved in the conversion of the olefinic exomethylene group into an oxo (ketone) group at C-9. Although the function of LrgC3 could not be definitively assigned, we propose that it acts at a very early stage in LRG biosynthesis. Furthermore, we demonstrate that the dehalogenation step in LRG biosynthesis occurs prior to the off-loading of the NRP-PK chain from the polyketide synthase (PKS) LrgJ. Overall, we have shown that the biosynthesis of the various LRGs congeners proceeds through parallel pathways and involves alternative modes of cyclization of the nascent NRP-PK chain by the thioesterase (TE) domain of LrgJ. Moreover, generating mutants in the cytochrome P450 genes *lrgC1* and *lrgC2* has led to the production of several new LRG compounds, some of which featuring unprecedented structural motifs, that significantly broaden the structural diversity of this family of natural products.

## Results and Discussion

### Cytochrome P450s in the Largimycin Biosynthetic Gene Cluster

Based on the chemical structures of LRGs (Figure 1A) and those of compounds accumulated by different *lrg*-minus mutants,^1,4^ several steps between the hypothetical nascent macrocyclic oxime ester intermediate LRG A (Figure 2), synthesized by the hybrid nonribosomal peptide synthetase (NRPS)-PKS, and the final LRG products remain uncharacterized. These include, among others, the conversion of the olefinic exomethylene group at C-9 into an oxo (ketone) group and the introduction of an epoxide at the C-3 side chain. The *lrg* BGC encodes three cytochrome P450 enzymes—LrgC1, LrgC2, and LrgC3—that are plausibly involved in these transformations. LrgC1 belongs to the CYP170A1 family, which also includes *S. coelicolor* CYP170A1 (SCO5222) from the albaflavenone BGC and *S. avermitilis* PtlI (SAV2999) from the pentalenolactone gene cluster.^8,9^ LrgC2 is CYP107-like, a P450 family grouping with members of the CYP154 and CYP197 families, which includes *Saccharopolyspora erythraea* EryF from the erythromycin gene cluster.^10^ LrgC3 is CYP164-like, a broader P450 group encompassing multiple families, including, among others, *S. longisporoflavus* StaP from the staurosporine BGC.^11,12^

The presence of three P450 encoding genes (*lrgC1*, *lrgC2*, and *lrgC3*) within the *lrg* BGC (Figure 1C) is particularly intriguing, as the biosynthesis of LRGs would apparently require only two. In previous work, we demonstrated that *lrgC1* and *lrgC3* are essential for LRG biosynthesis, since deletion of either gene in *S. argillaceus* ΔlrgC1-R2 and *S. argillaceus* ΔlrgC3-R2 mutants abolished native LRGs production.^1^ However, characterization of the compounds accumulated by these mutants remained pending. To determine whether *lrgC2* is also required for LRG biosynthesis, this gene was deleted and replaced by an apramycin resistance cassette using plasmid pHZ-cit26. The genotype of the resultant mutant strain, *S. argillaceus* ΔlrgC2, was confirmed by PCR with primers Cit26 c up/Cit26 c rp (Table S1), which amplified a 1.85 kb DNA fragment from the mutant and a 1.6 kb from the WT strain, confirming the replacement of *lrgC2* by the apramycin resistance cassette (Figure S1). Because the *lrg* BGC is silent under standard conditions, the specific pathway activator *lrgR2* was overexpressed in *S. argillaceus* ΔlrgC2 using plasmid pEM4T-R2,^1^ generating strain *S. argillaceus* ΔlrgC2-R2. This strain, along with the *S. argillaceus* WT-R2 control, was cultivated to assess LRGs production. UPLC analysis of culture extracts revealed that native LRGs production was completely abolished (Figure 3B), confirming that *lrgC2* is also essential for LRG biosynthesis. Restoration of LRG production in this mutant strain was achieved by expressing a wild-type (WT) copy of *lrgC2 in trans* using plasmid pSETEH-cit26, generating *S. argillaceus* ΔlrgC2-R2-C2 (Figure S2).

**Figure 3:**
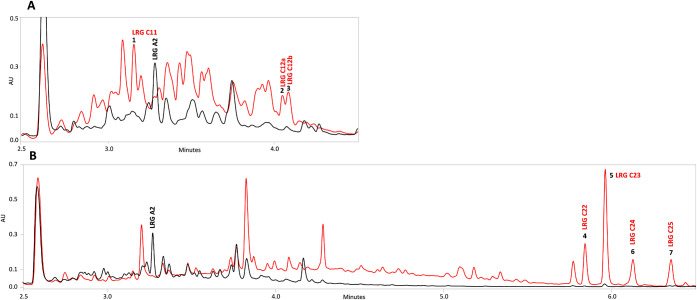
Production of largimycins (LRGs) by P450 minus mutants: (A) *S. argillaceus* ΔlrgC1-R2; (B) *S. argillaceus* ΔlrgC2-R2. UPLC chromatograms of culture extracts from *S. argillaceus* WT-R2 (black line) and mutant strains (red line) obtained at 330 nm (A) and 300 nm (B). Peaks with numbers correspond to compounds produced by mutants that have been purified and chemically characterized (names labeled in red). The peak corresponding to LRG A2 is labeled in black.

### LrgC1 Is Involved in the Incorporation of an Epoxide at the C-3 Side Chain, Whereas LrgC2 Drives the Conversion of the Olefinic Exomethylene into an Oxo (Ketone) Group at C-9

To investigate the specific roles of the different P450s in LRG biosynthesis, cultures of the P450 minus mutants *S. argillaceus* ΔlrgC1-R2, *S. argillaceus* ΔlrgC2-R2, and *S. argillaceus* ΔlrgC3-R2 were analyzed to assess the accumulated compounds. Each mutant was cultivated independently in SM30a medium, alongside *S. argillaceus* WT-R2 as a control, and cultures were extracted with ethyl acetate containing formic acid for metabolite profiling. UPLC analysis of extracts from *S. argillaceus* ΔlrgC1-R2 revealed the accumulation of several compounds (designated as LRG C1-compounds) (Figure 3A) eluting between 3 and 5 min, and with absorption maxima between 316 and 341 nm (see Supporting Information). Some of these compounds (peaks **1** to **3**, Figure 3A) were purified for structural characterization by HRMS and NMR (see Supporting Information). These analyses showed that peak **1** corresponds to a new compound, named LRG C11 (Figure 4A), with the molecular formula C_23_H_28_N_2_O_9_S. LRG C11 derives from a product of hydrolytic chain release and lacks the epoxide at the C-3 side chain but contains a keto group at C-9, excluding a role for the cytochrome P450 LrgC1 in converting the olefinic exomethylene group at C-9 into an oxo (ketone) group. Additionally, LRG C11 lacks both the *S*-conjugated *N*-acetyl-cysteine moiety and the epoxide at the C-17 side chain characteristic of certain LRG A compounds. Notably, it possesses a hydroxy group at C-19, a structural feature not previously reported in known LRGs.^1,4^ Interestingly, LRG C11 exhibits an *E* configuration for the C-12/C-13 double bond rather than the *Z* configuration observed in all macrocyclic LRGs.^1,4^ Such aberrant olefin stereochemistry—previously observed only in the truncated LRG intermediates LRG M1 and LRG M3 accumulated by *S. argillaceus* ΔlrgM2-R2^4^—is detrimental for macrocyclization and leads to hydrolytic chain off-loading. Spectroscopic analyses further revealed that peaks **2** and **3** correspond to two rotamers of a new compound with molecular formula C_23_H_28_N_2_O_8_S named as LRG C12. These rotamers, respectively, designated as LRG C12a (**2**) and LRG C12b (**3**) (Figure 4A), interconvert very slowly on the NMR and chromatographic time scales, being the LRG C12b (**3**) rotamer the more stable one (see Supporting Information). Similar to LRG C11 (**1**), LRGs C12 (**2** and **3**) lack the epoxide at the C-3 side chain, contain a keto group at C-9, and feature a hydroxy group at C-19. Interestingly, although LRGs C12 (**2** and **3**) retain the oxime functionality, the macrolactone ring is closed not through this group—as in LRG A2 (Figure 4C)—but via an ester bond involving the aliphatic hydroxy group at C-18. The chemical structure of LRGs C12 thus confirms that cytochrome P450 LrgC1 does not participate in the conversion of the olefinic exomethylene group at C-9 into an oxo (ketone) group, but it is rather involved in the incorporation of the epoxide at the C-3 side chain of native LRGs.^1^ In LNM biosynthesis, cytochrome LrgZ functions as a monofunctional enzyme that only hydroxylates C-4′ (the α-position in relation to the carbonyl group at the C-3 side chain).^13^ This contrasts sharply with the unexpected noncanonical role of LrgC1, which is involved in the epoxidation of the two alkylic carbons within the same C-3 side chain. Since P450-catalyzed epoxidation reactions in natural product biosynthesis are known to occur only at olefinic double bonds,^5^ our finding implies that LrgC1 would not be a monofunctional enzyme or may act cooperatively with additional oxidative enzymes. Future work will be required to elucidate the mechanism underlying this striking epoxidation of saturated alkylic carbons.

**Figure 4:**
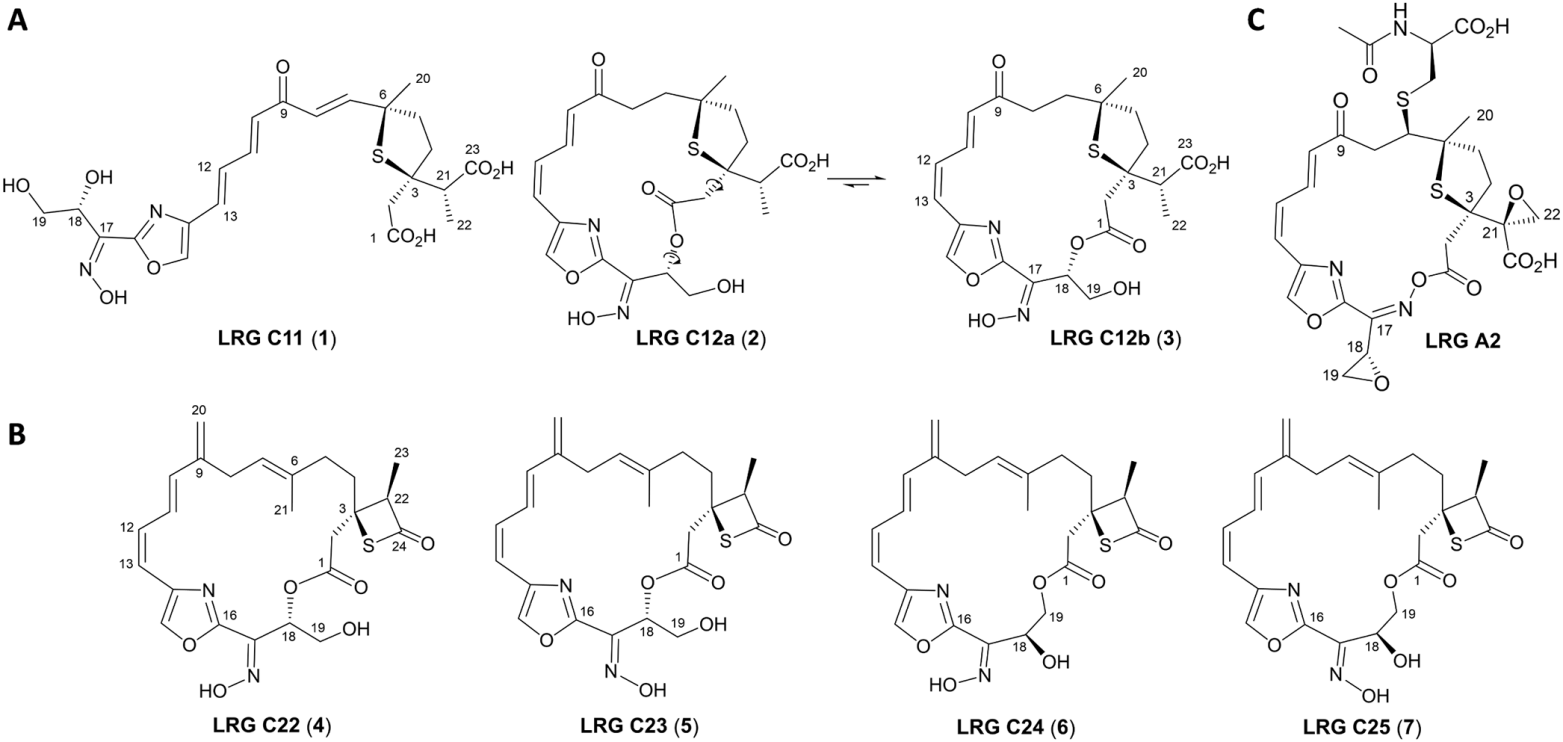
Chemical structures of largimycin (LRG) compounds produced by *S. argillaceus* ΔlrgC1-R2 (A) and *S. argillaceus* ΔlrgC2-R2 (B). Native LRG A2 produced by *S. argillaceus* WT strain is shown to facilitate structural comparisons (C).

Analyses of *S. argillaceus* ΔlrgC2-R2 chromatograms revealed that this mutant accumulated five major metabolites (designated as LRG C2-compounds) (Figure 3B), with retention times higher than 5 min, and absorption maxima around 297–299 nm (see Supporting Information). Four of these new compounds (**4**–**7**) were purified and structurally characterized (see Supporting Information). They share the same molecular formula, C_24_H_28_N_2_O_6_S, and were named LRG C22 (**4**), LRG C23 (**5**), LRG C24 (**6**) and LRG C25 (**7**) (Figure 4B). All LRG C2-compounds retain the olefinic β-exomethylene group at C-9 and lack the epoxide at the C-3 side chain. Remarkably, the C-3 side chain is cyclized into an unusual β-thiolactone ring (Figure 4B), a structural feature unprecedented among naturally occurring compounds.^14^ These LRG C2-compounds also lack the C-18/C-19 epoxide but instead display a hydroxy group at C-19. Again, although all these compounds contain the oxime functionality, macrolactone rings occur through aliphatic hydroxy groups either at C-18—LRG C22 (**4**) and LRG C23 (**5**)—or C-19—LRG C24 (**6**) and LRG C25 (**7**) (Figure 4B). Both *Z* and *E* configurations of the oxime hydroxy group are observed, giving rise to geometric isomer pairs around the oxime double bond—LRG C22 (**4**) vs LRG C23 (**5**) and LRG C24 (**6**) vs LRG C25 (**7**) (Figure 4B).

Metabolite profiling of the organic extracts from *S. argillaceus* ΔlrgC3-R2 cultures revealed neither the production of known LRGs nor the accumulation of any new related compounds (data not shown). Because we had previously determined that early intermediates in LRG biosynthesis are halogenated,^4^ we analyzed *S. argillaceus* ΔlrgC3-R2 cultures mining for the presence of halogenated intermediates bearing a chlorine atom in comparison with the WT cultures. However, this analysis did not detect any accumulated halogenated intermediates in the ΔlrgC3-R2 mutant (data not shown).

Taken together, these results confirm that all three Lrg P450 enzymes are essential for LRG biosynthesis. Specifically, LrgC2 is involved in the conversion of the C-9 olefinic exomethylene group into an oxo (ketone) functionality, while LrgC1 participates in the formation of the epoxide at the C-3 side chain. The role of LrgC3 remains unresolved, as the *lrgC3*-minus mutant did not accumulate any detectable LRG intermediates. This observation suggests that LrgC3 may participate in a yet unknown very early and possibly cryptic step of the LRG biosynthetic pathway. Interestingly, every *lnm*-type BGC identified to date contains at least one P450-encoding gene,^3^ and in some cases—such as in GNM biosynthesis—its precise function or putative role remains unclear.

### Reanalysis of the Biosynthetic Pathway of Largimycins

Biosynthesis of LRGs begins with the formation of a 4-Cl-l-threonyl-serine dipeptide, which is subsequently cyclized and oxidized by the NRPS LrgI to generate an oxazole ring (Figure 2, path a). Based on the chemical structures of LRGs, it was previously proposed that the adenylation (A) domain of LrgI NRPS would recognize l-serine, although its substrate specificity-conferring code more closely resembles those that recognize l-cysteine.^1^ Given that our results suggest LrgC3 P450 acts at a very early biosynthetic step, we speculate that the LrgI A domain may initially recognize a different amino acid. Once attached to the PCP2 domain of LrgI, such an amino acid could be hydroxylated by LrgC3 rendering serine. A plausible candidate for this precursor amino acid is alanine. Participation of cytochrome P450 enzymes in the β-hydroxylation of amino acids tethered to NRPS PCP domains has been reported in several biosynthetic pathways,^6^ including JBIR-35 and JNIR-34, novobiocin, telomycin, or skyllamycin.^15–18^

Additionally, based on the structures of compounds accumulated by *lrgC1*- and *lrgC2*-minus mutants (LRG C1- and LRG C2-compounds, respectively), which lack the chlorine at C-19 and instead display a hydroxyl group at this position (Figure 4), we propose the existence of a formal hydrolytic haloalkane dehalogenation step during LRG biosynthesis.^19,20^ This step would result in the substitution of chlorine with a hydroxy group at C-19. An alternative hydrolytic dehalogenation mechanism, involving intramolecular participation of the vicinal hydroxy group of the halohydrin moiety, would generate the epoxide at C-18/C-19 observed in LRG A2 and LRG A4 (Figure 1A). We propose that this dehalogenation occurs prior to off-loading of the nascent NRP-PK chain from the LrgJ PKS (Figure 5), rather than afterward as previously hypothesized,^1,4^ since LRG C2 macrolactones are cyclized through the hydroxy group at C-19.

**Figure 5:**
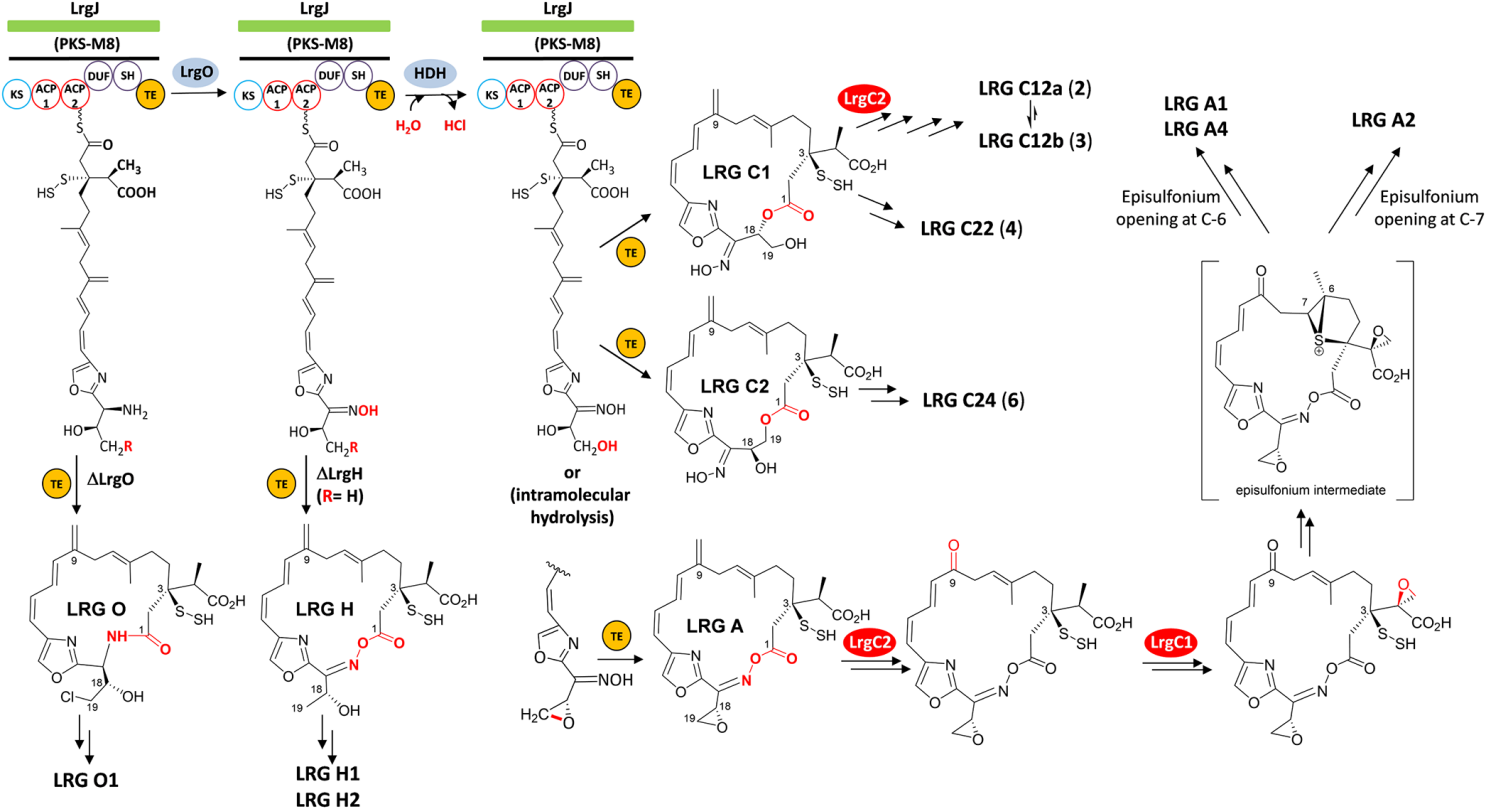
Revised biosynthetic pathway of largimycins (LRG): biosynthesis of different macrocyclic scaffolds and proposed roles of cytochromes P450 LrgC1 and LrgC2.

The putative nascent oxime macrolactone intermediate LRG A, off-loaded from LrgJ PKS, is predicted to contain an unstable hydropersulfide group at C-3, according to recent studies on the biosynthesis of members of the leinamycin family of natural products,^21,22^ and an olefinic exomethylene group at C-9 (Figure 5). This olefinic exomethylene group is subsequently converted into an oxo (ketone) group by cytochrome P450 LrgC2, while the epoxide at the C-3 side chain is installed by cytochrome P450 LrgC1. At the moment, the timing of these oxidation steps remains unclear, being possible to occur before or after macrocyclization. In any case, the production of final LRGs involves the formation of an episulfonium intermediate via a thiol-dependent or oxidative “activation” mechanism, which then nonenzymatically evolves toward LRG A1, LRG A2, or LRG A4 by nucleophilic (mycothiol or water) attack at C-6 or C-7 (Figure 5).^1^

Although cytochrome P450 enzymes have been reported to catalyze dealkylation reactions,^5^ examples of transformations converting olefinic exomethylene groups into oxo (ketone) groups are scarce. Interestingly, some WSMs (WSM A1 and WSM A2) also display an olefinic exomethylene group at the same position as in LRG C2 compounds, whereas WSM A3 (Figure 1B) contains a spiroepoxide instead.^3^ This observation suggests that the conversion of the C-9 olefinic exomethylene into the oxo (ketone) group in LRG biosynthesis could proceed via an initial epoxidation of the exocyclic double bond. This transformation likely occurs after macrolactone formation, since the olefinic exomethylene is retained in all truncated biosynthesis intermediates identified to date,^4^ as well as in the macrolactam LRG O1^1^ and in the LRG C2 compounds characterized in this work. Similar to LrgC1 and based on the canonical roles reported for cytochromes P450 in natural product biosynthesis,^5^ it is reasonable to postulate that LrgC2 functions in a concerted manner with other oxidative enzymes to ultimately convert the olefinic exomethylene into an oxo (ketone) group. Future investigations are likewise required to decipher the timing and the mechanism behind such a unique conversion.

The biosynthesis of LRG C2-compounds in the ΔlrgC2 mutant would proceed from LRG C1 or LRG C2 macrolactone scaffolds (Figure 5). Disproportionation of the intrinsically unstable hydropersulfide group at C-3 would generate a sulfhydryl group at this position,^21^ whose formal condensation with the carboxylic acid group at the C-3 side chain would yield the characteristic and unprecedented β-thiolactone ring of LRG C2-compounds (Figure 4B). It is tempting to speculate that an enzyme homologous to the one that must be involved in the formation of the 1,2-dithiolane-3-oxo ring in LNM biosynthesis could also catalyze β-thiolactone formation to some extent. However, this hypothesis must be regarded with caution, as the enzyme responsible for 1,2-dithiolane-3-oxo ring formation in the LNM family of natural products has yet to be identified. Conversely, LRG C1-compounds accumulated in the ΔlrgC1 mutant would arise after conversion of the olefinic exomethylene group at C-9 of LRG C1 macrolactone into an oxo (ketone) group by LrgC2, followed by further formation of the tetrahydrothiophene ring from nonenzymatic opening of the corresponding episulfonium intermediate.

### Structures of LRG C-Compounds Reveal Alternative Modes of Macrocyclization in LRG Biosynthesis

The chemical structures of native LRGs and the compounds produced by cytochrome P450 minus mutants reveal that the assembly line off-loading step in LRG biosynthesis can occur through alternative modes of macrocyclization. Typically, macrocyclization proceeds via the formation of an oxime ester bond, as observed in the native LRGs produced by the WT strain (Figure 5)^1^ and in the nonhalogenated LRG H-compounds produced by the halogenase-minus ΔlrgH mutant (Figure 5).^4^ Noticeably, the compounds produced by the ΔlrgC1 and ΔlrgC2 mutants also contain the oxime group but are cyclized in a different manner. In these cases, macrocyclization occurs through an ester bond that does not involve the oxime group, but rather an aliphatic hydroxy group located at either C-18—LRG C1-compounds, LRG C22 (**4**) and LRG C23 (**5**)—or C-19—LRG C24 (**6**) and LRG C25 (**7**)—, resulting in 19- and 20-membered macrolactone rings, respectively (Figures 4 and 5). Conversely, biosynthetic intermediates lacking the oxime group cyclize via amide bond formation, as observed in LRG O1 from the ΔlrgO mutant (Figure 5).^1^ These findings indicate that the LrgJ-TE domain exhibits remarkable flexibility, capable of forming different types of bonds and employing intramolecular nucleophilic groups located at different positions for chain release. Similar substrate tolerance has been reported for other TE domains.^23^ For instance, the PKS TE domain involved in conglobatin biosynthesis can utilize either intramolecular or external hydroxyl nucleophiles for esterification,^24^ while the TE domain involved in the biosynthesis of the lipopeptide haerogladin from *Burkholderia gladioli* can employ either the hydroxyl or amino group of l-threonine as external nucleophiles for chain release.^25^ Remarkably, the LrgJ-TE domain can generate 18-membered macrolactams by selecting an intramolecular amine for amide bond formation, or 19- and 20-membered macrolactone rings by selecting intramolecular hydroxy groups either aliphatic or from an oxime group—as nucleophiles. Thus, LrgJ-TE represents an unprecedented example of flexible chain release by macrocyclization based on alternative selection of distinct intramolecular nucleophiles. The intrinsic favorable conformational preorganization of the unreleased LRG chain likely facilitates this flexible TE catalyzed macrocyclization.^26^ Equilibrium rotamers around the C-16/C-17 and C-17/C-18 bonds position the alternative nucleophilic groups (amino, oxime hydroxyl, or aliphatic hydroxyls) at an appropriate distance and orientation to attack the C-1 carbonyl tethered to the ACP2 domain of the terminal PKS module of LrgJ.

In conclusion, we have elucidated the roles of the cytochromes P450 involved in the biosynthetic pathway of LRGs by generating mutants in their respective encoding genes within the *lrg* BGC and characterizing the compounds accumulated in each case. LrgC2 is required for converting the olefinic exomethylene group into an oxo (ketone) group at C-9, while LrgC1 is involved in the formation of the epoxide at the C-3 side chain. Notably, this work has also led to the discovery of new LRG compounds that expand the structural diversity of this family of natural products. Particularly important is the identification of the first β-thiolactone-containing natural products ever reported. Finally, our findings have unveiled alternative modes of macrocyclization in LRG biosynthesis, revealing that LrgJ-TE possesses remarkable substrate flexibility through an unprecedented capacity to select distinct intramolecular nucleophiles. This catalytic versatility not only expands our understanding of LRG biosynthesis but indeed could be harnessed to explore the generation of chemical diversity in the LNM family of natural products.

## Experimental Section

### General Experimental Procedures

*S. argillaceus* ATCC 12956 was used as the source of DNA. *S. argillaceus* WT-R2 was used as the control for LRGs production.^1^ *S. argillaceus* ΔlrgC1-R2 and ΔlrgC3-R2 mutants were used to analyze their metabolite profiles and to purify their accumulated compounds.^1^ pEM4T-R2 was used to overexpress the activator *lrgR2* gene into mutant strains.^1^ *S. argillaceus* strains were cultivated in SM30a as previously reported.^1^ *Escherichia coli* DH10B and *E. coli* ET12567/pUB307 were used as the cloning host and for conjugation experiments between *E. coli* and *S. argillaceus*, respectively.^27^ When antibiotic selection was required, the corresponding antibiotics were added to the culture media at the following final concentrations: ampicillin (100 μg/mL), hygromycin (200 μg/mL), nalidixic acid (25 μg/mL), apramycin (100 μg/mL for *E. coli* and 25 μg/mL for *Streptomyces*), and thiostrepton (5 μg/mL and 50 μg/mL in liquid and solid media, respectively). Plasmids pUO9090, pHZ1358, and pSETEcH were used as vectors along this work.^28–30^ DNA isolation and manipulation, intergeneric conjugations, and transformations were carried out according to standard procedures for *Streptomyces* and *E. coli*.^27,31^

### Generation and Complementation of *S. argillaceus* ΔlrgC2 Mutant Strain

This mutant was generated by deleting most of the *lrgC2* coding region and its replacement by an apramycin resistance cassette, which was inserted in the same direction of transcription of *lrgC2* to avoid polar effects on downstream genes (Figure S1). To this aim, plasmid pHZ-cit26 was constructed as follows: first, a 1.97 kb DNA fragment containing the 5′-end of *lrgT2*, *lrgR2* and the 5′-end of *lrgC2* was amplified using oligonucleotides Cit26I up/Cit26I rp (Table S1), digested with *Eco*RI and *Hin*dIII, and subcloned into the same sites of pUO9090, upstream of the apramycin resistance cassette, generating pUO-cit26I. Then, a 2.0 kb DNA fragment containing *lrgR1*, *lrgQ* and the 3′-end of *lrgC2* was amplified using oligonucleotides Cit26D 2 up/Cit26D 2 rp (Table S1), digested with EcoRV and XbaI, and subcloned into the same sites of pUO-cit26I, downstream of the apramycin cassette, generating pUO-cit26. Finally, the whole fragment (5.5 kb) was rescued as a SpeI fragment and subcloned into the XbaI site of pHZ1358. The resultant plasmid pHZ-cit26 was introduced into *S. argillaceus* by conjugation. Apramycin-resistant, thiostrepton-sensitive colonies were selected as mutants. The genotype of mutant strains was confirmed by PCR and by sequencing the corresponding amplicons, using oligoprimers Cit26 up/Cit26 rp (Table S1). To complement *S. argillaceus* ΔlrgC2 mutant, a plasmid (pSETEH-cit26) was constructed as follows: a 1.39 kb DNA fragment containing *lrgC2* was PCR amplified using oligonucleotides ermECit26 up/ermECit26 rp (Table S1), digested with NheI and SpeI, and subcloned in the right direction into the XbaI site of pSETEcH, downstream of the erythromycin resistance promoter.

### Chromatographic Analysis and Purification of Compounds

Extraction of compounds and UPLC analyses were performed as previously described.^1,4^ For purification purposes, *S. argillaceus* ΔlrgC1-R2 and *S. argillaceus* ΔlrgC2-R2 strains were cultivated by a two-step culture method.^32^ In the production step, six 2 L Erlenmeyer flasks, each containing SM30a medium (400 mL), were incubated for 4 and 5 days for *S. argillaceus* ΔlrgC2-R2 and *S. argillaceus* ΔlrgC1-R2, respectively. To purify LRG C11 (**1**), LRG C12a (**2**) and LRG C12b (**3**), cultures of *S. argillaceus* ΔlrgC1-R2 were extracted with ethyl acetate plus 1% formic acid. Extracts were preliminarily fractionated into three major fractions by applying a linear gradient of acetonitrile, by preparative HPLC using a SunFire C18 column (10 mm, 10 × 150 mm, Waters). These fractions were analyzed by UPLC, evaporated in vacuo, and dissolved in a small volume of DMSO:methanol (1:1). To purify LRG C22 (**4**) to LRG C25 (**7**) compounds, cultures were centrifuged, and cells were extracted with ethyl acetate plus formic acid. The organic extracts were dried down under vacuum, and residues were dissolved in a small volume of DMSO:methanol (1:1). Extracts enriched in the target compounds were purified by preparative HPLC. They were chromatographed with mixtures of acetonitrile or methanol, and 0.05% TFA in water, in isocratic conditions optimized for each compound, at 5 mL/min. For LRG C11 (**1**), LRG C12a (**2**), and LRG C12b (**3**), mixtures containing 20–40% of organic solvents were used, while for LRG C22 (**4**) to LRG C25 (**7**), mixtures containing 65–80% organic solvent were needed. The purification procedure afforded LRG C22 (**4**) (0.9 mg), LRG C23 (**5**) (3.4 mg), LRG C24 (**6**) (0.7 mg), and LRG C25 (**7**) (0.4 mg) from *S. argillaceus* ΔlrgC2-R2; and LRG C11 (**1**) (0.5 mg), LRG C12a (**2**) (1.4 mg), and LRG C12b (**3**) (1.1 mg) from *S. argillaceus* ΔlrgC1-R2, all as amorphous yellowish solids.

### Spectroscopic Analysis and Structural Elucidation of Compounds

Structural elucidation of each compound was carried out by ESI-TOF mass spectrometry and NMR spectroscopy, further assisted by comparisons with the reported spectroscopic data of known LRGs.^1,4^ HRMS spectra were collected by LC-MS analyses using an Agilent 1200RR HPLC equipped with an SB-C8 column (2.1 × 30 mm, Zorbax) and coupled to a Bruker maXis spectrometer. Chromatographic and ionization conditions were identical to those previously described.^33,34^ UV/vis (DAD) spectra were also collected in the same chromatographic analyses. NMR spectra were recorded in DMSO-*d*_6_ or CD_3_OD at 24 °C on a Bruker AVANCE III-500 MHz (500 and 125 MHz for ^1^H and ^13^C NMR, respectively) equipped with a 1.7 mm TCI MicroCryoProbe, using the residual solvent signal as an internal reference (δ_H_ 2.50 and δ_C_ 39.5 for DMSO-*d*_6_; δ_H_ 3.31 and δ_C_ 49.0 for CD_3_OD). The full connectivity of the compounds was established based on the key correlations observed in the COSY and HMBC spectra in combination with the determined molecular formulas (based on the experimental accurate mass of each compound). Stereochemistry was determined based on that reported for known LRGs and their shared biosynthetic origin.^1,4^ Molecular models of conformers LRG C12a (**2**) and LRG C12b (**3**) were generated with Chem3D Pro 12.0 starting from the modeled 3D structure of known LRGs,^1,4^ which were based on the reported X-ray structure of LNM E2.^35^ Energy minimization by molecular mechanics employed the MM2 force field using as gradient convergence criteria an RMS value of 0.001. Molecular modeling images (Figure S45) were generated with PyMOL.^36^ A detailed description of the structure elucidation of the new LRGs is included in the Supporting Information.

## Supplementary Material



## Data Availability

The NMR data for largimycin compounds described in this work have been deposited in the Natural Products Magnetic Resonance Database (www.np-mrd.org) and can be found at NP0352047 (**1**), NP0352048 (**3**), NP0352049 (**4**), NP0352050 (**5**), NP0352051 (**6**), and NP0352052 (**7**).

## Notes

Structure elucidation of largimycins; Generation and genetic confirmation of *S. argillaceus* ΔlrgC2 mutant; UPLC analyses of extracts of complemented *S. argillaceus* ΔlrgC2 mutant; Spectroscopic data of LRG C11 (**1**), LRG C12a (**2**), LRG C12b (**3**), and LRG C22–C25 (**4**-**7**); Key COSY and HMBC correlations; Equilibrium conformers LRG C12a (**2**) and LRG C12b (**3**); Oligonucleotides used for PCR amplifications; ^1^H NMR and ^13^C NMR data tables for LRG C11 (**1**), LRG C12a (**2**), LRG C12b (**3**), and LRG C22–C25 (**4**–**7**) (PDF)

## #

A.B.: Bioinsectis SL, Polígono Industrial Mocholi, Pl. Cein, n° 5, Office A14, 31110, Noáin, Navarra, Spain
