# Supplementary material for: Parallel Pathways and Alternative Macrocyclizations in the Biosynthesis of Largimycins by Cytochrome P450 Minus Mutants of Streptomyces argillaceus
Source: J Nat Prod. 2026 May 7;89(5):1579–87. doi: 10.1021/acs.jnatprod.6c00240 (PMC13200230; doi:10.1021/acs.jnatprod.6c00240)
Supplement: Supplementary file 1 [file np6c00240_si_001.pdf]

Supporting Information

Parallel pathways and alternative macrocyclizations in the biosynthesis of largimycins by cytochrome P450-minus mutants of *Streptomyces argillaceus*

Adriana Becerril,[1,2,3‡] Ignacio Pérez-Victoria[4,5‡] Jesús Martín,[5] José A. Salas,[1,2,3] Fernando Reyes[5] and Carmen Méndez*[1,2,3]

[1]Departamento de Biología Funcional, Universidad de Oviedo, 33006 Oviedo, Spain

[2]Instituto Universitario de Oncología del Principado de Asturias (I.U.O.P.A), Universidad de Oviedo, 33006 Oviedo, Spain

[3]Instituto de Investigación Sanitaria de Asturias (ISPA), 33011 Oviedo, Spain

[4]Department of Biotechnology and Environmental Protection, Estación Experimental del Zaidín, Consejo Superior de Investigaciones Científicas, 18008 Granada, Spain.

[5]Fundación MEDINA, Centro de Excelencia en Investigación de Medicamentos Innovadores en Andalucía, Armilla, 18016 Granada, Spain.

*Email: cmendezf@uniovi.es

‡ *A.A. and I.P.-V contributed equally to this work*

KEYWORDS: largimycin, leinamycin, cytochrome P450, olefinic *β*-exomethylene, epoxide, thioesterase, cryptic halogenation, *β*-thiolactone, *Streptomyces argillaceus*

Table of contents

Structure elucidation of largimycins…………………………………………………………. 4
Figure S1. Generation and genetic confirmation of *S. argillaceus* ΔlrgC2 mutant……….. 9
Figure S2. UPLC analyses of extracts of complemented *S. argillaceus* ΔlrgC2 mutant… 9
Figure S3. UV-DAD and HRMS spectra of LRG C11 (**1**)…………………………………… 10
Figure S4. ^{1}H NMR spectrum of LRG C11 (**1**) (500 MHz, 24 ºC)………………………... 11
Figure S5. COSY spectrum of LRG C11 (**1**)……………………………………………….. 12
Figure S6. Edited HSQC spectrum of LRG C11 (**1**)………………………………………… 13
Figure S7. HMBC spectrum of LRG C11 (**1**)……………………………………………….. 14
Figure S8. UV-DAD and HRMS spectra of LRG C12a (**2**)……………………………..... 15
Figure S9. ^{1}H NMR spectrum of LRG C12a (**2**) (500 MHz, 24 ºC)……………………….. 16
Figure S10. COSY spectrum of LRG C12a (**2**)……………………………………………. 17
Figure S11. ROESY spectrum of LRG C12a (**2**)…………………………………………… 18
Figure S12. Edited HSQC spectrum of LRG C12a (**2**)……………………………………. 19
Figure S13. HMBC spectrum of LRG C12a (2)……………………………………………….. 20
Figure S14. UV-DAD and HRMS spectra of LRG C12b (**3**)…………………………………. 21
Figure S15. ^{1}H NMR spectrum of LRG C12b (**3**) (500 MHz, 24 ºC)……………………… 22
Figure S16. COSY spectrum of LRG C12b (**3**)……………………………………………... 23
Figure S17. ROESY spectrum of LRG C12b (**3**)……………………………………………. 24
Figure S18. Edited HSQC spectrum of LRG C12b (**3**)…………………………………….. 25
Figure S19. HMBC spectrum of LRG C12b (**3**)……………………………………………….. 26
Figure S20. UV-DAD and HRMS spectra of LRG C22 (**4**)…………………………………… 27
Figure S21. ^{1}H NMR spectrum of LRG C22 (**4**) (500 MHz, 24 ºC)……………………….. 28
Figure S22. COSY spectrum of LRG C22 (**4**)………………………………………………… 29
Figure S23. NOESY spectrum of LRG C22 (**4**)……………………………………………… 30
Figure S24. Edited HSQC spectrum of LRG C22 (**4**)……………………………………….. 31
Figure S25. HMBC spectrum of LRG C22 (**4**)………………………………………………… 32
Figure S26. UV-DAD and HRMS spectra of LRG C23 (**5**)………………………………….. 33
Figure S27. ^{1}H NMR spectrum of LRG C23 (**5**) (500 MHz, 24 ºC)……………………….. 34
Figure S28. COSY spectrum of LRG C23 (**5**)………………………………………………… 35
Figure S29. NOESY spectrum of LRG C23 (**5**)……………………………………………… 36
Figure S30. Edited HSQC spectrum of LRG C23 (**5**)……………………………………….. 37
Figure S31. HMBC spectrum of LRG C23 (**5**)………………………………………………… 38
Figure S32. UV-DAD and HRMS spectra of LRG C24 (**6**)………………………………….. 39
Figure S33. ^{1}H NMR spectrum of LRG C24 (**6**) (500 MHz, 24 ºC)……………………….. 40

Figure S34. COSY spectrum of LRG C24 (**6**).. 41
Figure S35. NOESY spectrum of LRG C24 (**6**).. 42
Figure S36. Edited HSQC spectra of LRG C24 (**6**)... 43
Figure S37. HMBC spectrum of LRG C24 (**6**).. 44
Figure S38. UV-DAD and HRMS spectra of LRG C25 (**7**)... 45
Figure S39. ^{1}H NMR spectrum of LRG C25 (**7**) (500 MHz, 24 ºC).............................. 46
Figure S40. COSY spectrum of LRG C25 (**7**).. 47
Figure S41. NOESY spectrum of LRG C25 (**7**).. 48
Figure S42. Edited HSQC spectrum of LRG C25 (**7**)... 49
Figure S43. HMBC spectrum of LRG C25 (**7**).. 50
Figure S44. Key COSY correlations and 1H to 13C HMBC correlations determining the connectivity of LRG C11 (**1**), C12 (both conformers **2** and **3**), C22 (**4**), C23 (**5**), C24 (**6**) and C25 (**7**)... 51
Figure S45. Equilibrium conformers LRG C12a and LRG C12b................................. 51
Table S1. Oligonucleotides used for PCR amplification.. 52
Table S2. ^{1}H NMR (500 MHz) and ^{13}C NMR (125 MHz) data of LRG C11 (**1**) in DMSO-d_6... 53
Table S3. ^{1}H NMR (500 MHz) and ^{13}C NMR (125 MHz) data of LRG C12a (**2**) in DMSO-d_6... 54
Table S4. ^{1}H NMR (500 MHz) and ^{13}C NMR (125 MHz) data of LRG C12b (**3**) in DMSO-d_6... 55
Table S5. ^{1}H NMR (500 MHz) and ^{13}C NMR (125 MHz) data of LRG C22 (**4**) in CD_3OD... 56
Table S6. ^{1}H NMR (500 MHz) and ^{13}C NMR (125 MHz) data of LRG C23 (**5**) in CD_3OD... 57
Table S7. ^{1}H NMR (500 MHz) and ^{13}C NMR (125 MHz) data of LRG C24 (**6**) in DMSO-d_6... 58
Table S8. ^{1}H NMR (500 MHz) and ^{13}C NMR (125 MHz) data of LRG C25 (**7**) in DMSO-d_6... 59
References.. 60

Structure elucidation of largimycins

Largimycin C11 (**1**) was assigned a molecular formula $C_{23}H_{28}N_2O_9S$ based on the observed ion $[M+H]^+$ at *m/z* 509.1594 (calcd. for $C_{23}H_{29}N_2O_9S^+$, 509.1588, Δ 1.2 ppm) alongside its corresponding isotopic pattern, indicating 11 degrees of unsaturation. The structure of **1** was determined by detailed 1D (^{1}H) and 2D NMR (COSY, NOESY, HSQC and HMBC) spectroscopic analyses further assisted by comparison of its NMR data (Table S2) with those of already reported LRGs.[1, 2] Interpretation of the HSQC and HMBC spectra revealed the presence of eight quaternary carbons (including two carboxylic acid carbonyls in the range δ_C 172-177, a doubly α,β unsaturated ketone at δ_C 189.1, three sp^2 carbons at δ_C 138.7, 145.2 and 154.8, and two sp^3 carbons at δ_C 57.4 and 62.8), seven sp^2 methine carbons (resonating at δ_C 124.2, 129.2, 129.2, 129.8, 139.4, 142.8, and 152.4), two sp^3 methines (one oxygenated methine and one aliphatic methine), one olefinic methylene, four aliphatic methylenes (one of them oxygenated) and two aliphatic methyl groups. Analysis of COSY correlations identified different spin systems which could be connected using the key long-range correlations observed in the HMBC spectrum (Figure S44). The spin system comprising H-10 to H13, contains four olefinic protons corresponding to two *E* double bonds, as indicated by the measured coupling constants ($J_{10\text{-}11}$ = 15.3 Hz, $J_{12\text{-}13}$ = 15.2 Hz). This spin system is conjugated on the H-13 end with the aromatic oxazole heterocycle characteristic of LRGs, as revealed by the key HMBC correlations between H-15 and C-13, C-14 and C-16. On the opposite H-10 end, this spin system is conjugated with a ketone group, as indicated by the key long-range correlations between H-11/C-9 and H-10/C-9. This ketone is also conjugated with the spin system comprised by the Δ^7 *E* double bond, as proved by the key HMBC correlations between both H-7 and H-8 with the ketone C-9 carbon. This double bond is substituted at the C-7 end with the same tetrahydrothiophene ring present in LRG A2,[1] LRG H2,[2] and LNM E5.[3] Such saturated heterocycle ring shows the same substituents as those found in LNM E5,[3] as revealed by the corresponding key HMBC correlations observed (Figure S44). In fact, the chemical structure related to positions C-1 to C-9 and C-20 to C-21 is identical in both LRG C11 and LNM 5 as additionally reflected by the similarity of the chemicals shift associated to those positions in both compounds. Thus, LRG C11 does not display an epoxide on the C-3 side chain as clearly evidenced by the presence of the aliphatic methyl doublet H-22. No epoxide signals were observed neither in the Thr-derived moiety (C-18/C-19), and interestingly C-19 turned out to be a hydroxylated methylene. Key long-range correlation between H-18 and H-19 with the sp^2 C-17 at δ_C 145.2 confirmed the presence of the oxime functionality characteristic of native LRGs.[1] However, LRG C11 is not a macrocycle since carboxylic acid at position C-1 is not esterified. The oxime double bond was assigned a *Z*

configuration based on the observed δ_C 154.8 for C16 and comparison with the empirical chemical shift prediction obtained for the two possible *E*/*Z* configurations of the oxime double bond, as it was described for the first LRGs discovered.[1] The absolute configuration of the chiral centers at C-3 and C-6, C-18 and C-21 was assigned to be the same as for all previously reported LRGs, based on their common biosynthetic origin.[1, 2]

Largimycin C12 was isolated as two independent conformers, LRG C12a (**2**) and LRG C12b (**3**), that interconvert so slowly that can be separated by HPLC. Taking each conformer as a pseudo independent compound, structure elucidation was carried out independently for each of them. LRG C12a (**2**) was assigned the molecular formula $C_{23}H_{28}N_2O_8S$ based on the observed ion $[M+H]^+$ at *m/z* 496.1639 (calcd. for $C_{23}H_{29}N_2O_8S^+$, 496.1639, Δ 0 ppm) alongside its corresponding isotopic pattern, indicating 11 degrees of unsaturation. The structure of **2** was established after detailed 1D (^1H) and 2D NMR (COSY, ROESY, HSQC and HMBC) spectroscopic analyses further assisted by comparison of its NMR data (Table S3) with those of LRG C11 and already reported LRGs.[1, 2] NMR features are very similar to those observed for LRG C11 with the important difference that LRGC12a has two less olefinic methines and gain two aliphatic methylenes, suggesting one of the double bonds present in LRG C11 is saturated in LRG C12a. Analysis of COSY correlations identified different spin systems which could be connected using the key long-range correlations observed in the HMBC spectrum (Figure S44). Connectivity is essentially identical to that determined for LRG C11 with some key differences: i) The C-12/C-13 double bond displays the usual *Z* configuration present in all macrocyclic LRGs, as revealed by the measured coupling constant (J_{H12H13} = 11.2 Hz); ii) The C-7/C-8 double bond is not present and those positions are saturated; iii) The compound is not an open chain but a macrocycle. Interestingly, the key HMBC correlation between H-18 and C-1 indicates that ring closure takes place as an ester bond involving the oxygen at position C-18, accounting for the deshielded $^{13}C/^{1}H$ chemical shifts observed for the oxygenated methine at that position compared to those of the non-esterified LRG C11. The oxime double bond was assigned a *Z* stereochemistry based the observed δ_C 154.2 for C16 and comparison with the empirical chemical shift prediction obtained for the two possible *E*/*Z* configurations of the oxime double bond, as previously indicated. Likewise, as already indicated for LRG C11, the absolute configuration of the chiral centers at C-3 and C-6, C-18 and C-21 was assigned to be the same as for all previously reported LRGs, based on their common biosynthetic origin.[1, 2] For the other conformer, LRG C12b, the same molecular formula was obviously found after observing the ion $[M+H]^+$ at *m/z* 496.1640 (calcd. for $C_{23}H_{29}N_2O_8S^+$, 496.1639, Δ 2 ppm). Its NMR data (Table S4) are logically very similar to those of LRG C12a and, obviously, an identical

pattern of COSY and HMBC correlations were observed for both conformers (Figure S44). The essentially identical multiplicity pattern and coupling constant values observed for both conformers suggest likewise almost identical conformations of the molecular backbone. Molecular modelling showed that two possible orientations of the macrocyclic ester bond account for these observations. It could be established that LRG C12a corresponds to the conformer with the C-1 carbonyl directed towards the inside of the macrocycle while C12b corresponds to the conformer where the C-1 carbonyl points out of the macrocycle (Figure S45). Conformer LRG C12b is ca. 0.5 kcal/mol more stable than conformer LRG C12a, accounting for the trend of slow interconversion observed by NMR after three days (Figure S45).

Largimycin C22 (**4**) was assigned a molecular formula $C_{24}H_{28}N_2O_6S$ based on the observed ion $[M+H]^+$ at *m/z* 473.1746 (calcd. for $C_{24}H_{29}N_2O_6S^+$, 473.1741, Δ 1.1 ppm) alongside its corresponding isotopic pattern, indicating 12 degrees of unsaturation. The structure of **4** was determined by detailed 1D (^{1}H) and 2D NMR (COSY, NOESY, HSQC and HMBC) spectroscopic analyses further assisted by comparison of its NMR data (Table S5) with those of already reported LRGs.[1, 2] Interpretation of the HSQC and HMBC spectra revealed the presence of eight quaternary carbons (including a low field carbonyl resonating at δ_C 194.5, an ester carbonyl at δ_C 169.9, five sp^2 carbons at δ_C 135.0, 140.0, 140.8, 147.0 and 153.2, and one sp^3 carbon at δ_C 49.0), six sp^2 methine carbons, one olefinic methylene carbon, two aliphatic sp^3 methines (one of them oxygenated), four aliphatic methylenes (one of them oxygenated) and two aliphatic methyl groups. Analysis of COSY correlations identified different spin systems which could be connected using the key long-range correlations observed in the HMBC spectrum (Figure S44). The spin system comprising H-10 to H-13, contains four olefinic protons corresponding to Δ^{10} *E* and Δ^{12} *Z* double bonds, as indicated by the measured coupling constants of 15.7 and 11.3 Hz, respectively. The Δ^{10} *E* double bond of this spin system is conjugated with an olefinic exomethylene group, as indicated by the key long-range correlations between H-11/C-9 and H-10/C-20. On the Δ^{12} *Z* double bond end, this spin system is conjugated with the aromatic oxazole heterocycle characteristic of LRGs, as revealed by the key HMBC correlations between H-15 and C-13, C-14 and C-16. The extended π-system between positions 9 and 16 is connected to another spin system, comprising H-7 and H-8, as demonstrated by the long-range correlations from H-10 to C-8 and from H-8 to C-9. The HMBC correlations of the methyl H-21 with C-5, C-6 and C-7 confirmed the expected substitution position of this methyl group. As expected, the chemical shifts from position 5 to 17 were indeed very similar to those of LRG O1[1] and LRGs K1-K4[2], which share identical connectivity in that segment. The spin system comprising H-17 and H-18 turned out to be identical to that present in

LRG C12. Again, key long-range correlations between H-18 and H-19 with the sp^2 C-17 at δ_C 140.8 confirmed the presence of the oxime functionality characteristic of native LRGs.[1] Similar to LRG C12, the key HMBC correlation between H-18 and C-1 indicates that macrocyclic ring closure takes place as an ester bond involving the oxygen at position C-18, accounting for the deshielded ^{13}C/^{1}H chemical shifts observed for the oxygenated methine at that position. The C-3 side chain spin system, comprised by H-22 (methine) and H-23 (methyl), revealed very unusual NMR features. The ^{1}H and ^{13}C chemical shifts of the methine group are surprisingly downfield (δ_H at 4.29 and δ_C 71.8) and both H-22 methine and H-23 methyl also correlate with an unexpectedly downfield carbonyl, C-24, at δ_C 194.5 that cannot correspond to a free carboxylic acid group, as otherwise usual in native LRGs.[1] A β-thiolactone moiety is required to meet the molecular formula and it perfectly accounts for these observations since it has been reported that both α-protons and α-carbons of β-propiothiolactones exhibit atypical downfield chemical shifts due to a through-space interaction between the occupied orbital of the α-carbon and the vacant orbital of sulfur.[4] Likewise, the thioester nature of such β-thiolactone also explains the unexpected downfield chemical shift of C-24 carbonyl. Thus, the sulfur atom substituent at C-3 is not part of a free thiol group but rather part of a thioester functionality. The structure of LRG C22 is very remarkable since no naturally occurring compound bearing a β-thiolactone moiety have been reported to date. Again, the absolute configuration of the chiral centers at C-3, C-18 and C-22 was assigned to be the same as for all previously reported LRGs, based on their common biosynthetic origin.[1, 2]

Largimycin C23 (**5**) was assigned a molecular formula $C_{24}H_{28}N_2O_6S$, identical to that of LRG C22, based on the observed ion $[M+H]^+$ at m/z 473.1741 (calcd. for $C_{24}H_{29}N_2O_6S^+$, 473.1741, Δ 0 ppm) alongside its corresponding isotopic pattern, indicating 12 degrees of unsaturation. The structure of **5** was determined by 1D (1H) and 2D NMR (COSY, NOESY, HSQC and HMBC) spectroscopic analyses further assisted by comparison of its NMR data (Table S6) with those of LRG C22. Interestingly, LRG C23 displayed identical NMR features and key COSY and long-range HMBC correlations as those found for LRG C22 (Figure S44). In agreement with these observations, LRG C23 shares the same connectivity determined for LRG C22 and displays almost identical chemical shifts for all positions but C-16, C-17 and C-18, that respectively resonate at δ_C 156.6, 144.8 and 67.8 (compared with δ_C 153.2, 140.8 and 75.4 in LRG C22). Using the chemical shifts of oxime α-carbons as diagnostic of the oxime double bond stereochemistry, it is observed that C-16 is significantly deshielded and C-18 is shielded in LRG C-23 in comparison with LRG C22. This observation indicates that LRG C23 presents an *E* stereochemistry for the oxime double bond.[5]

LRG C23 is the oxime *E* isomer of LRG C22. Obviously, LRG C23 and LRG C22 share identical absolute configuration for their chiral centers.

Largimycin C24 (**6**) was assigned a molecular formula $C_{24}H_{28}N_2O_6S$, identical to that of LRG C22 and LRG C23, based on the observed ion $[M+H]^+$ at *m/z* 473.1749 (calcd. for $C_{24}H_{29}N_2O_6S^+$, 473.1741, Δ 1.7 ppm) alongside its corresponding isotopic pattern, indicating 12 degrees of unsaturation. The structure of **6** was determined by 1D (^{1}H) and 2D NMR (COSY, NOESY, HSQC and HMBC) spectroscopic analyses further assisted by comparison of its NMR data (Table S7) with those of LRG C22. Interestingly, LRG C24 displayed identical NMR features and key COSY and long-range HMBC correlations as those found for LRG C22 with the sole exception that the long-range correlation that provides the site of macrocycle ring closure is observed between H-19 and C-1 (Figure S44). Thus, LRG C24 displays identical connectivity and stereochemistry of the olefinic double bonds and the oxime as LRG C22 just differing in the position of esterification, that correspond to the primary hydroxy group in LRG C24. The absolute configuration for the chiral centers in LRG C24 is proposed to identical to that of LRG C23 and LRG C22 based on their shared biosynthetic origin. LRG C24 can be considered a regioisomer, regarding the macrocyclic ring-closing esterification position, of LRG C22.

Largimycin C25 (**7**) was assigned a molecular formula $C_{24}H_{28}N_2O_6S$, identical to that of LRG C22, LRG C23 and LRG C24, based on the observed ion $[M+H]^+$ at m/z 473.1746 (calcd. for $C_{24}H_{29}N_2O_6S^+$, 473.1741, Δ 1.1 ppm) alongside its corresponding isotopic pattern, indicating 12 degrees of unsaturation. The structure of **6** was determined by 1D (1H) and 2D NMR (COSY, NOESY, HSQC and HMBC) spectroscopic analyses further assisted by comparison of its NMR data (Table S8) with those of LRG C24. Interestingly, LRG C25 displayed identical NMR features and key COSY and long-range HMBC correlations as those found for LRG C24 (Figure S44). In agreement with these observations, LRG C25 shares the same connectivity determined for LRG C24 and displays almost identical chemical shifts for all positions but C-16, C-17 and C-18, that respectively resonate at δ_C 157.3, 146.8 and 65.3 (compared with δ_C 154.6, 142.7 and 69.0 in LRG C24). Once again, using the chemical shifts of oxime α-carbons as diagnostic of the oxime double bond stereochemistry, it is observed that C-16 is significantly deshielded and C-18 is shielded in LRG C-25 in comparison with LRG C24. This observation indicates that LRG C25 presents an *E* stereochemistry for the oxime double bond.[5] LRG C25 is the oxime *E* isomer of LRG C24. Obviously, LRG C25 and LRG C24 share identical absolute configuration for their chiral centers based on their common biosynthetic origin.

Figure S1. **Generation and genetic confirmation of *S. argillaceus* ΔlrgC2 mutant.** (A) Graphical representation of the generation of the mutant strain; (B) PCR analysis of *S. argillaceus* ΔlrgC2 mutant strain. PCR products from the wild type (WT) strain and from *S. argillaceus* ΔlrgC2 mutant strain using oligonucleotides Cit26 c up (a)/Cit26 c rp (b). C2.1 to C2.4 correspond to four independent mutants. λ, Pst-digested Lambda DNA; WT, wild type strain; *aac(3)IV*, apramycin resistance gene.

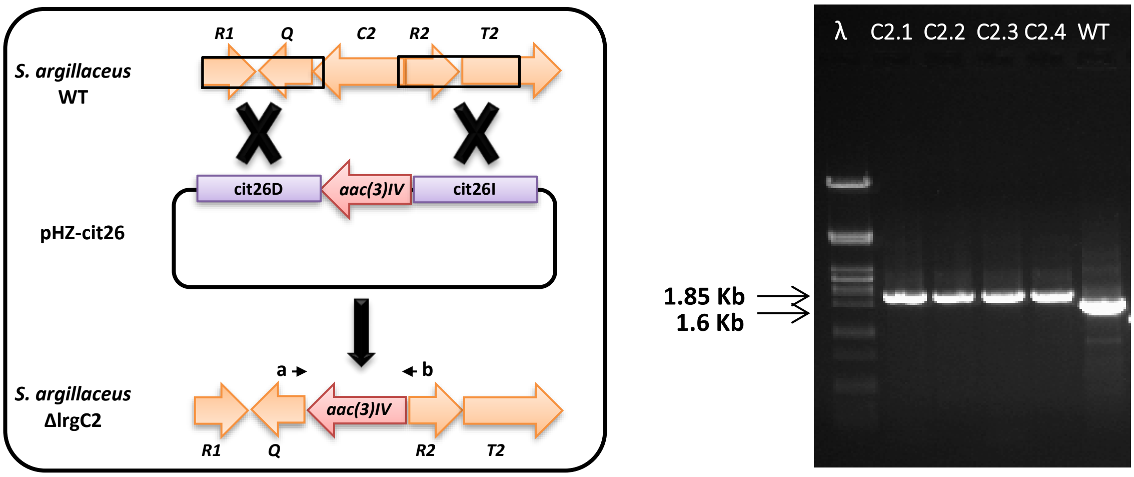

Figure S2: UPLC analyses of extracts of complemented *S. argillaceus* ΔlrgC2 mutant. Chromatogram at 330 nm of extracts from complemented mutant *S. argillaceus* ΔlrgC2-R2-C2 (in red) in comparison to *S. argillaceus* ΔlrgC2-R2 mutant (in black). Peak corresponding to LRG A2 is indicated.

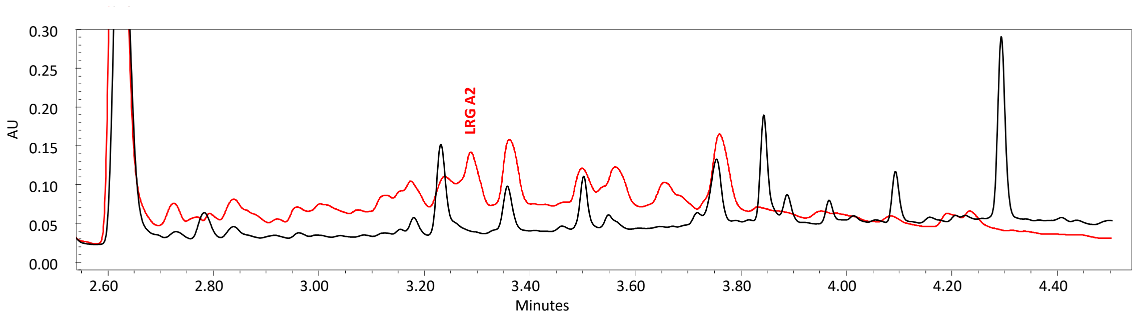

Figure S3. (A) UV-DAD spectrum of LRG C11 (**1**). (B) HRMS spectrum of LRG C11 (**1**).

A

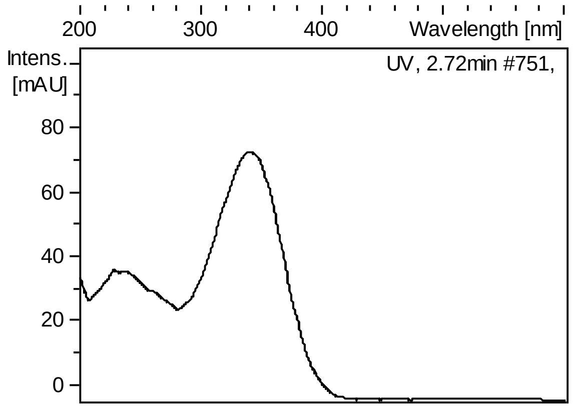

B

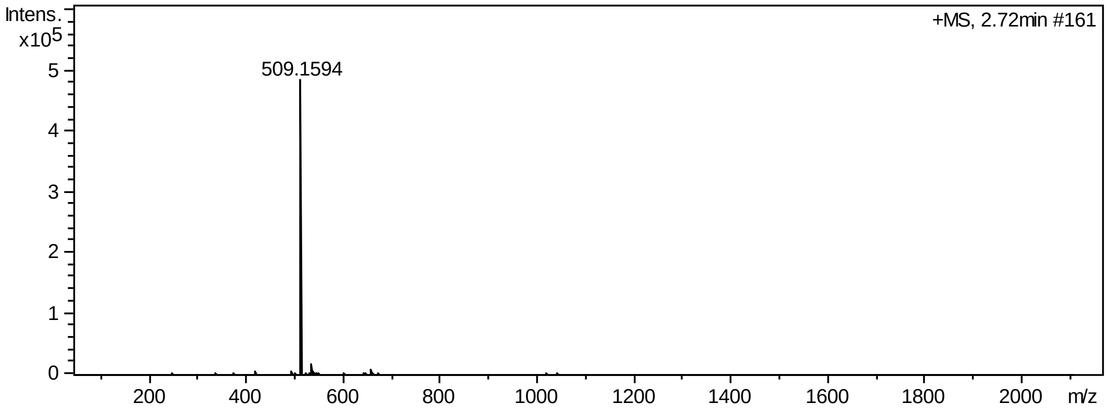

Figure S4. ^{1}H NMR spectrum of LRG C11 (**1**) (500 MHz, 24 ºC).

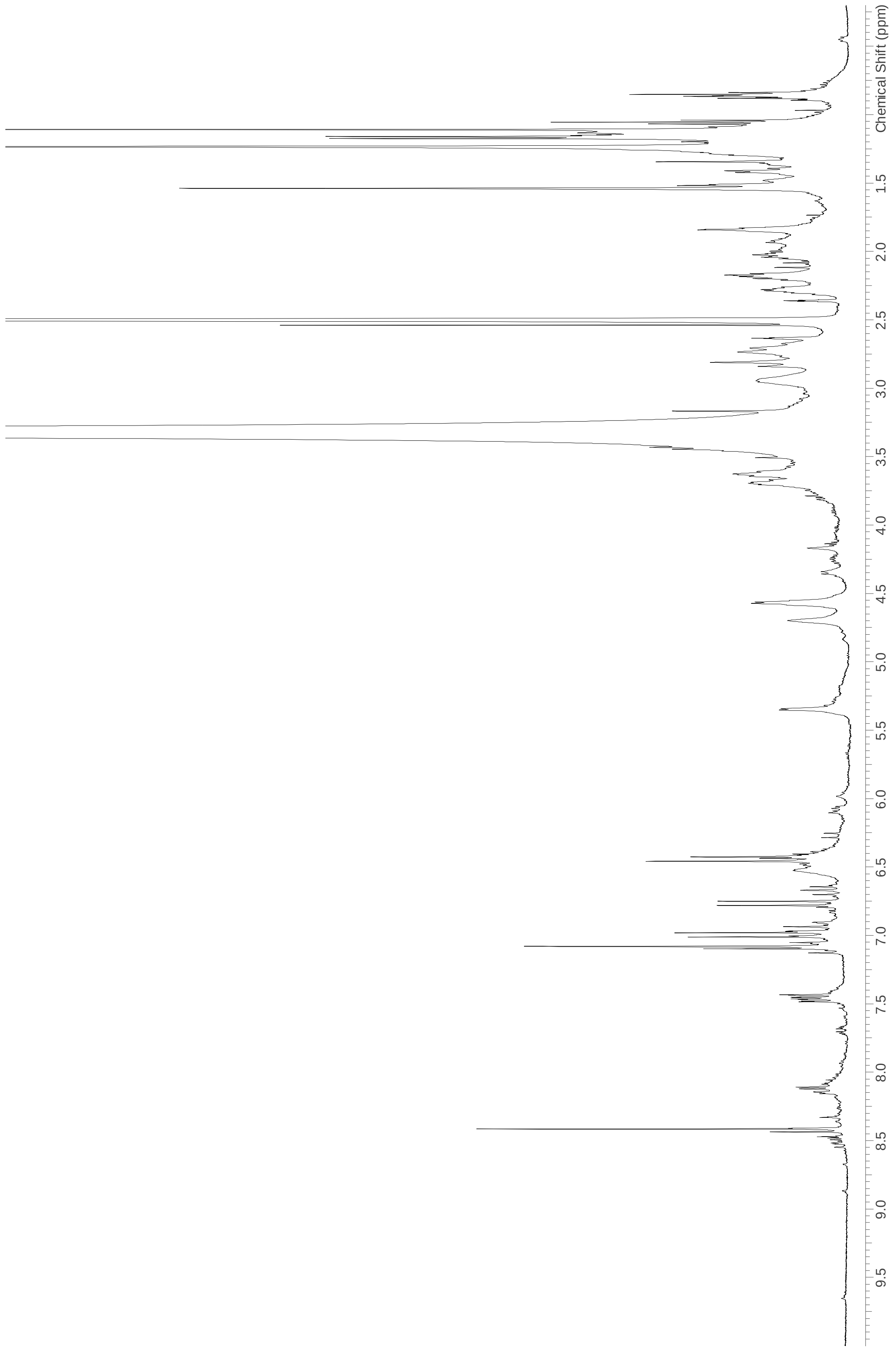

Figure S5. COSY spectrum of LRG C11 (**1**).

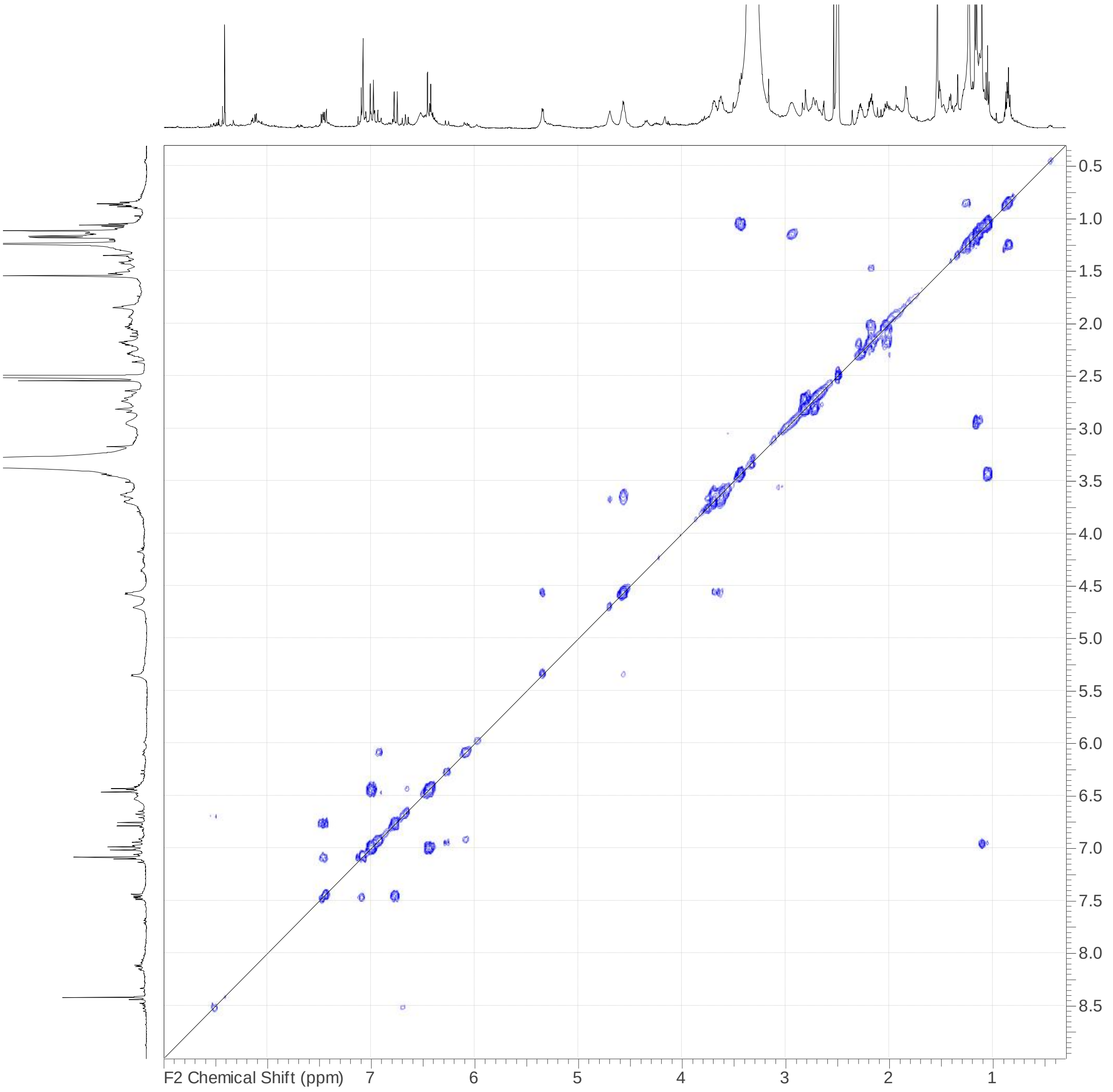

Figure S6. Edited HSQC spectrum of LRG C11 (**1**).

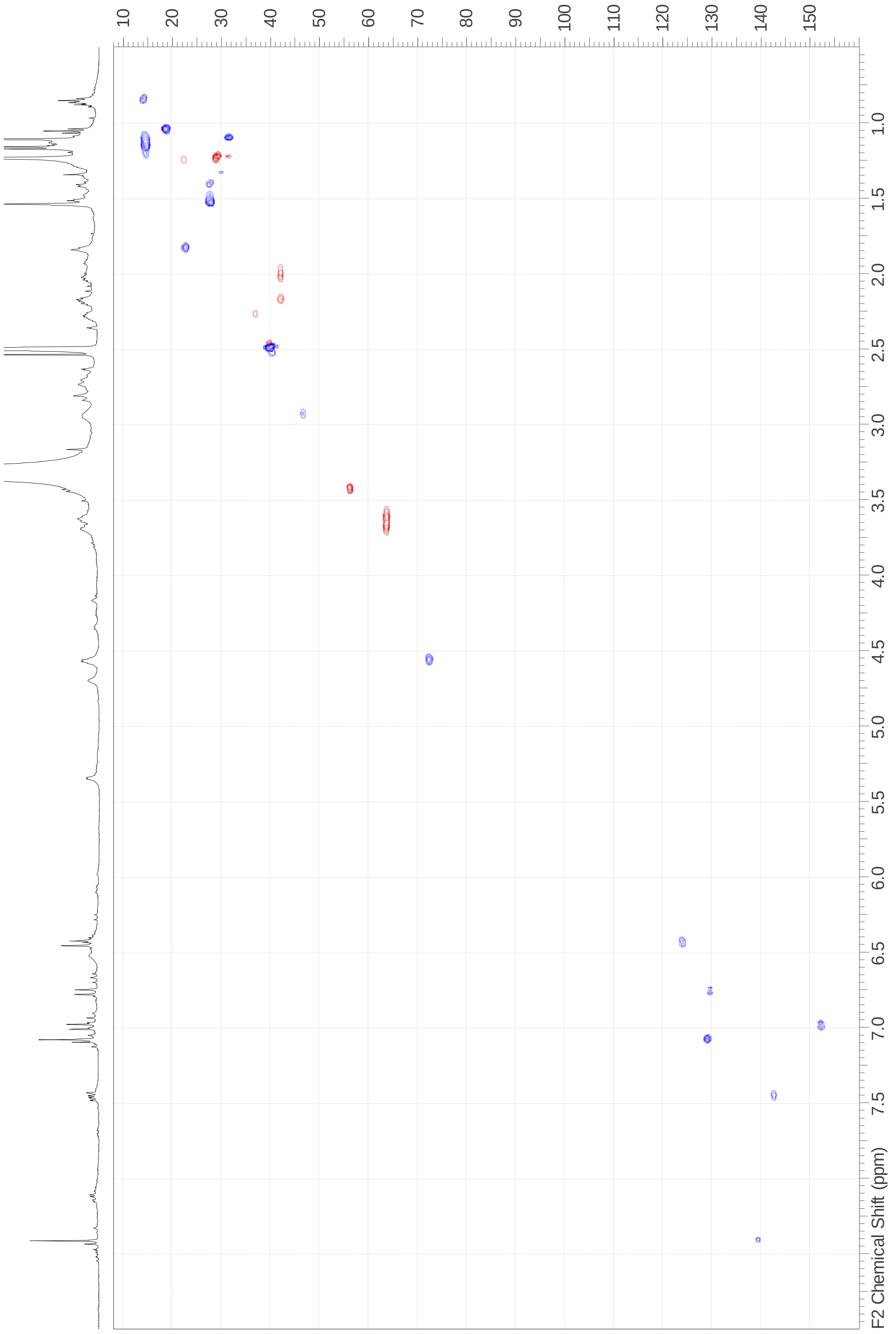

Figure S7. HMBC spectrum of LRG C11 (**1**).

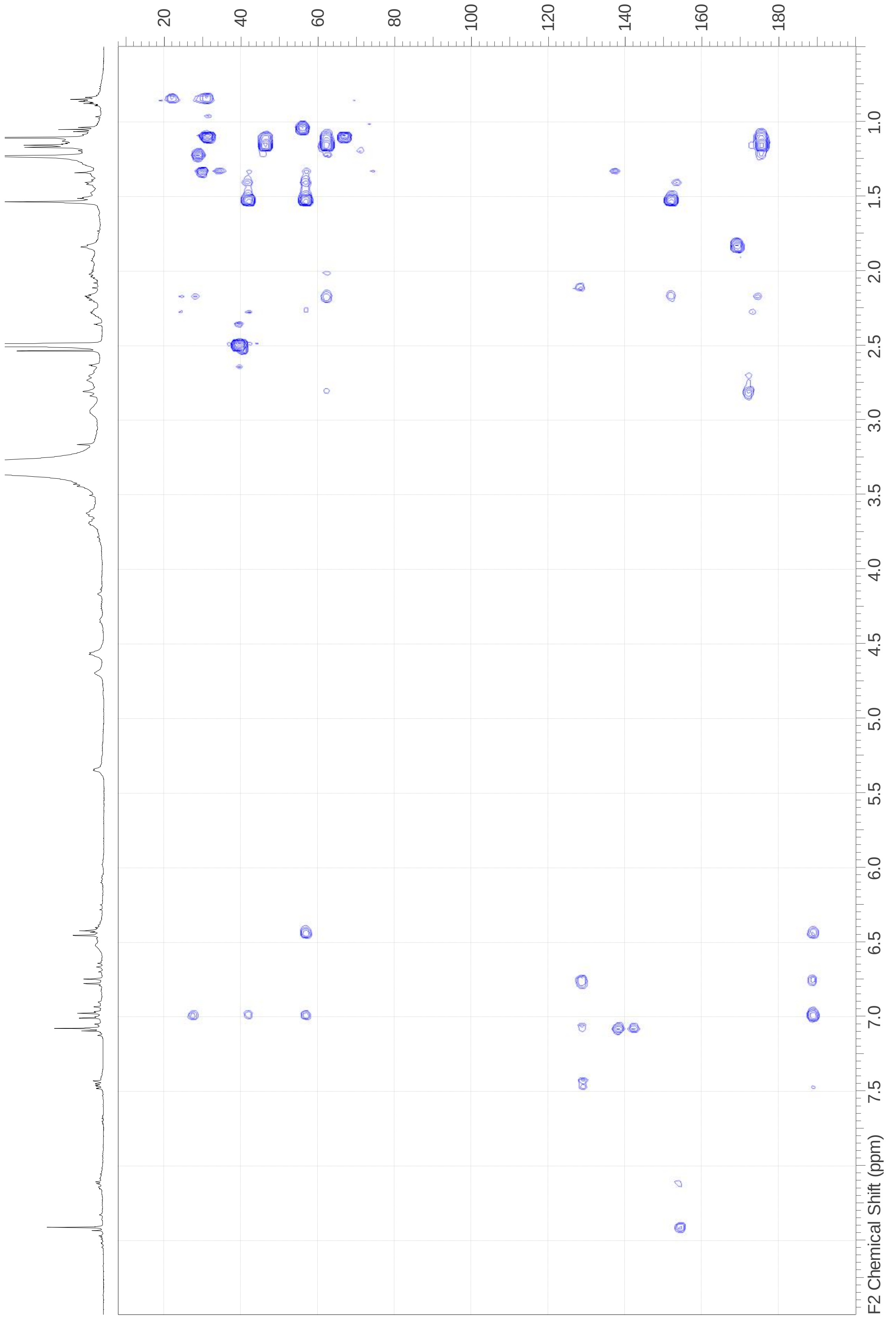

Figure S8. (A) UV-DAD spectrum of LRG C12a (**2**). (B) HRMS spectrum of LRG C12a (**2**).

A

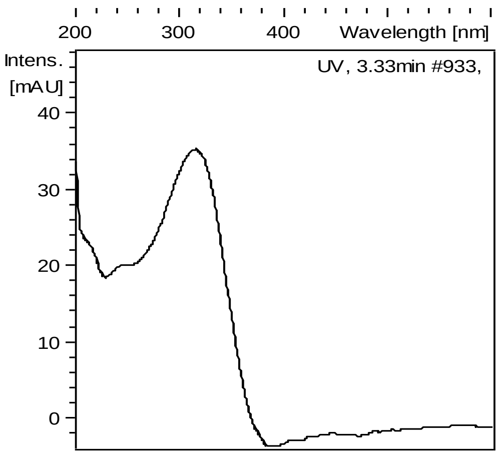

B

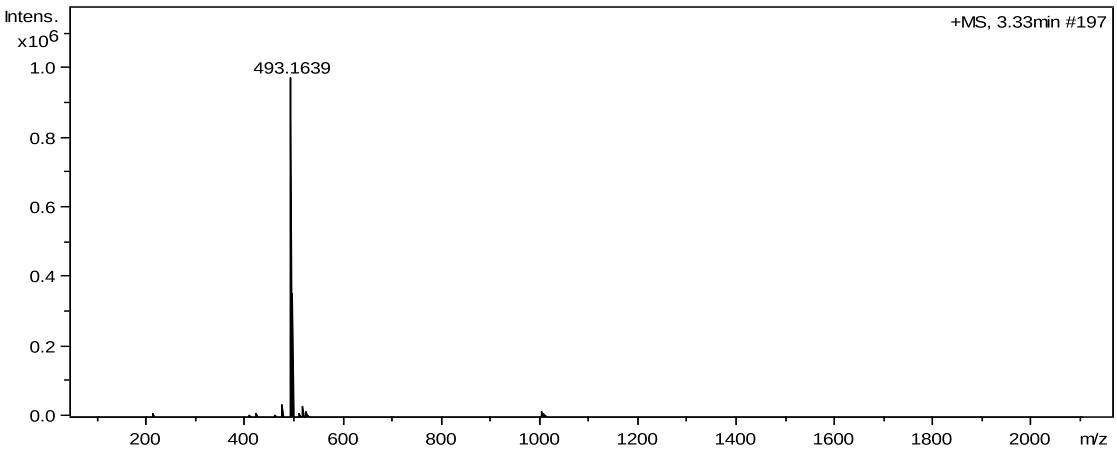

Figure S9. ^{1}H NMR spectrum of LRG C12a (**2**) (500 MHz, 24 ºC).

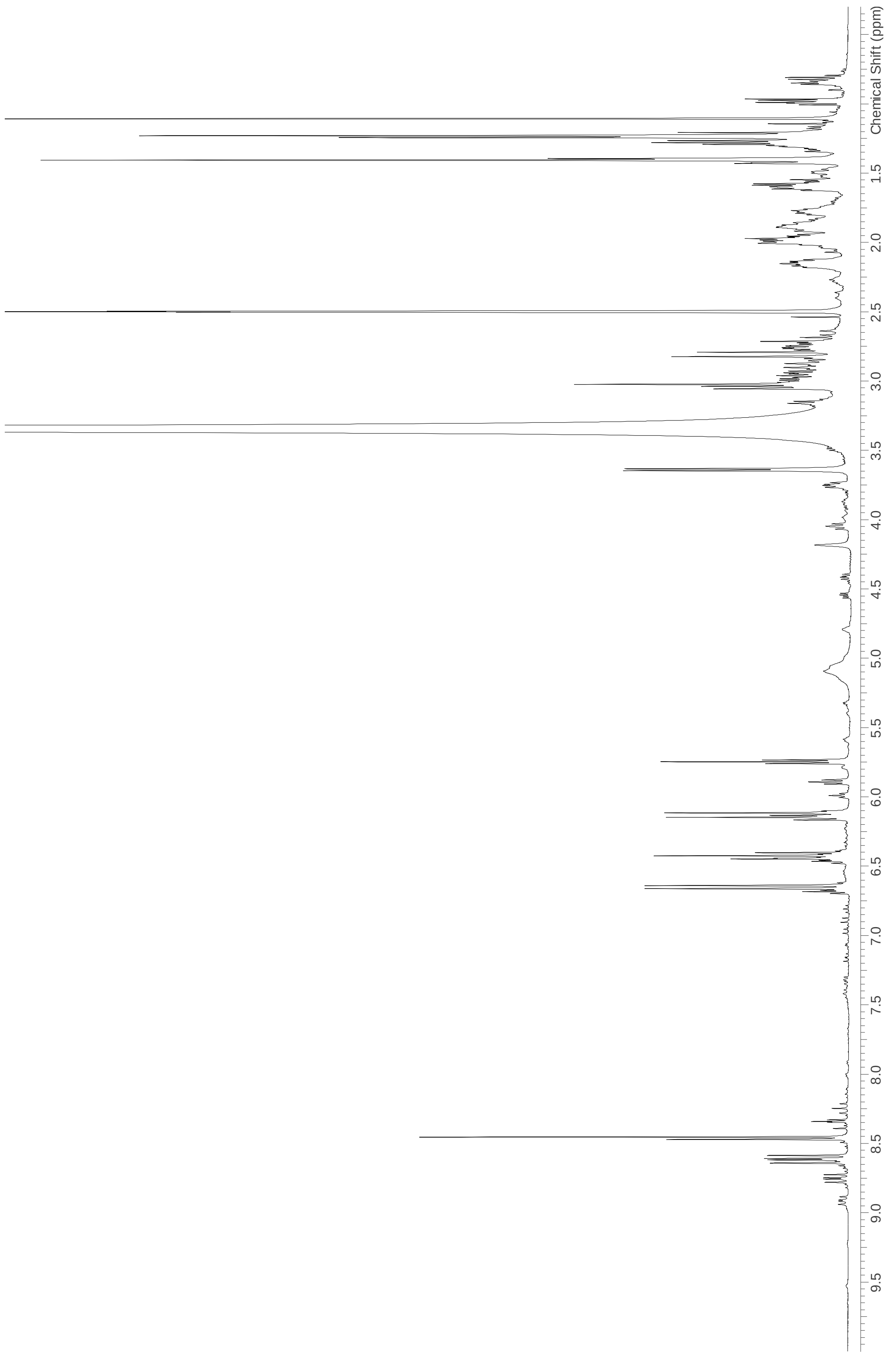

Figure S10. COSY spectrum of LRG C12a (**2**).

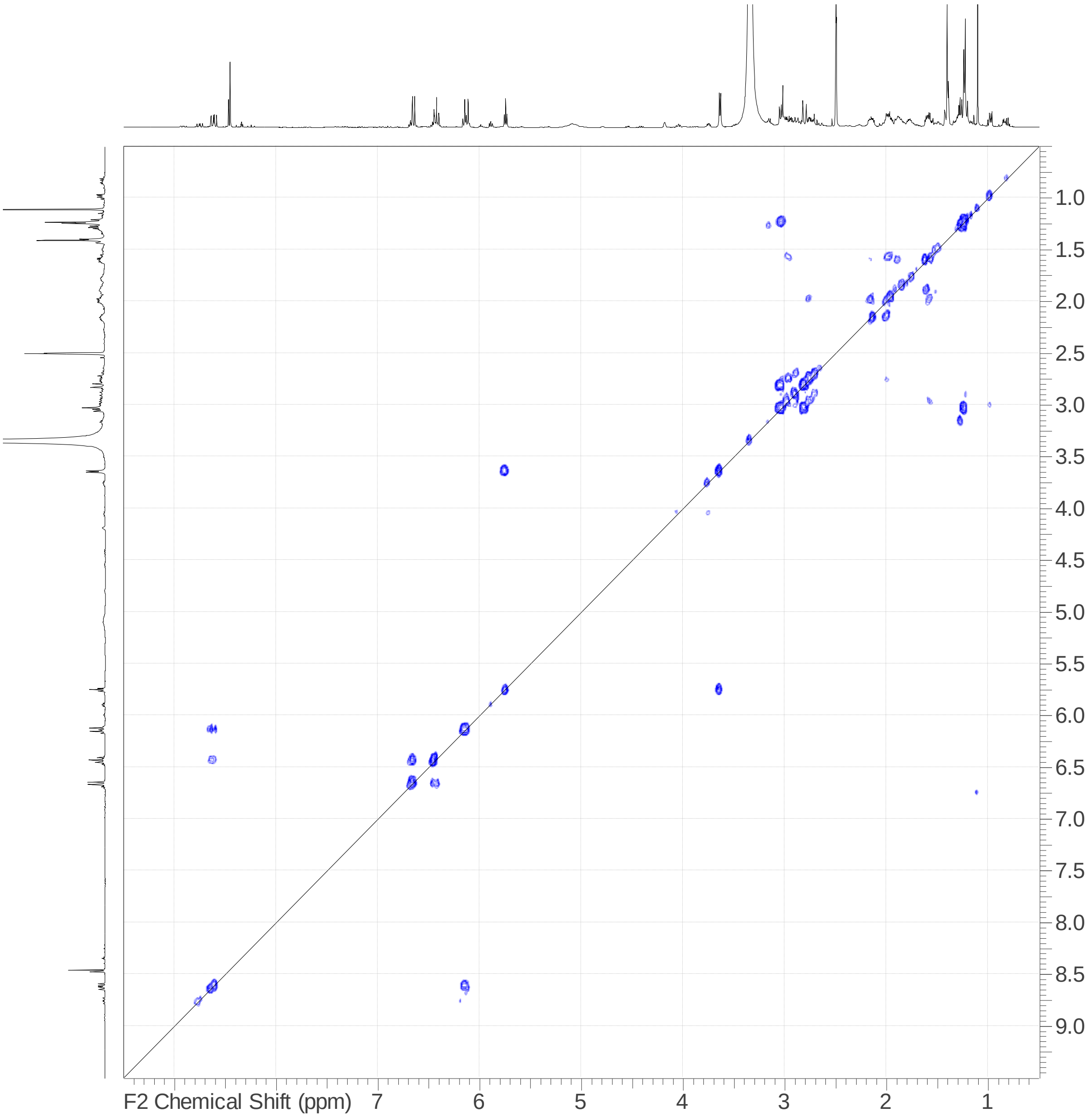

Figure S11. ROESY spectrum of LRG C12a (**2**).

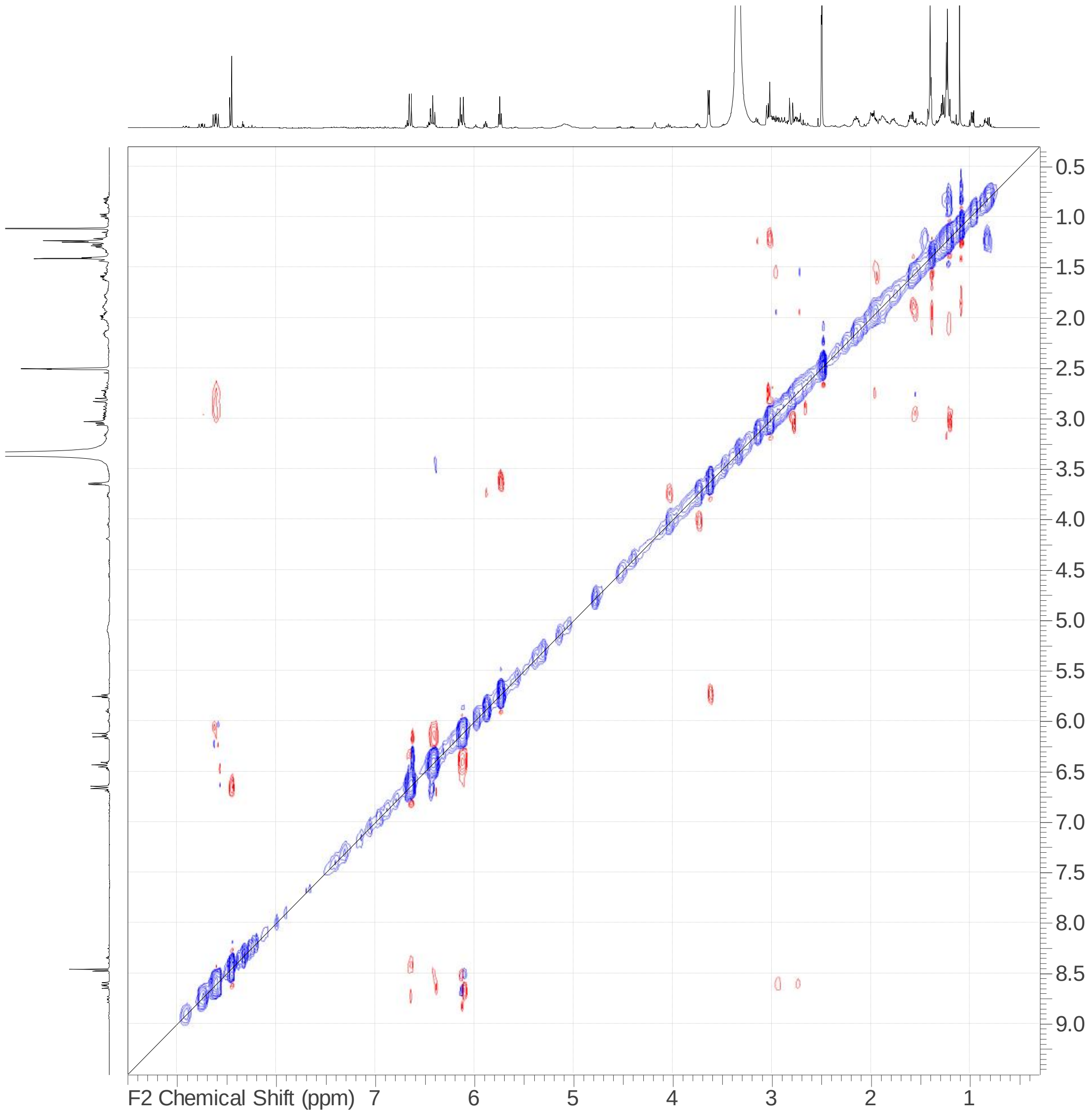

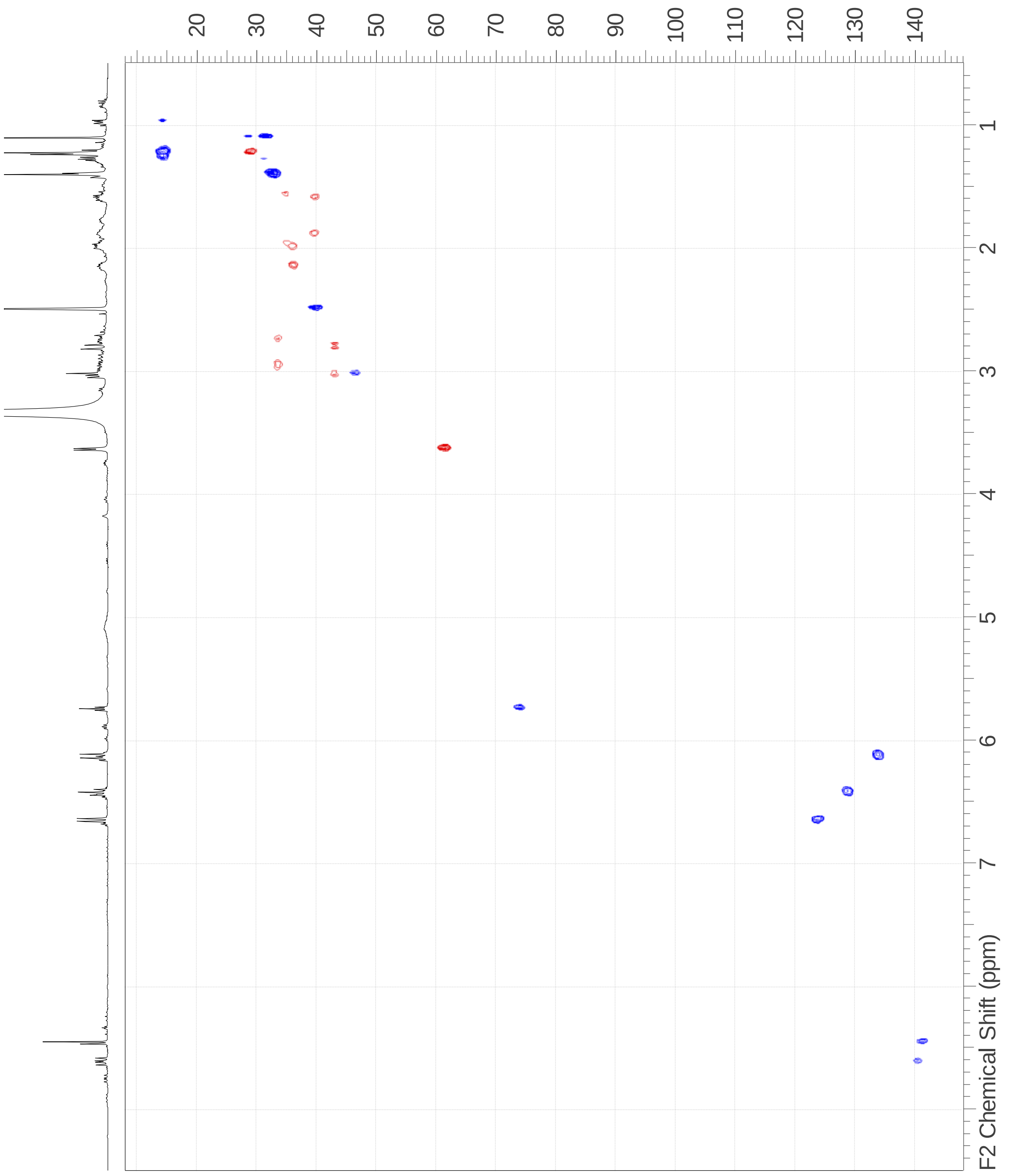

Figure S12. Edited HSQC spectrum of LRG C12a (**2**).

Figure S13. HMBC spectrum of LRG C12a (**2**).

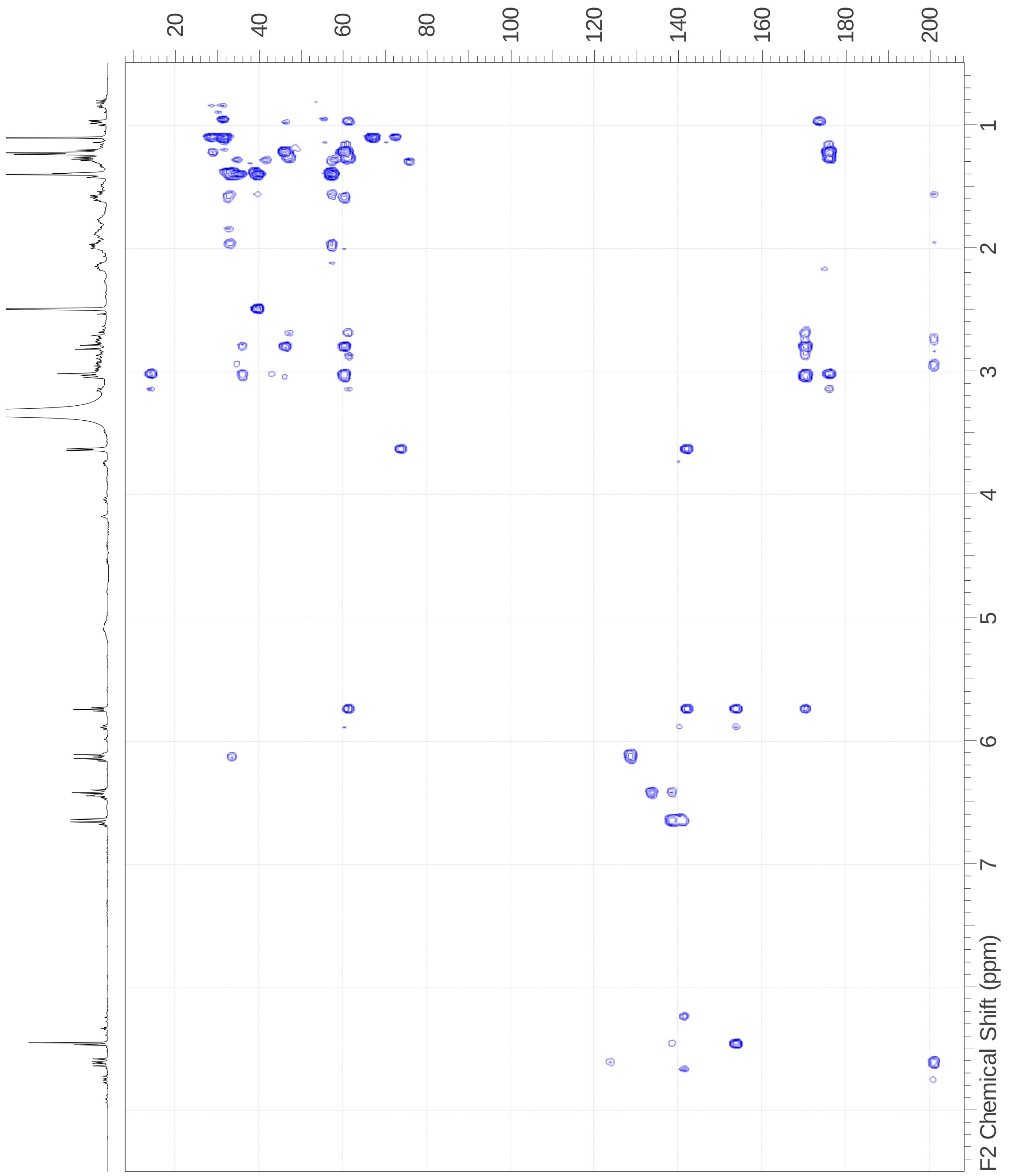

Figure S14. (A) UV-DAD spectrum of LRG C12b (**3**). (B) HRMS spectrum of LRG C12b (**3**).

A

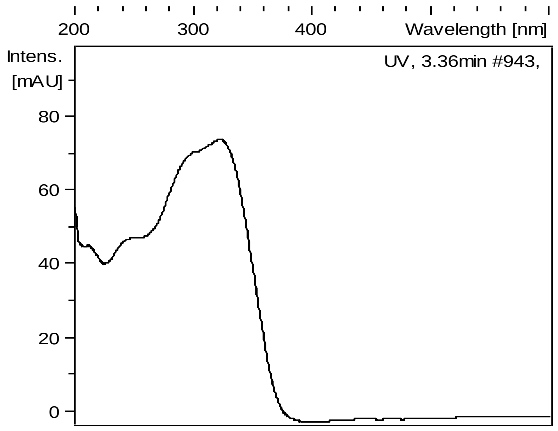

B

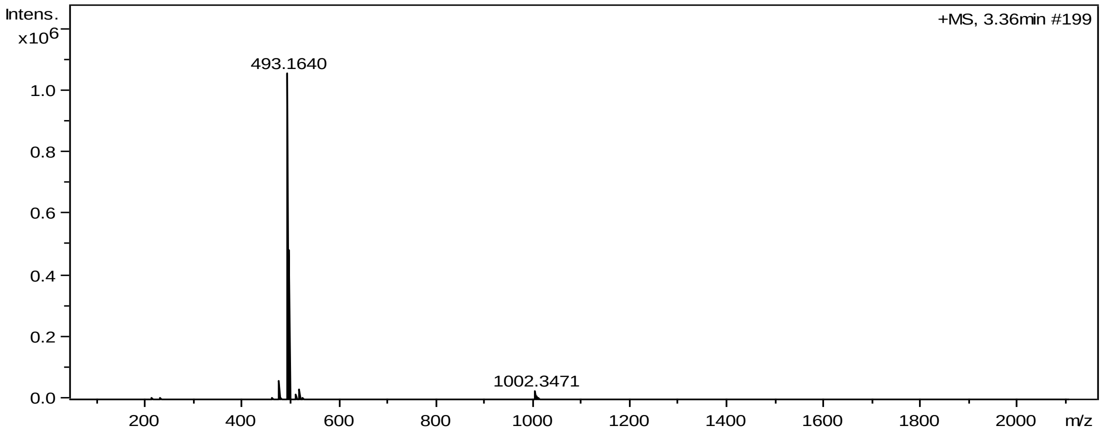

Figure S15. ^{1}H NMR spectrum of LRG C12b (**3**) (500 MHz, 24 ºC).

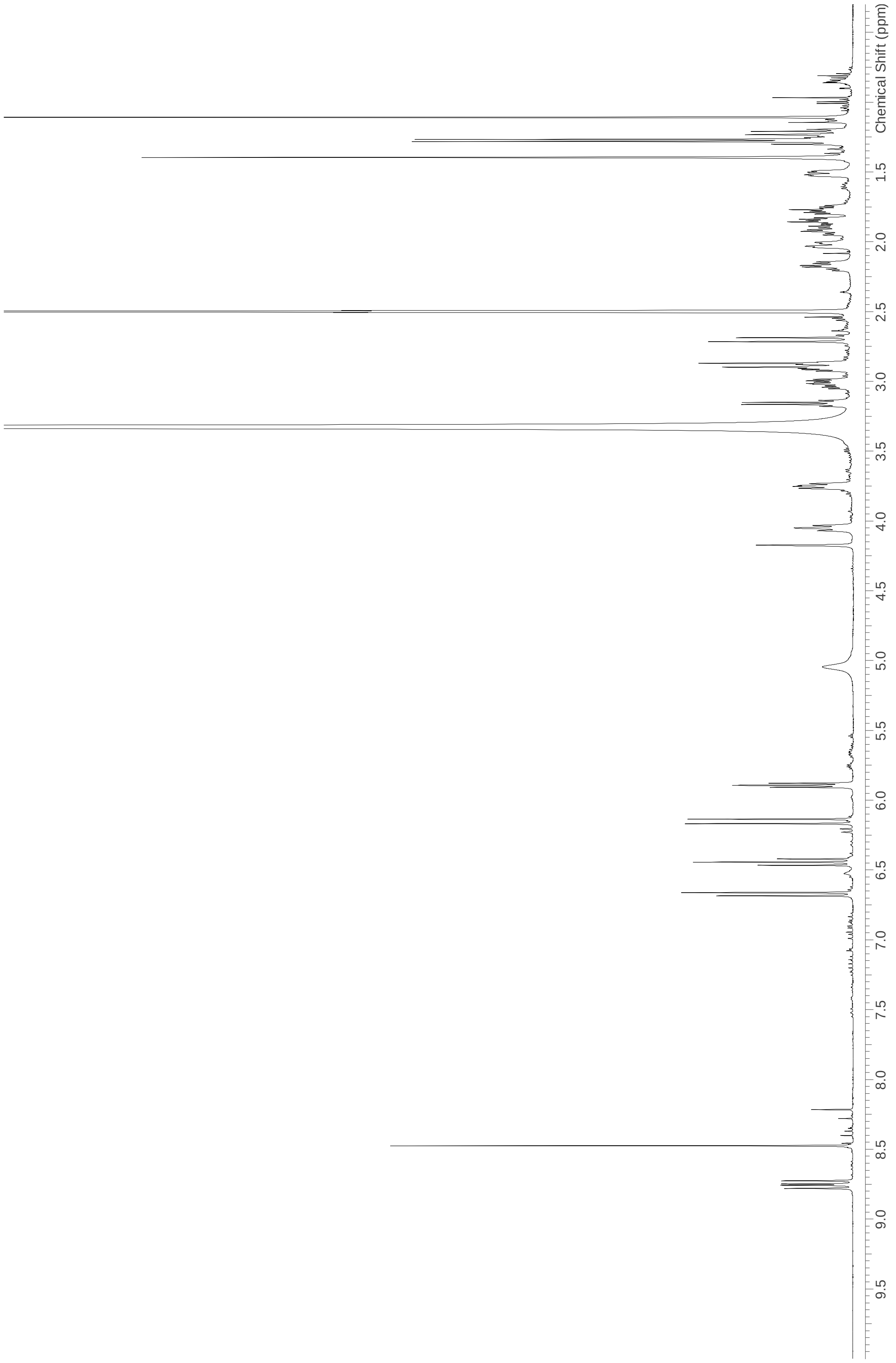

Figure S16. COSY spectrum of LRG C12b (**3**).

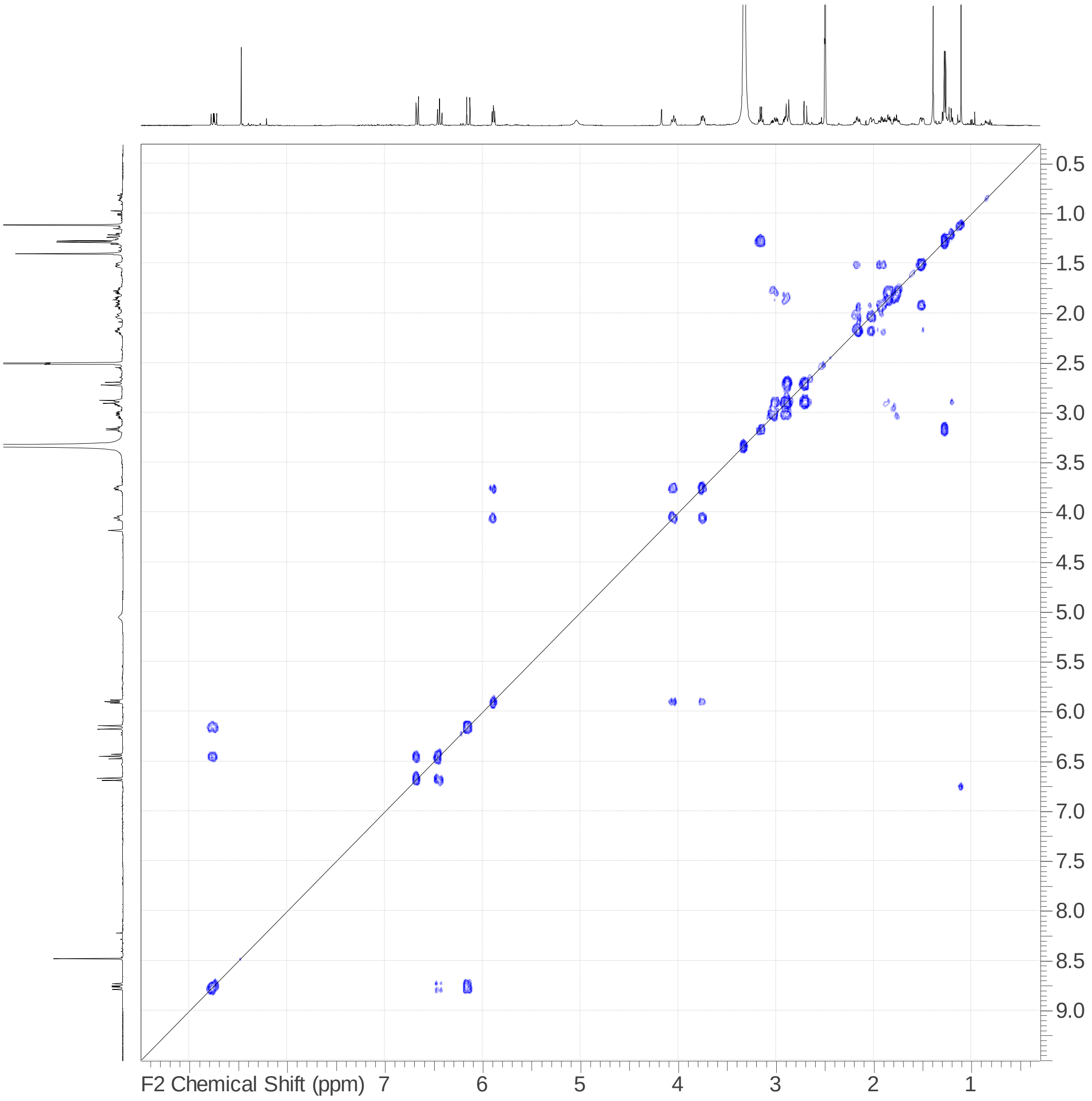

Figure S17. ROESY spectrum of LRG C12b (**3**).

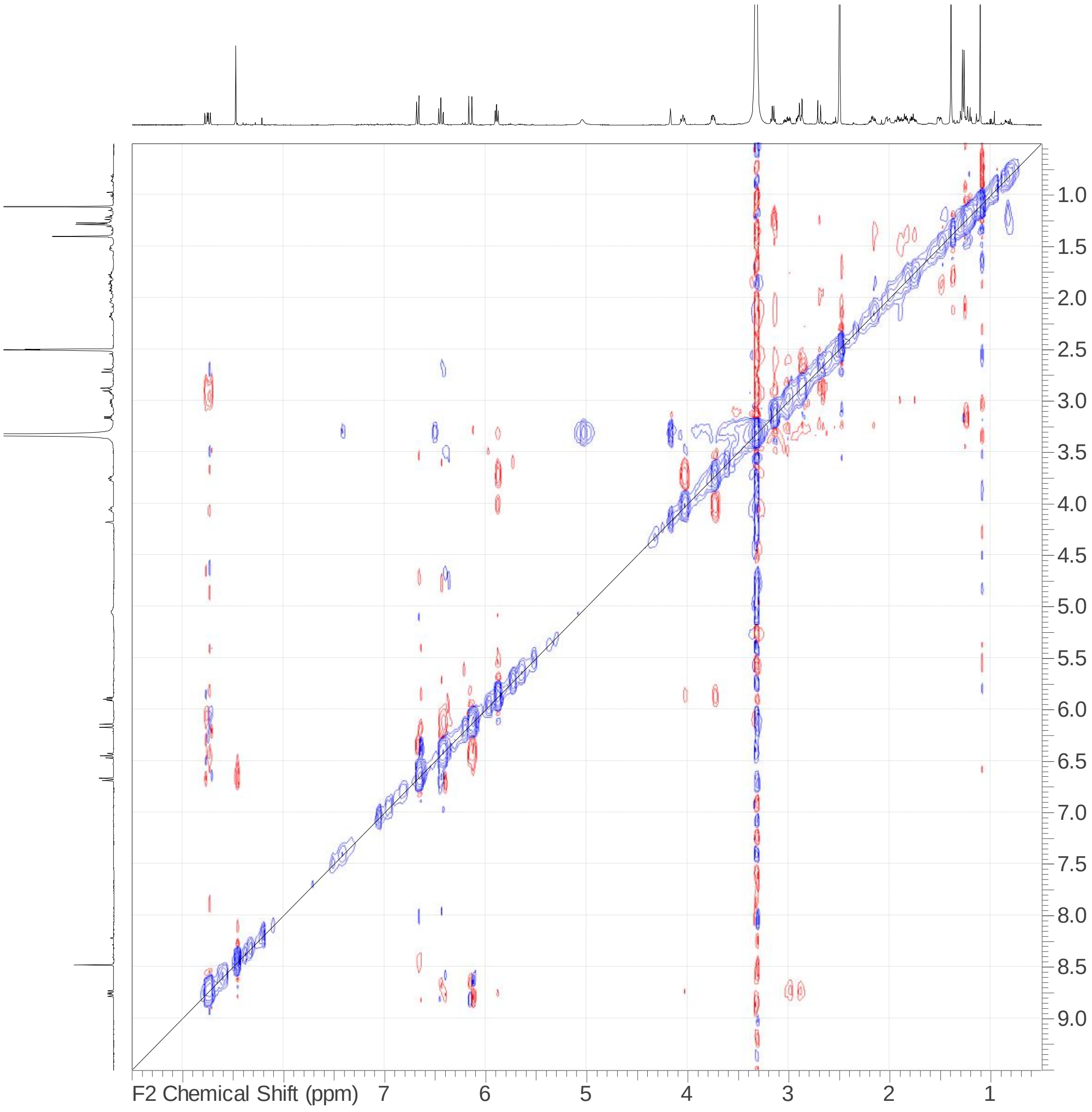

Figure S18. Edited HSQC spectrum of LRG C12b (**3**).

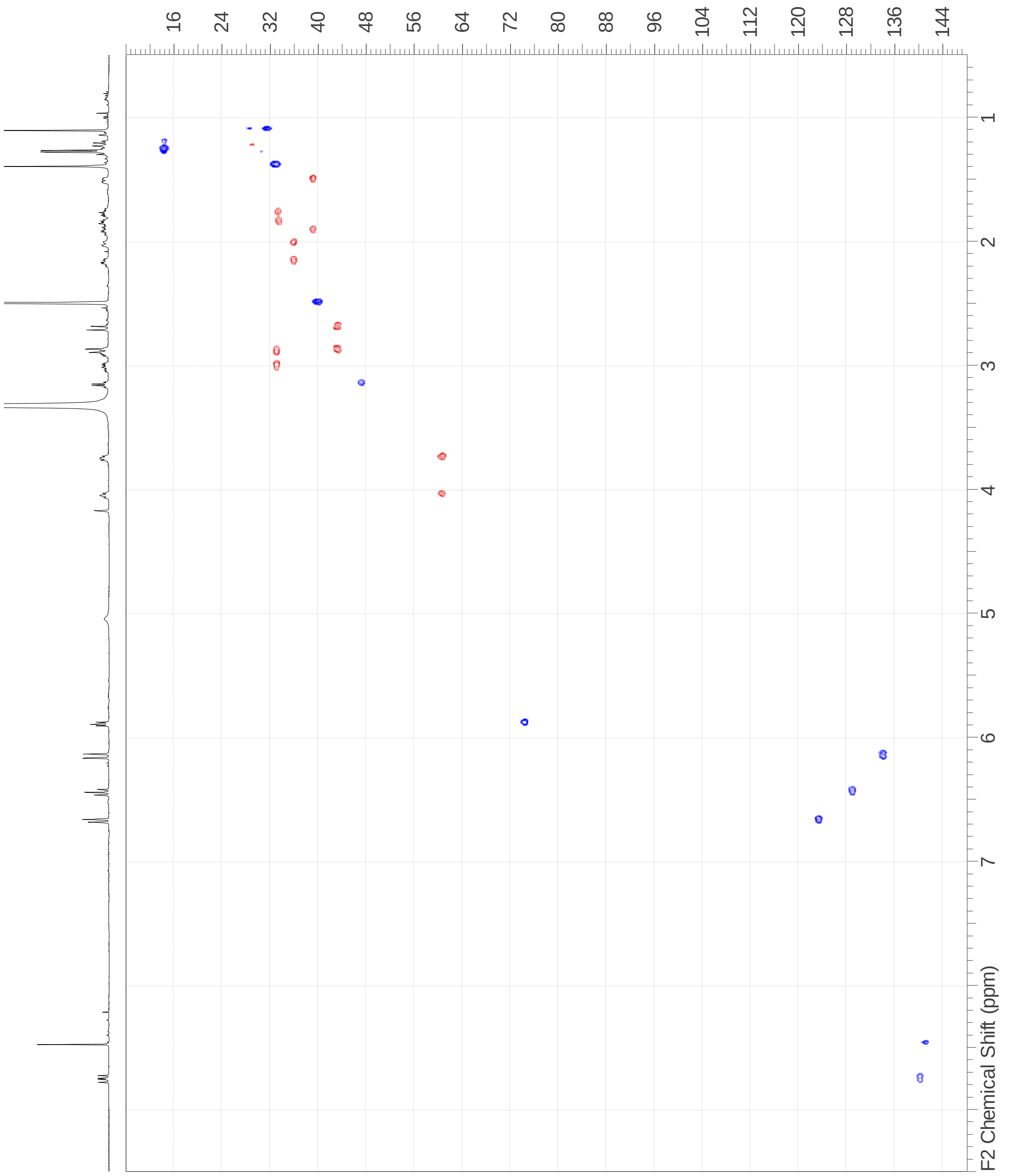

Figure S19. HMBC spectrum of LRG C12b (**3**).

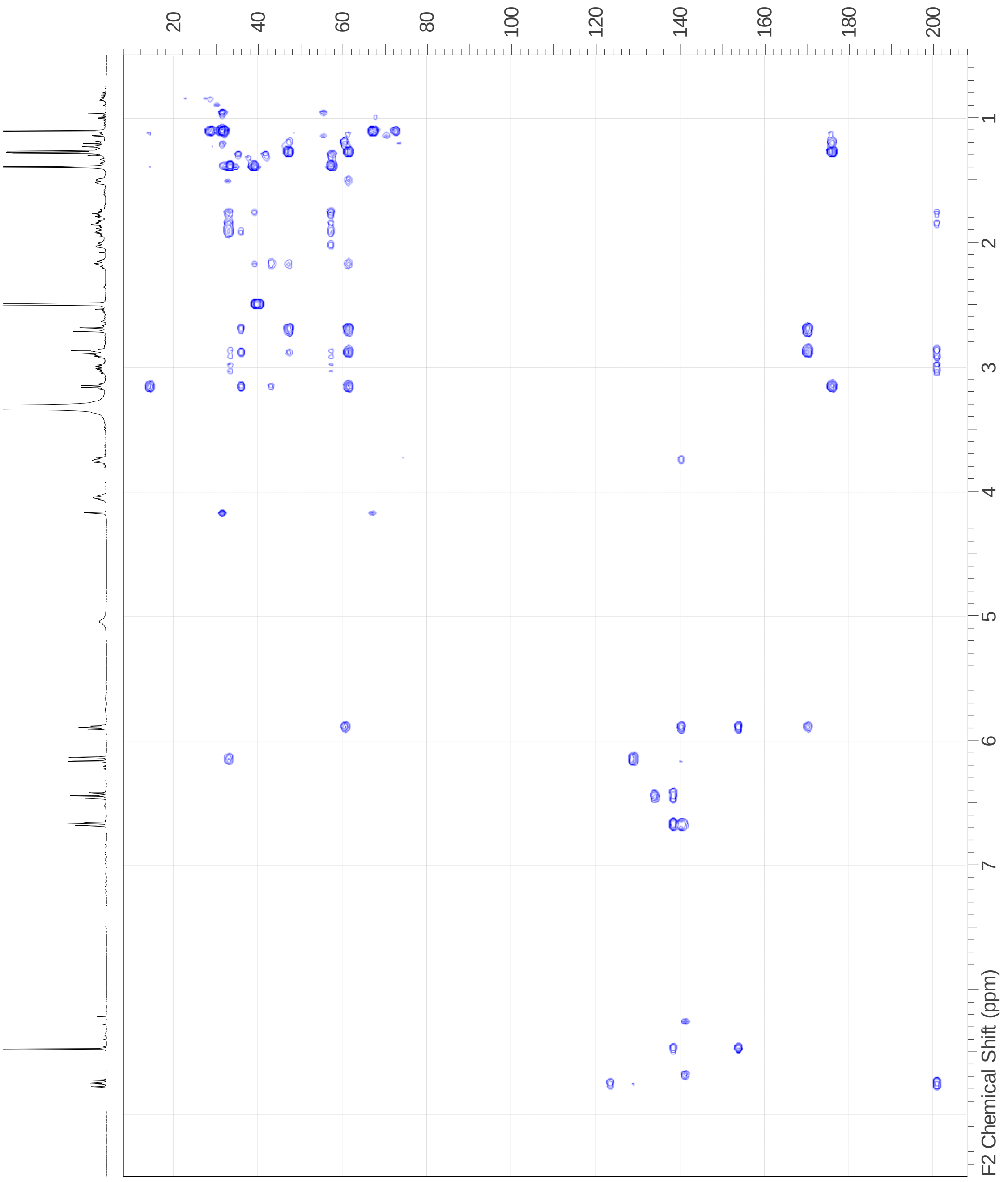

Figure S20. (A) UV-DAD spectrum of LRG C22 (**4**). (B) HRMS spectrum of LRG C22 (**4**).

A

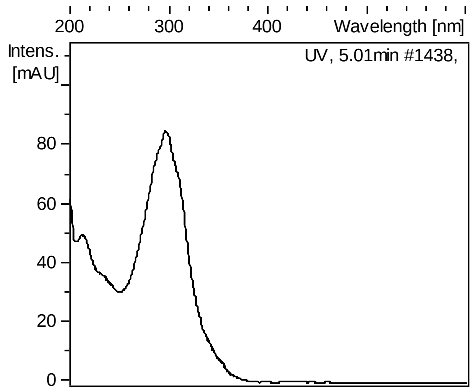

B

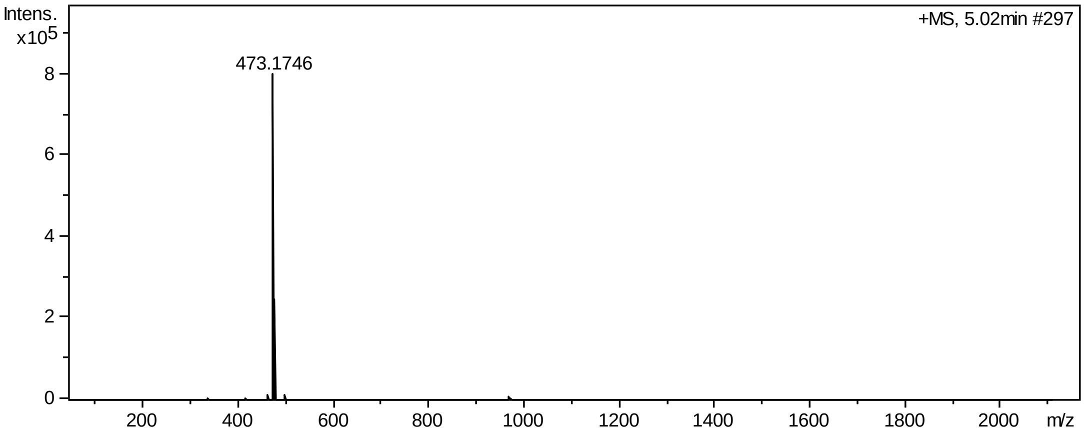

Figure S21. ^{1}H NMR spectrum of LRG C22 (**4**) (500 MHz, 24 ºC).

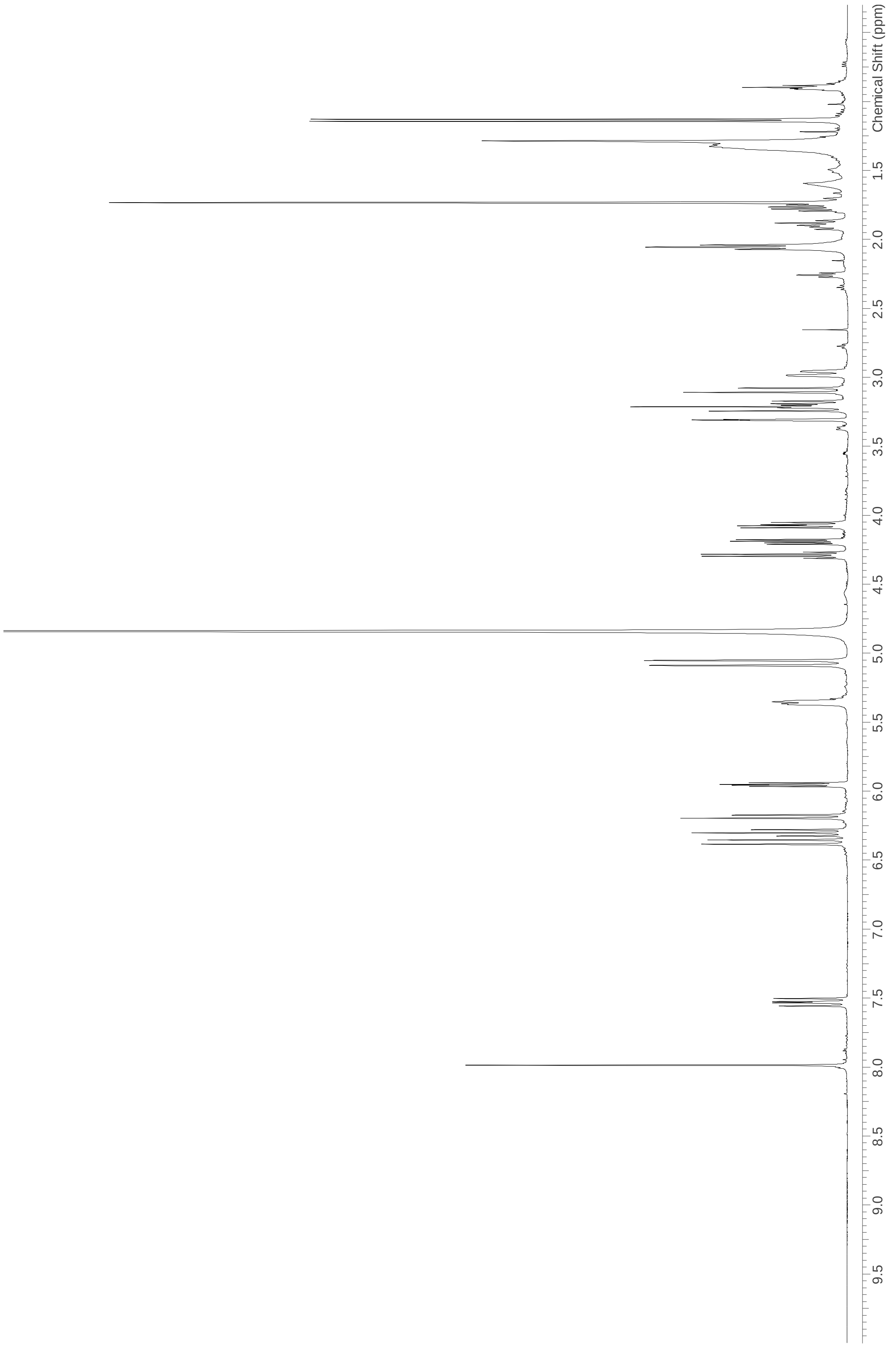

Figure S22. COSY spectrum of LRG C22 (**4**).

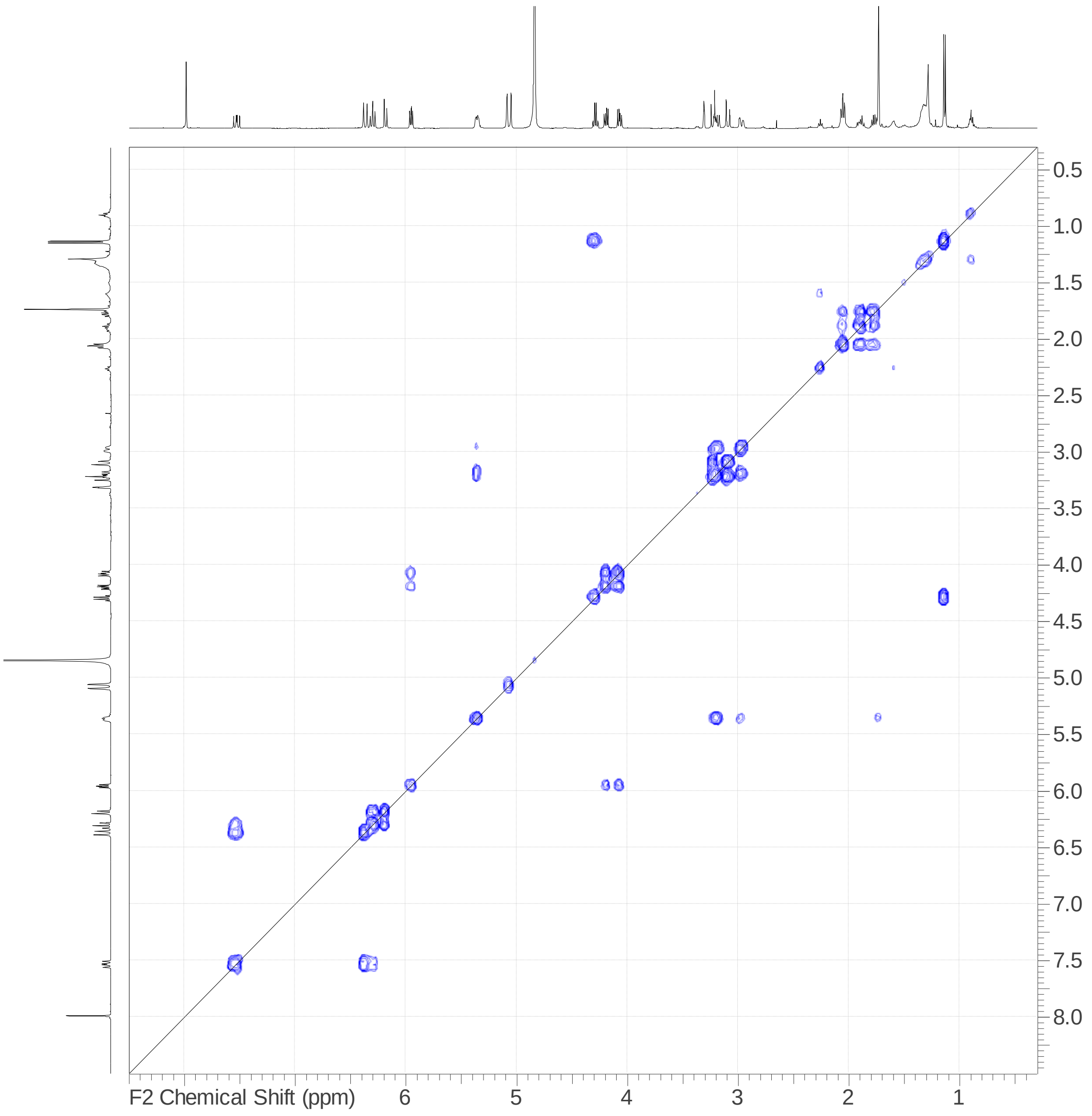

Figure S23. NOESY spectrum of LRG C22 (**4**).

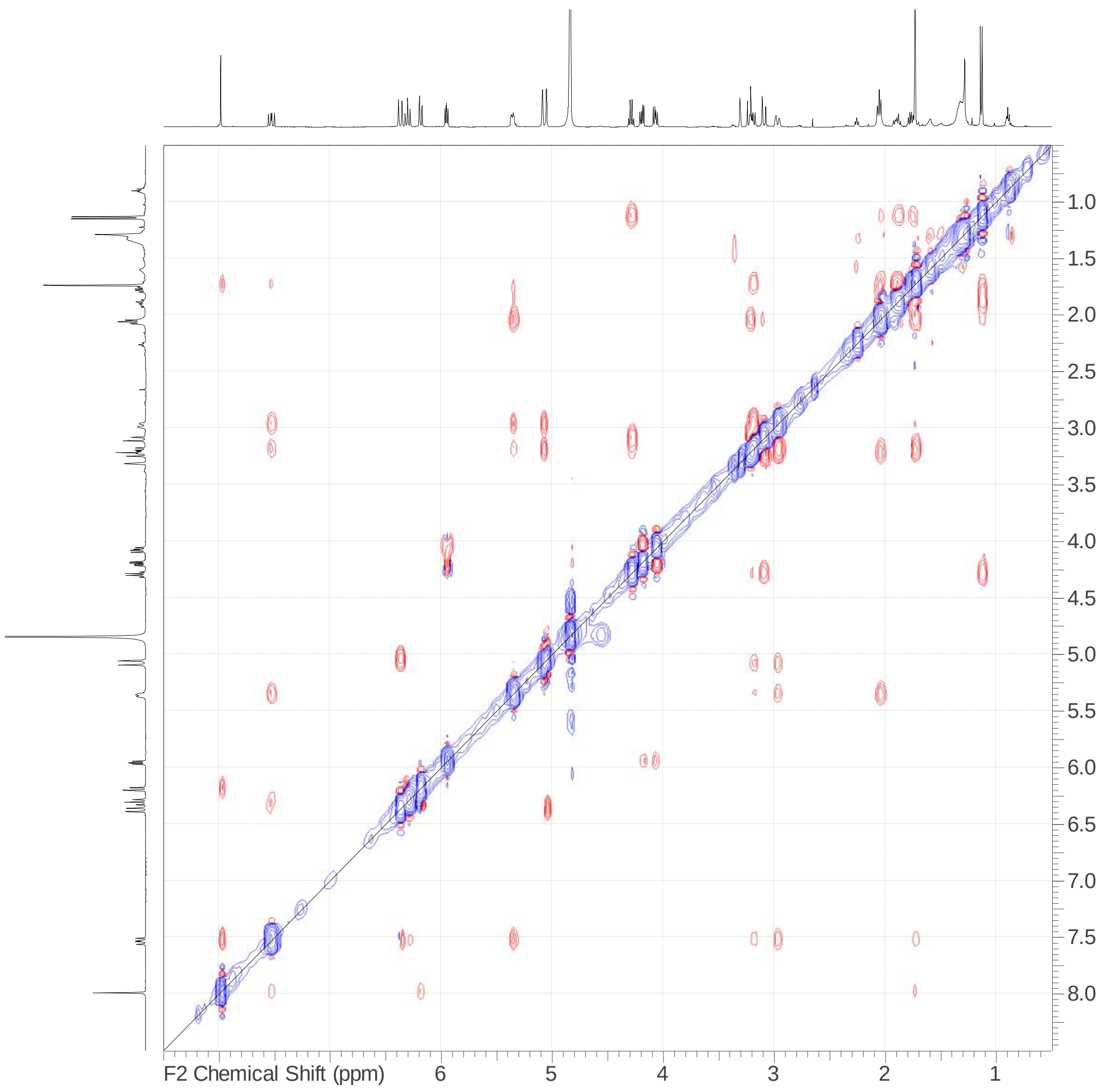

Figure S24. Edited HSQC spectrum of LRG C22 (**4**).

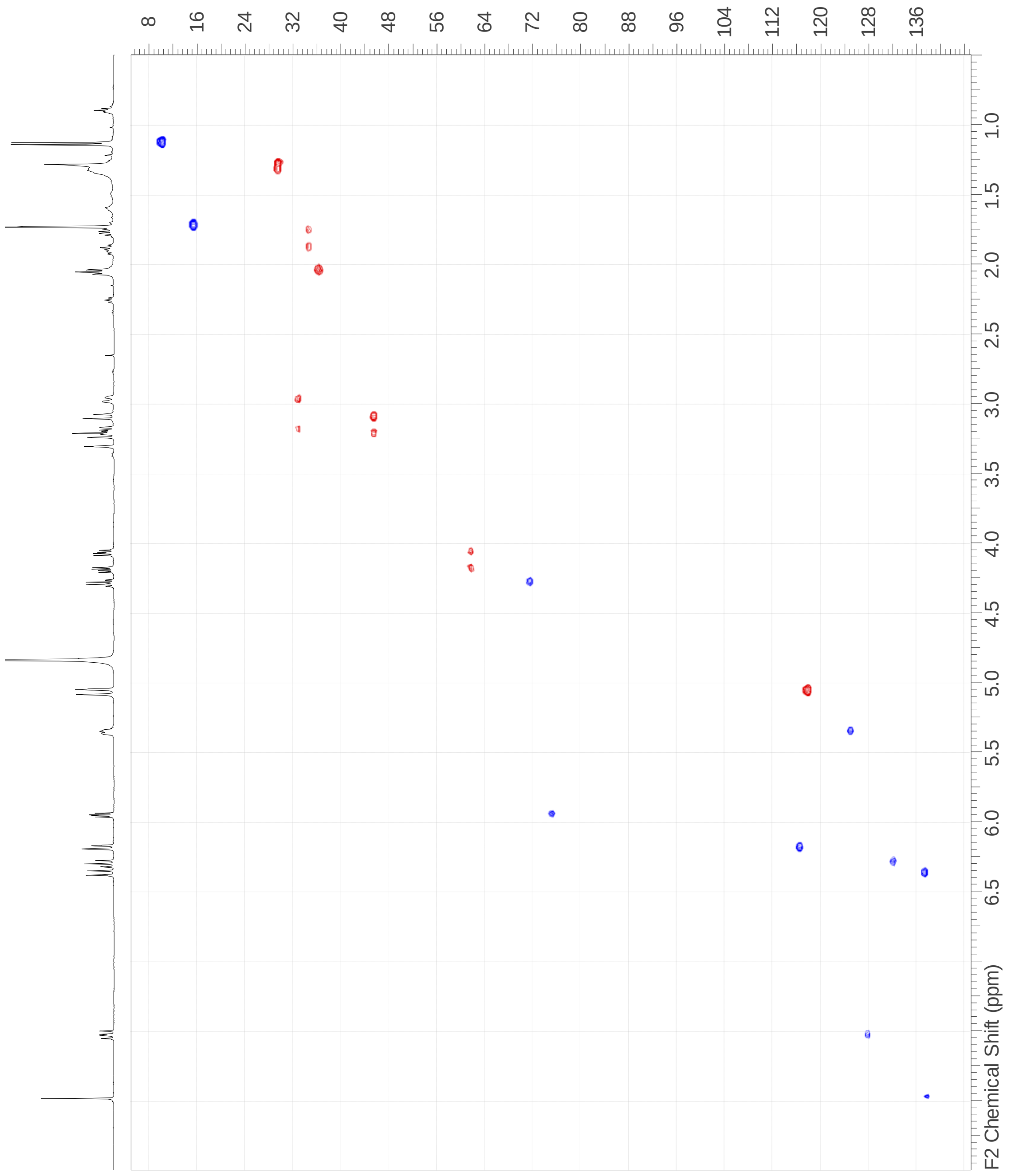

Figure S25. HMBC spectrum of LRG C22 (**4**).

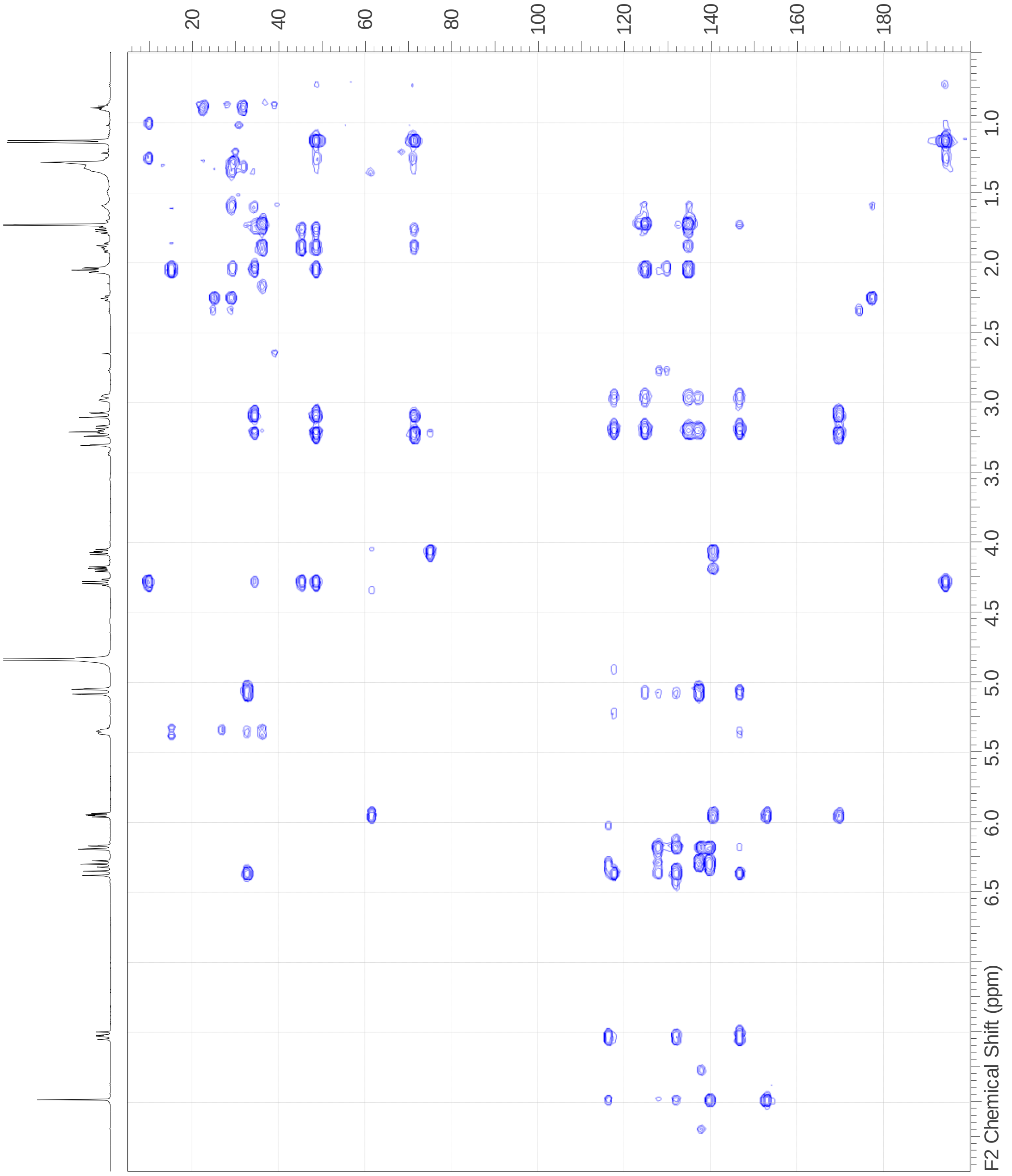

Figure S26. (A) UV-DAD spectrum of LRG C23 (**5**). (B) HRMS spectrum of LRG C23 (**5**).

A

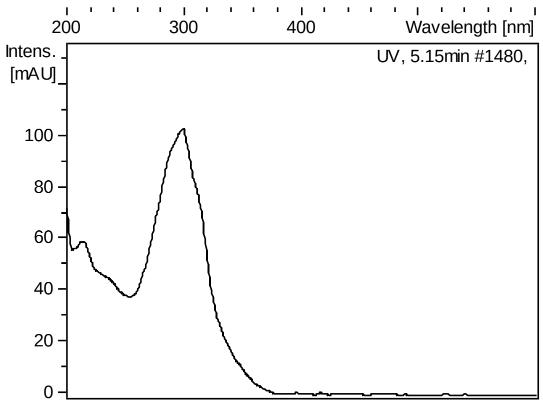

B

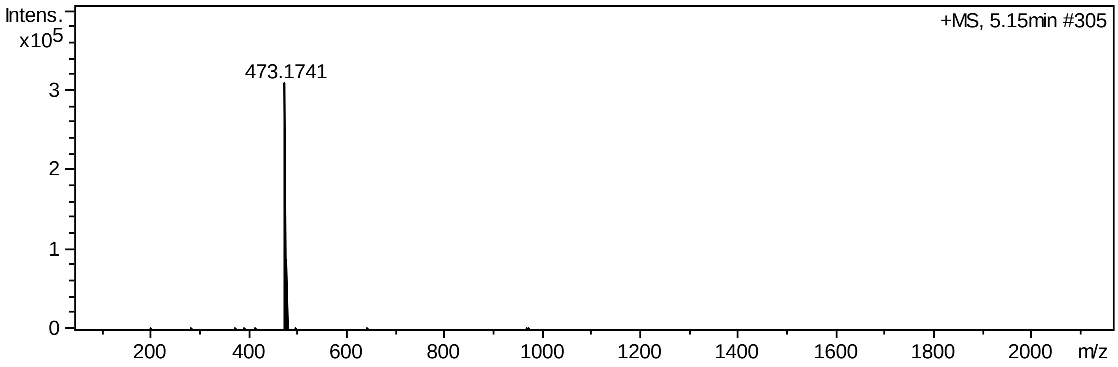

Figure S27. ^{1}H NMR spectrum of LRG C23 (**5**) (500 MHz, 24 ºC).

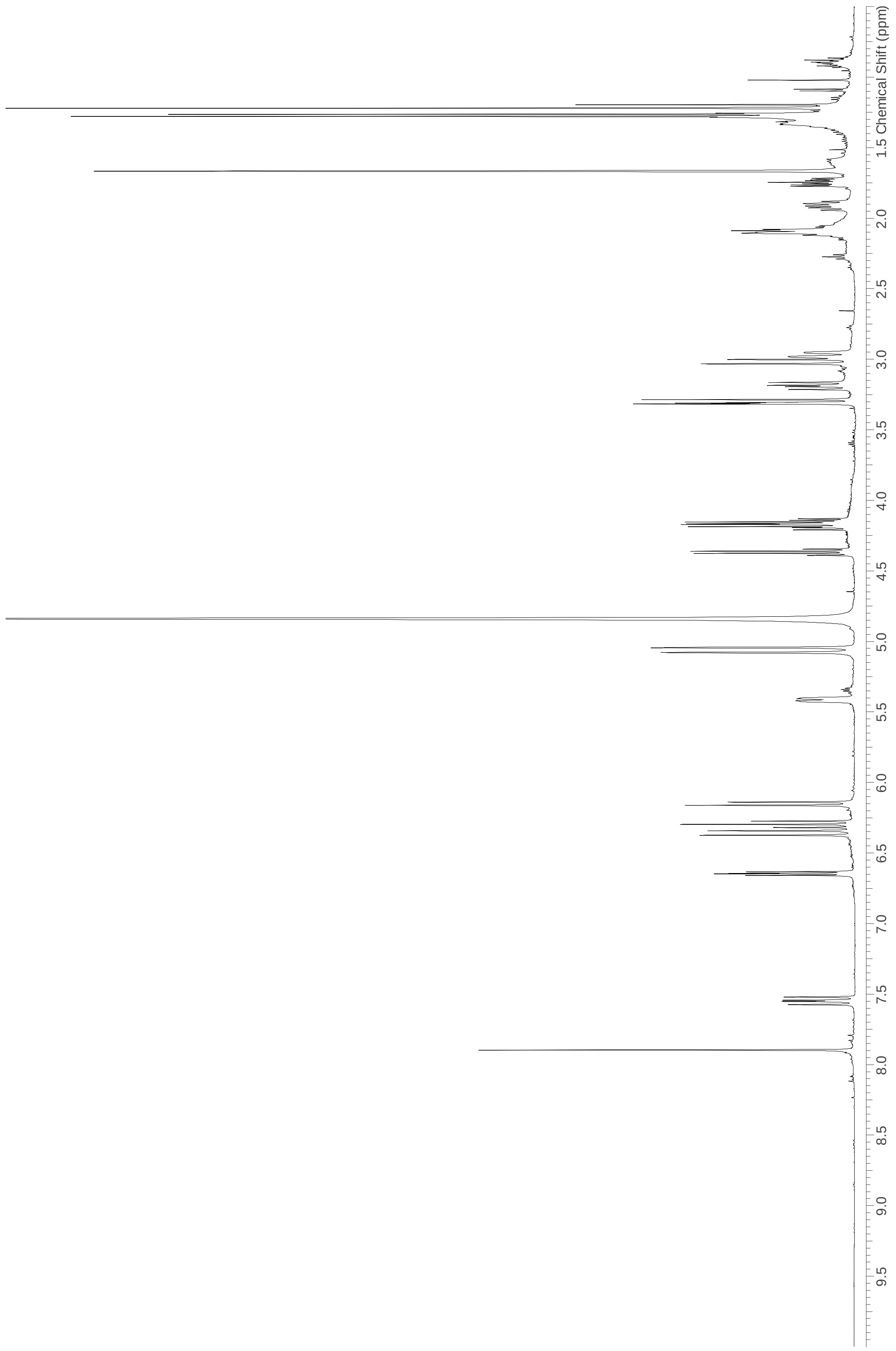

Figure S28. COSY spectrum of LRG C23 (**5**).

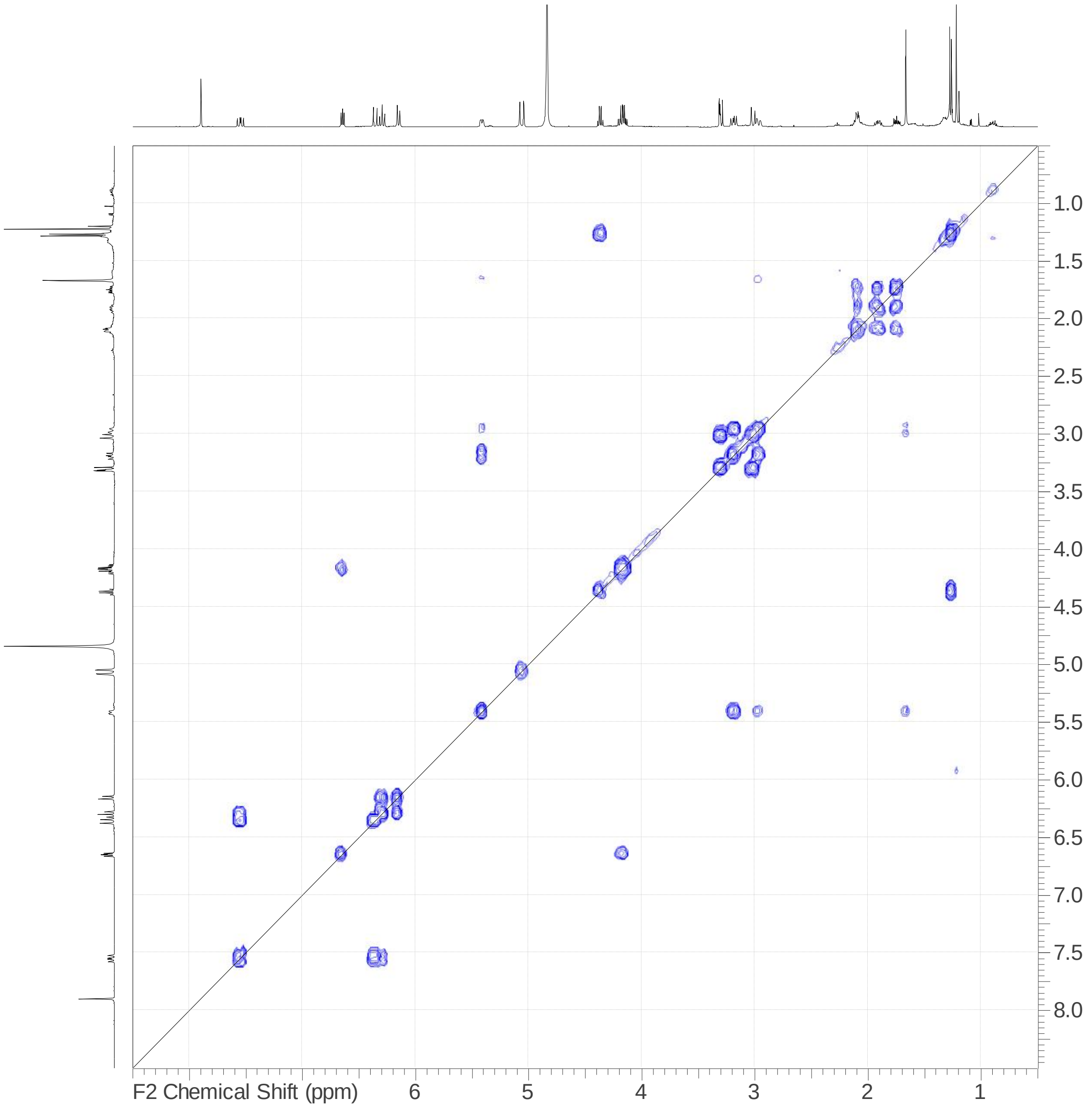

Figure S29. NOESY spectrum of LRG C23 (**5**).

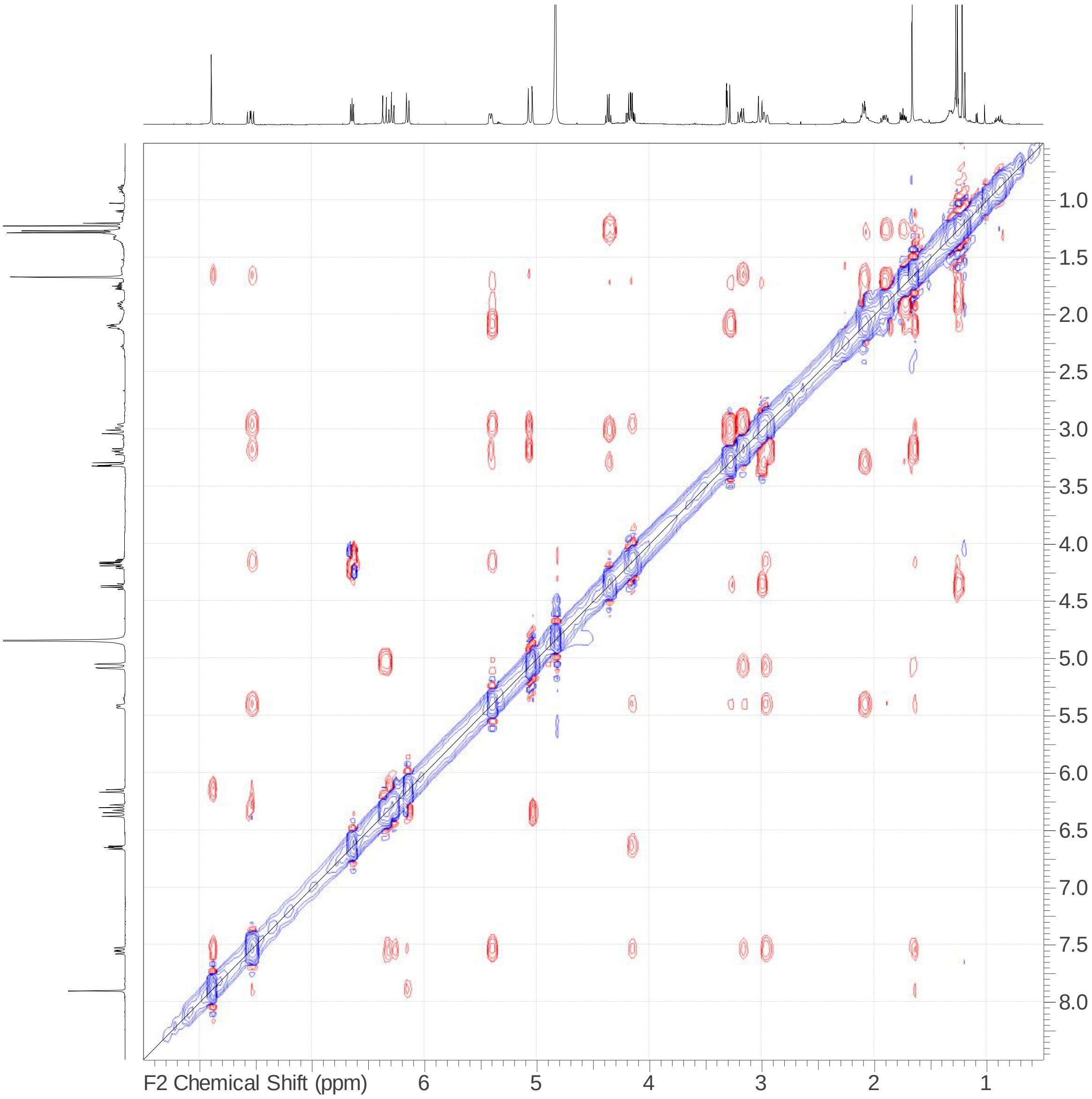

Figure S30. Edited HSQC spectrum of LRG C23 (**5**).

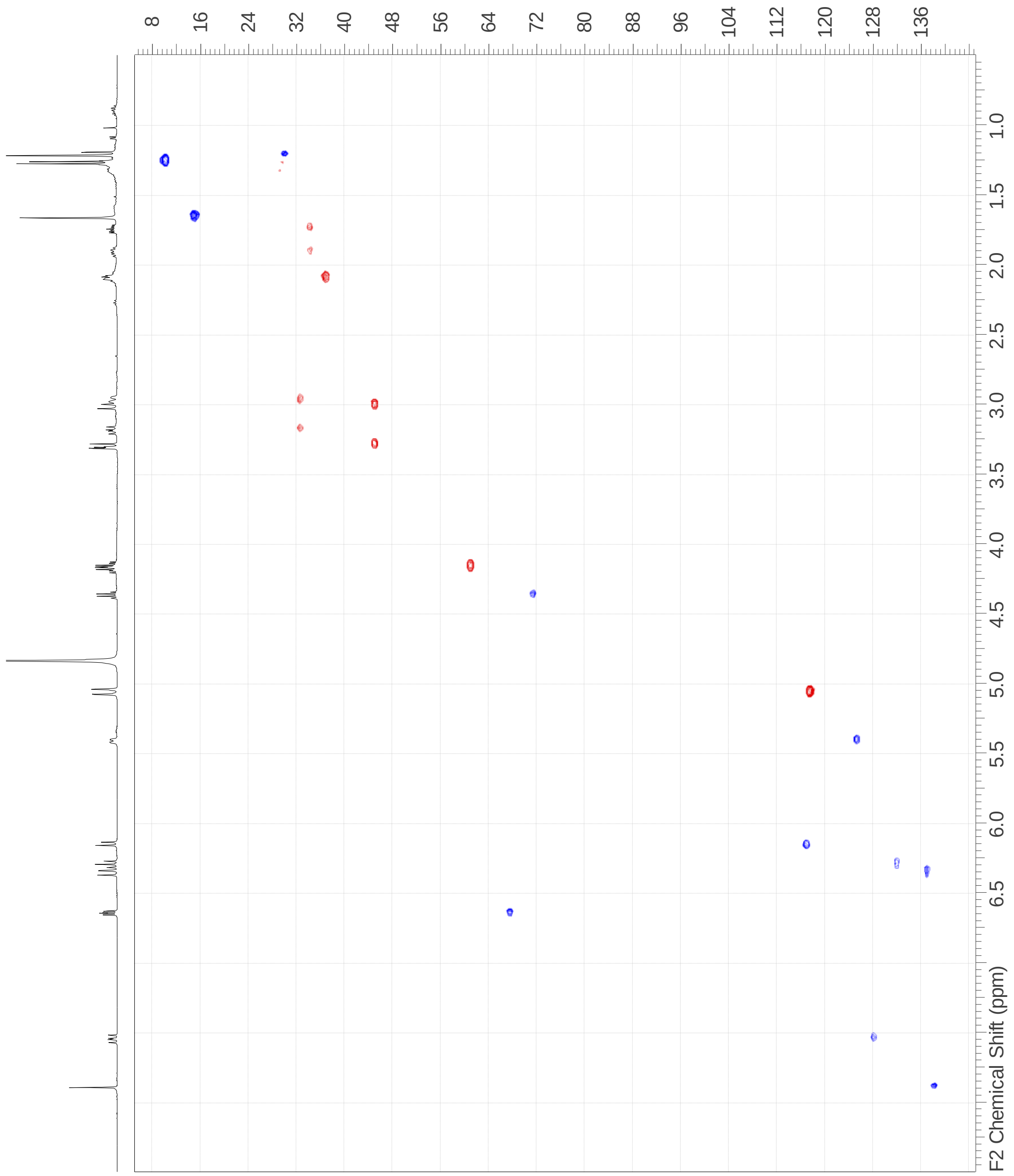

Figure S31. HMBC spectrum of LRG C23 (**5**).

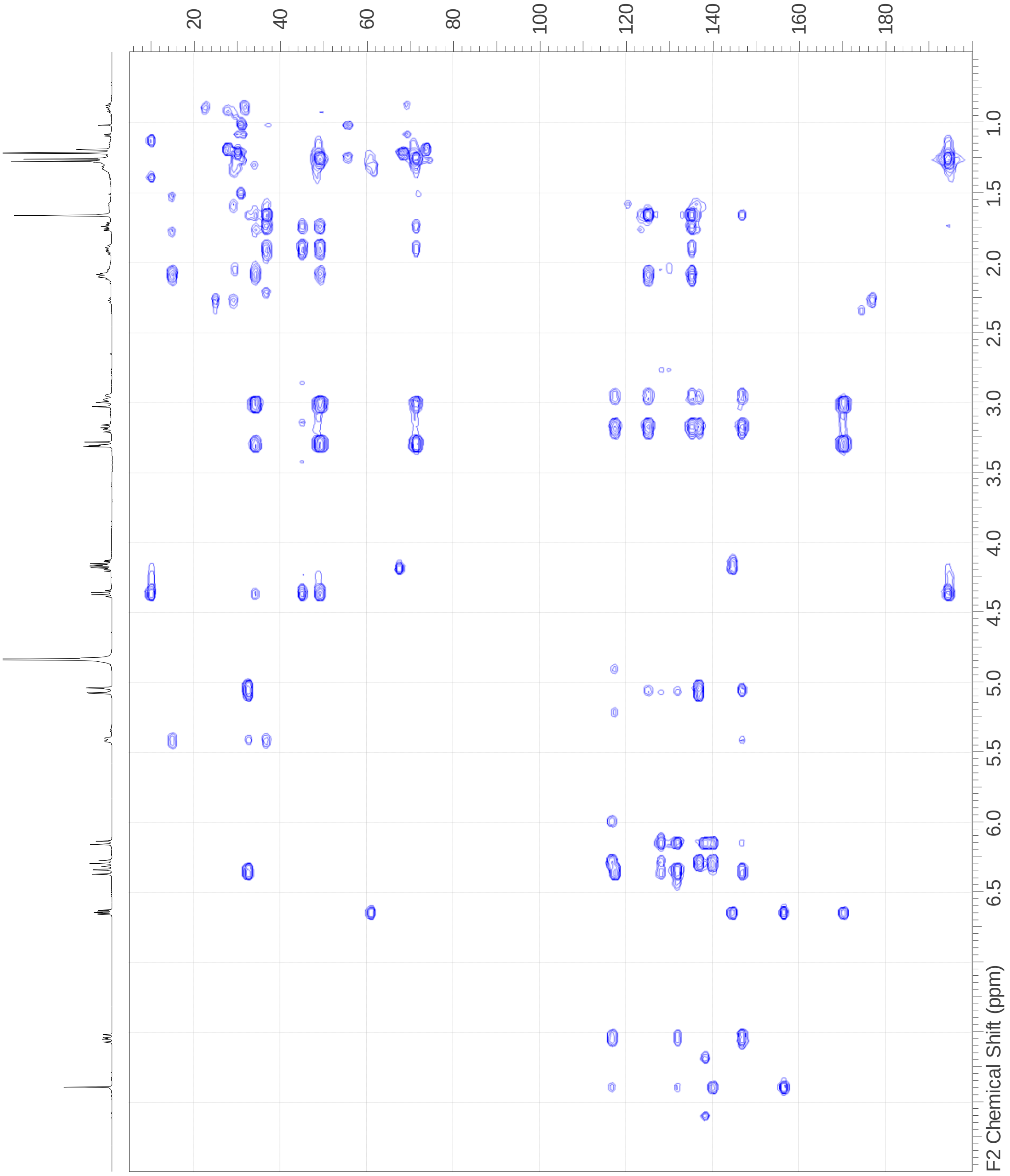

Figure S32. (A) UV-DAD spectrum of LRG C24 (**6**). (B) HRMS spectrum of LRG C24 (**6**).

A

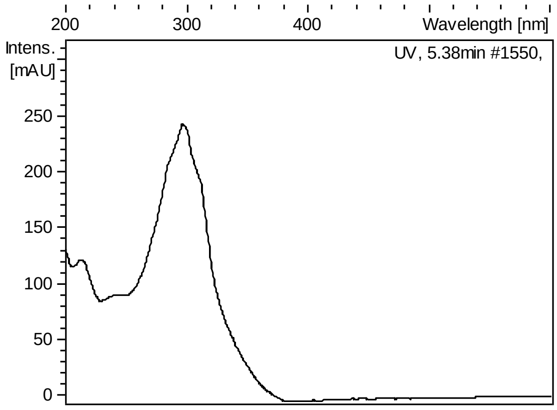

B

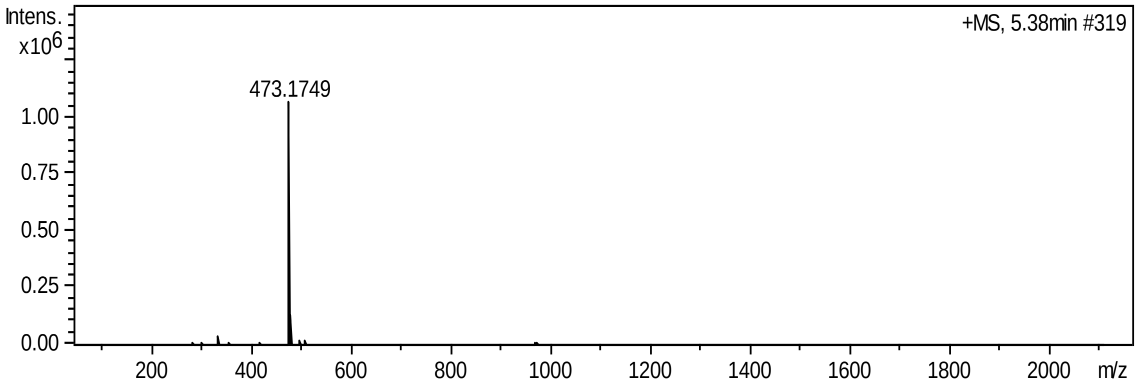

Figure S33. ^{1}H NMR spectrum of LRG C24 (**6**) (500 MHz, 24 ºC).

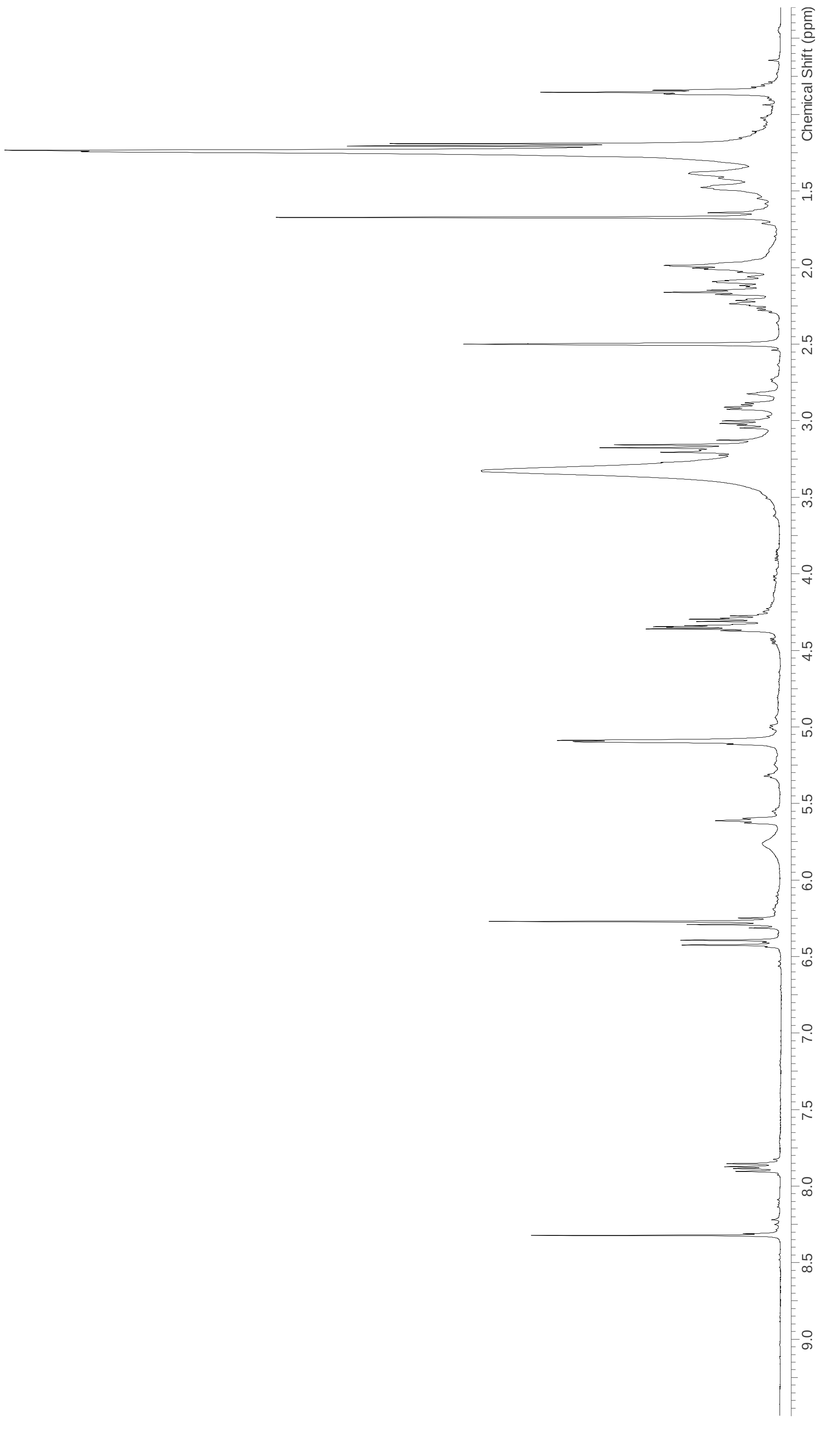

Figure S34. COSY spectrum of LRG C24 (**6**).

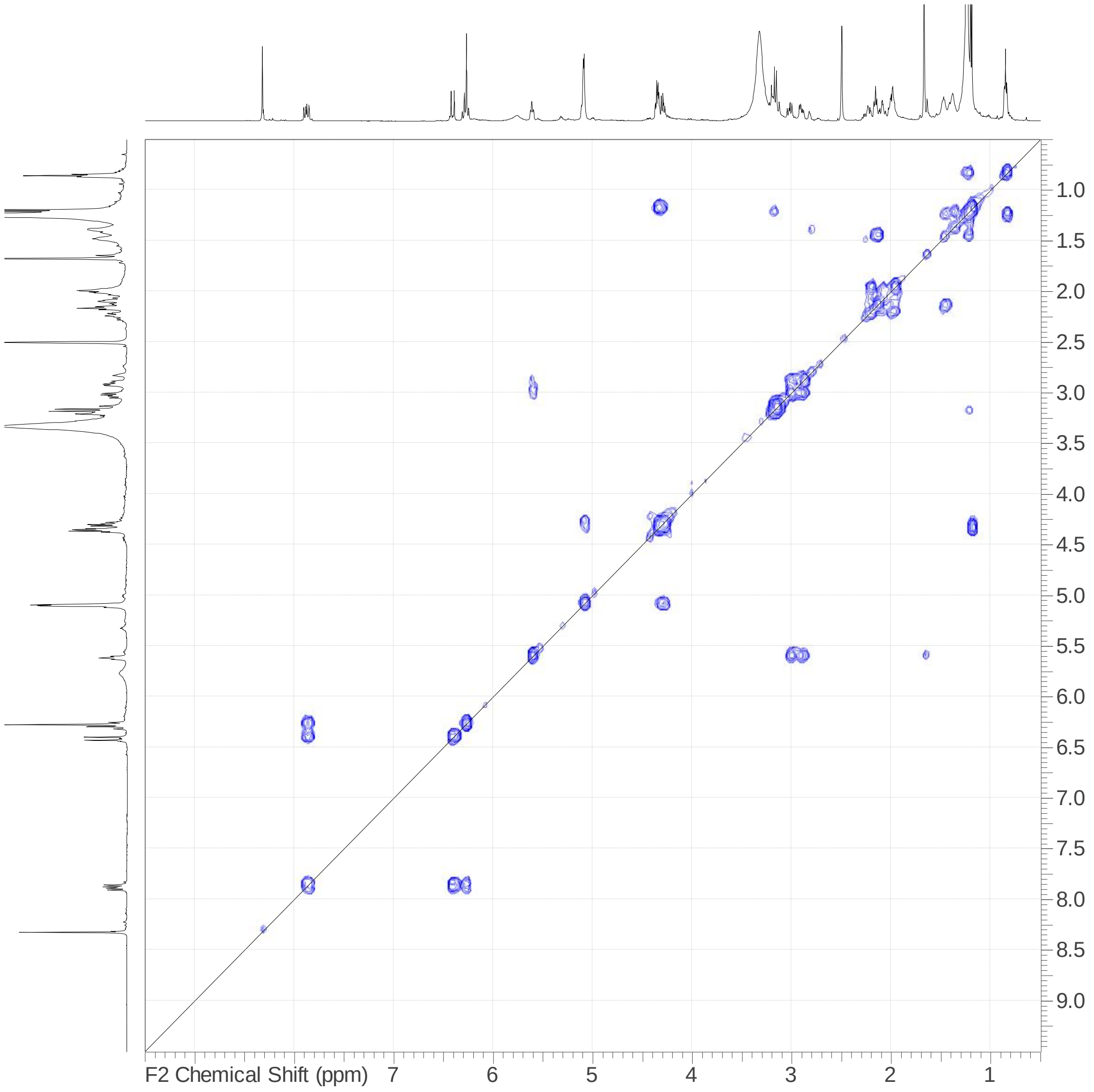

Figure S35. NOESY spectrum of LRG C24 (**6**).

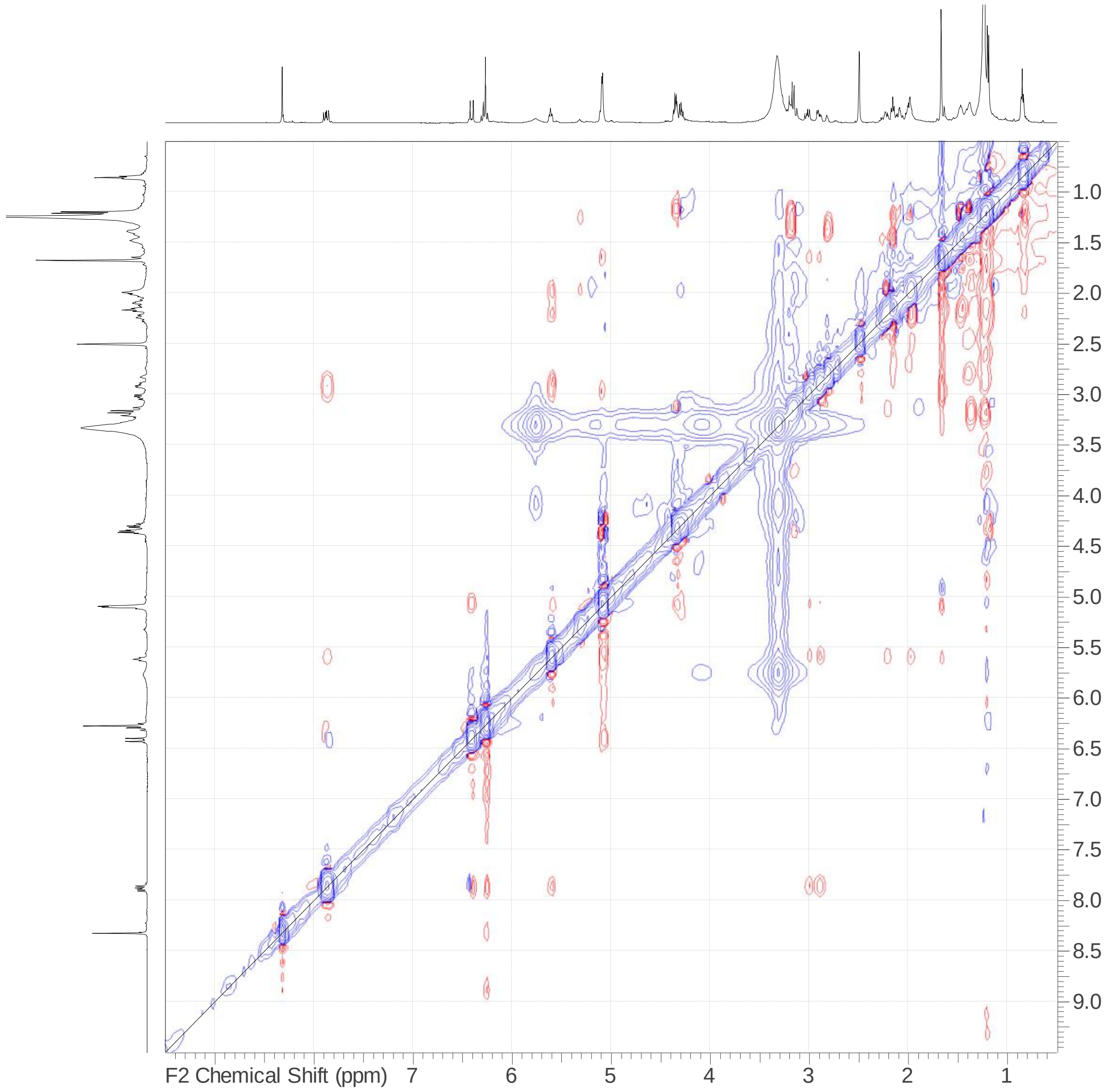

Figure S36. Edited HSQC spectrum of LRG C24 (**6**).

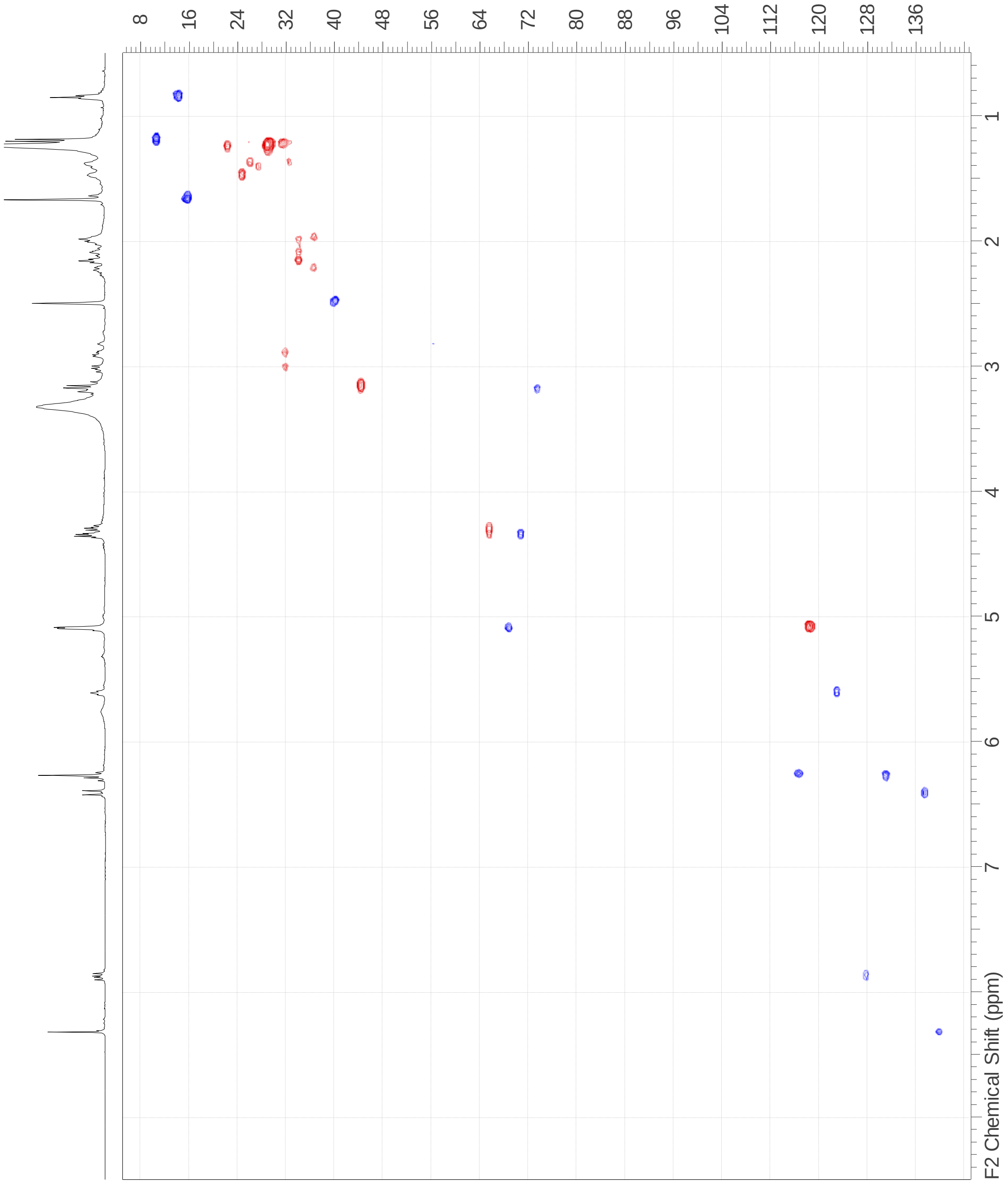

Figure S37. HMBC spectrum of LRG C24 (**6**).

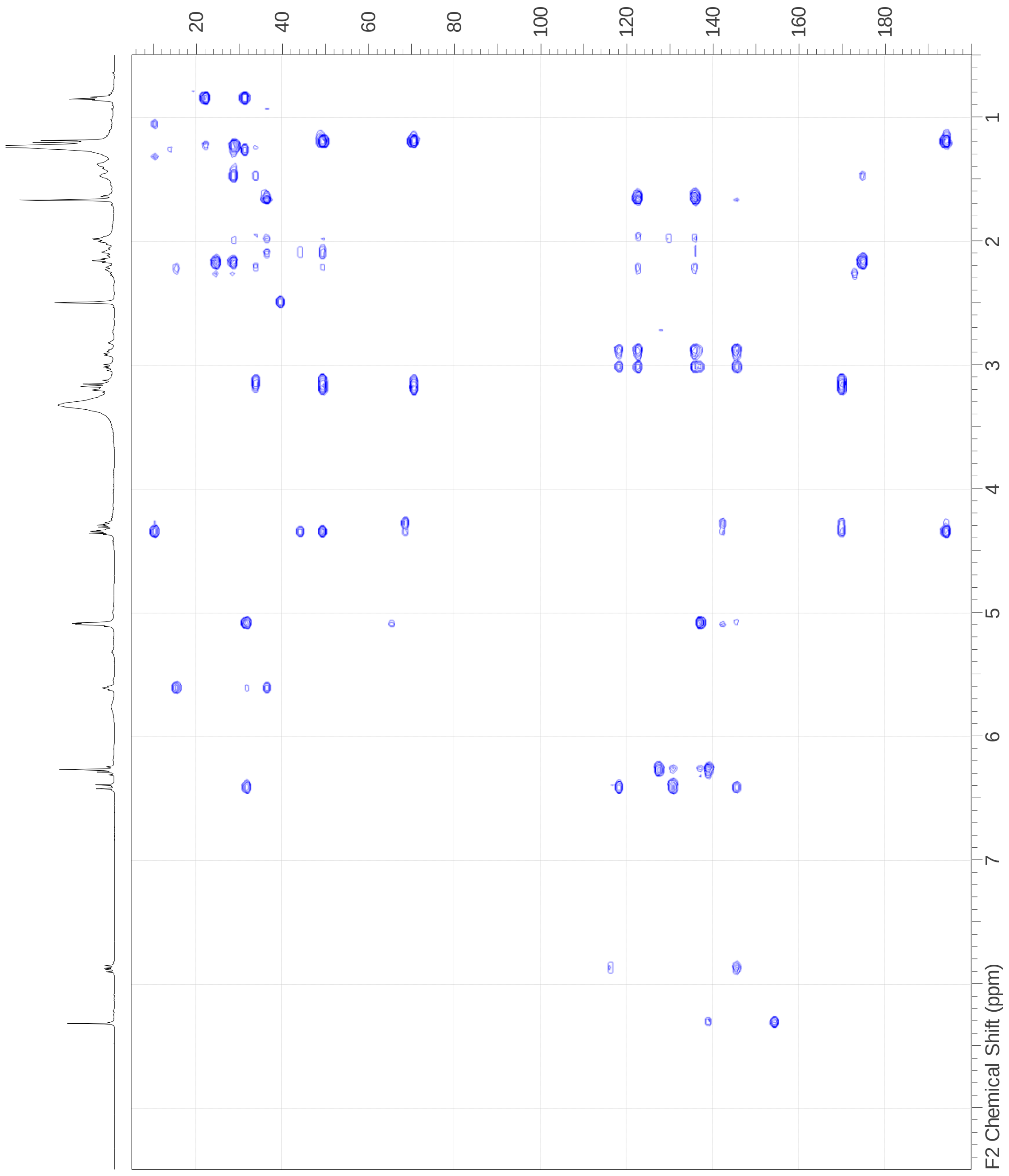

Figure S38. (A) UV-DAD spectrum of LRG C25 (**7**). (B) HRMS spectrum of LRG C25 (**7**).

A

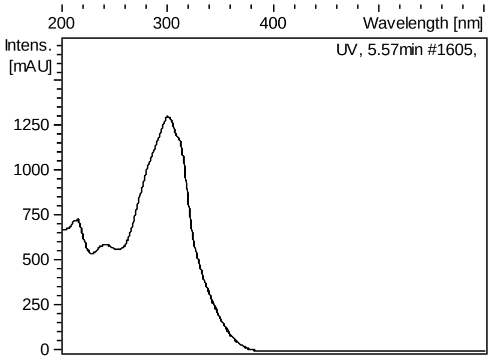

B

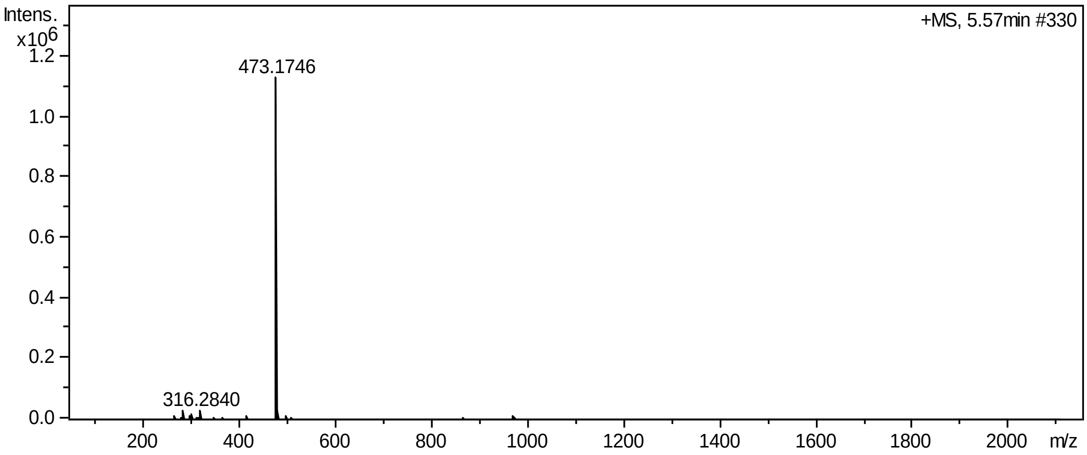

Figure S39. ^{1}H NMR spectrum of LRG C25 (**7**) (500 MHz, 24 ºC).

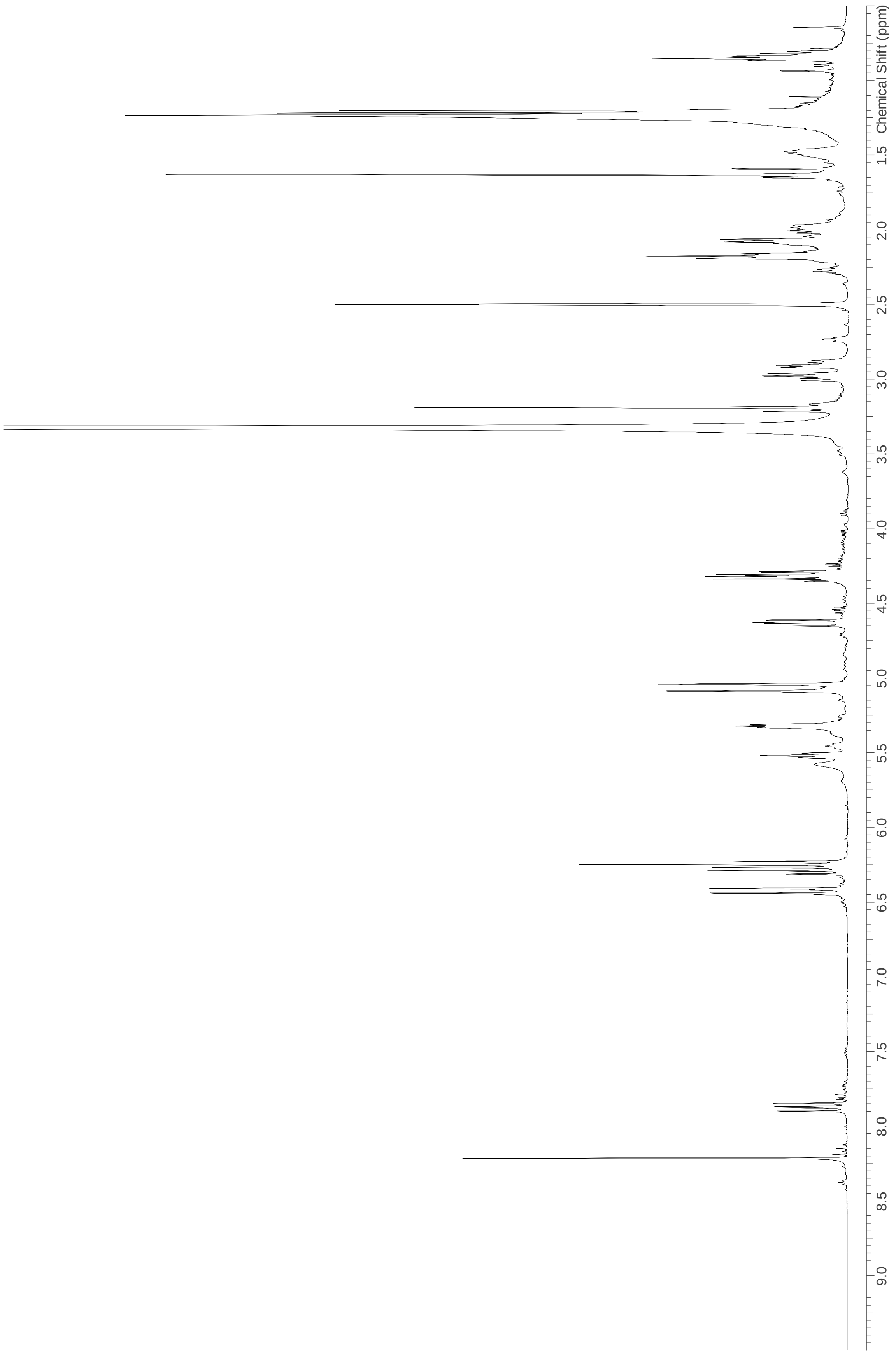

Figure S40. COSY spectrum of LRG C25 (**7**).

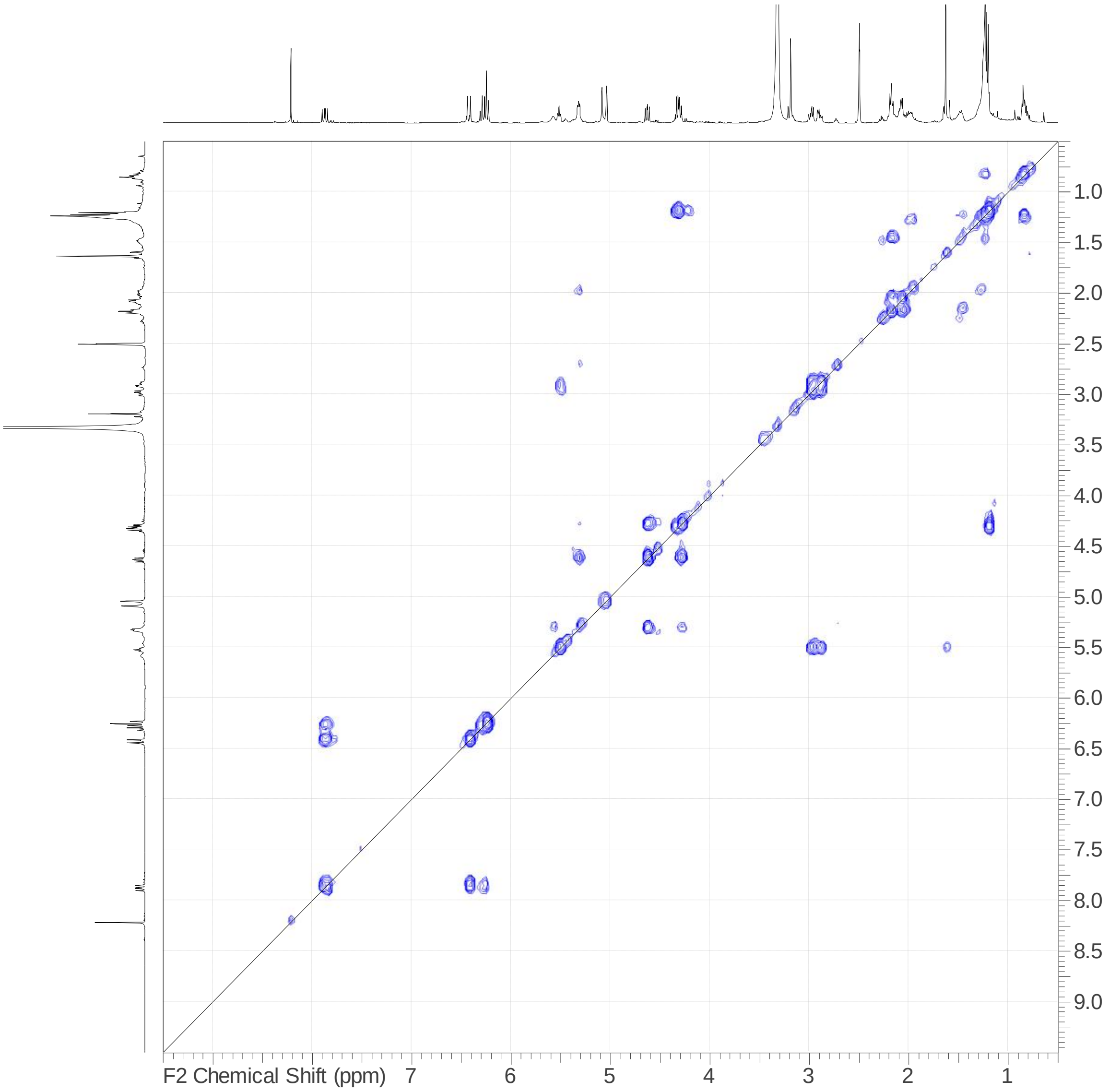

Figure S41. NOESY spectrum of LRG C25 (**7**).

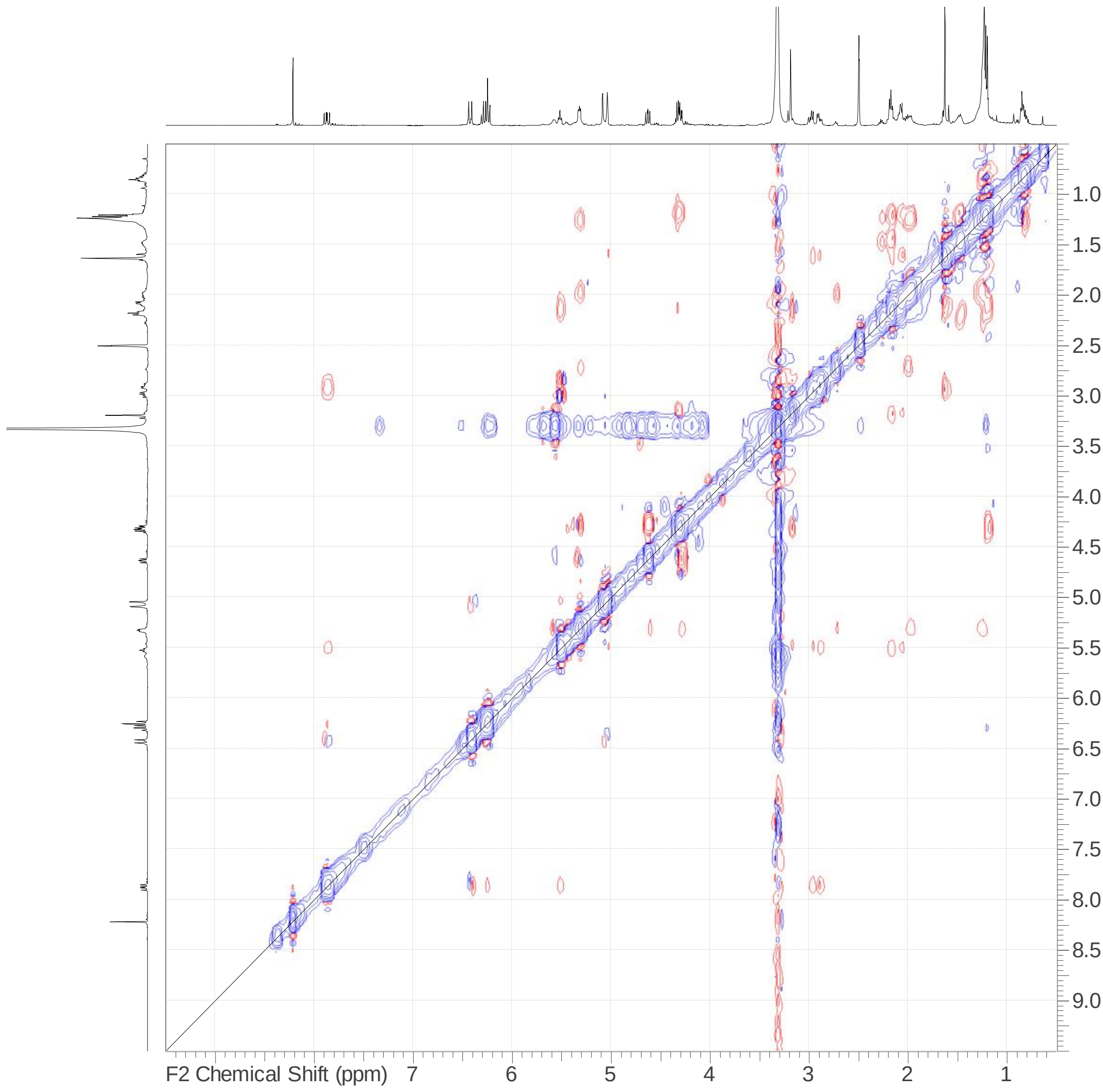

Figure S42. Edited HSQC spectrum of LRG C25 (**7**).

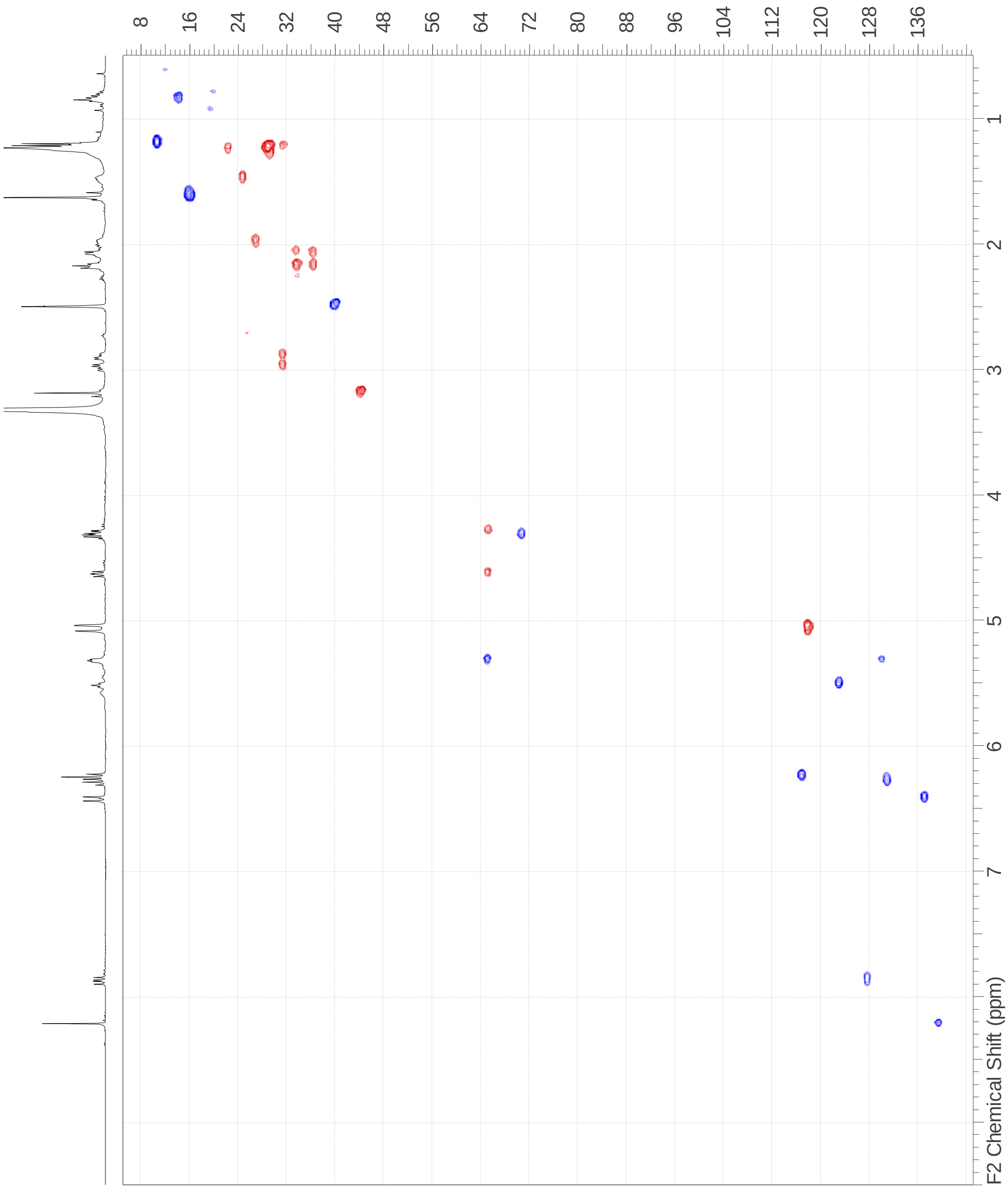

Figure S43. HMBC spectrum of LRG C25 (**7**).

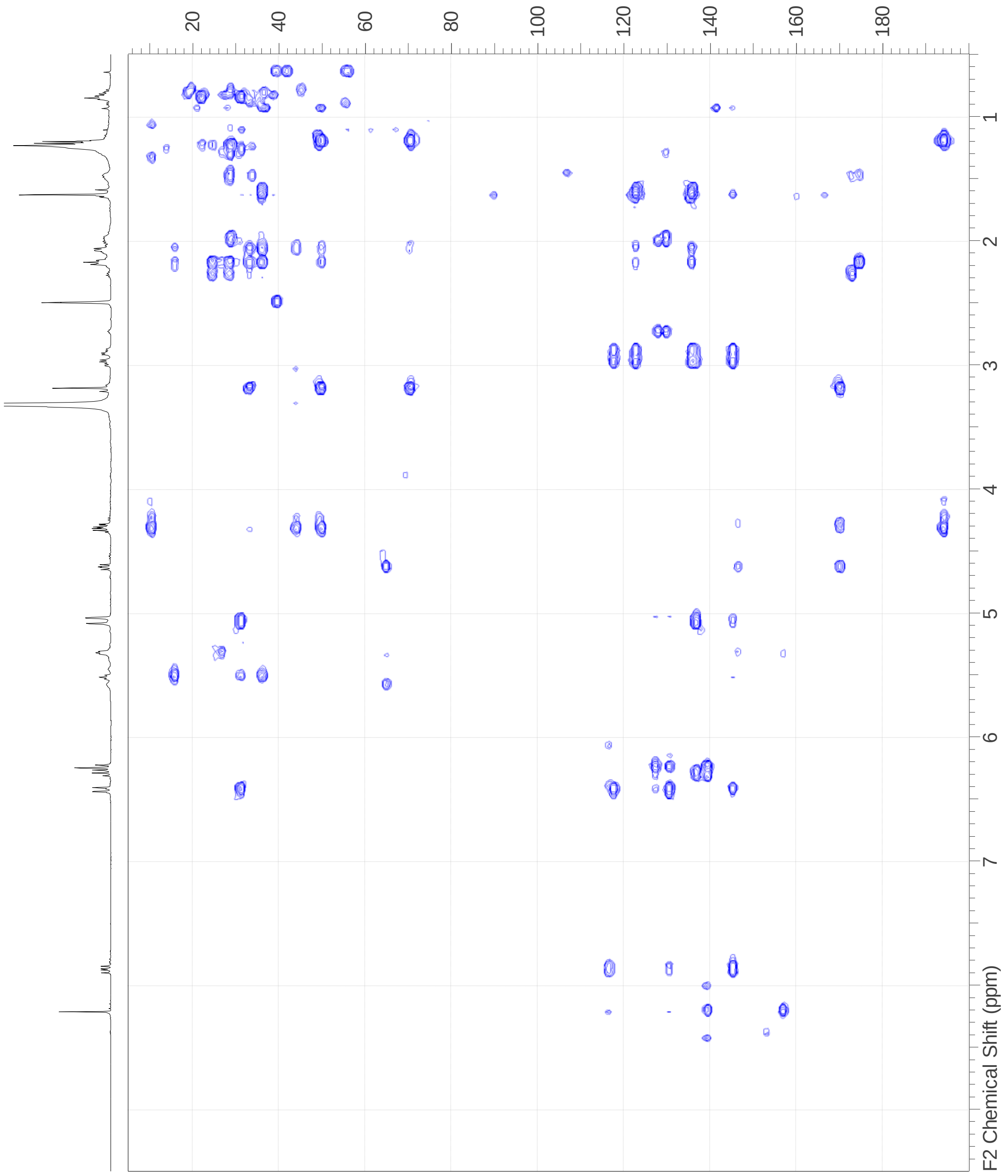

Figure S44. Key COSY correlations (bold bonds) and ^{1}H to ^{13}C HMBC correlations (blue arrows) determining the connectivity of LRG C11 (**1**), C12 (both conformers **2** and **3**), C22 (**4**), C23 (**5**), C24 (**6**) and C25 (**7**).

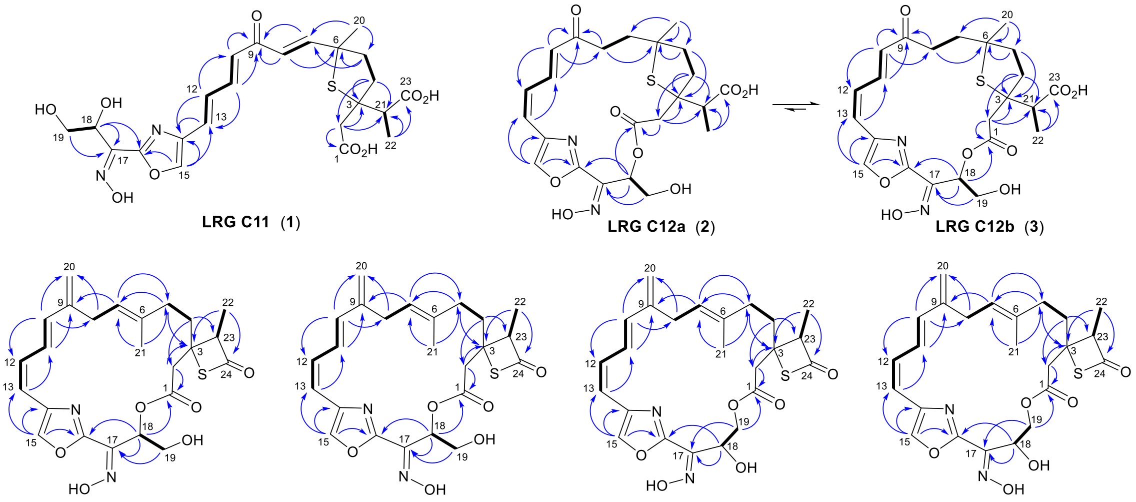

Figure S45. Equilibrium conformers LRG C12a and LRG C12b. Molecular mechanics calculations with the MM2 force field indicate that LRG C12b conformer is more stable than the other. The energy barrier for interconversion must be high to explain the slow interconversion observed by NMR.

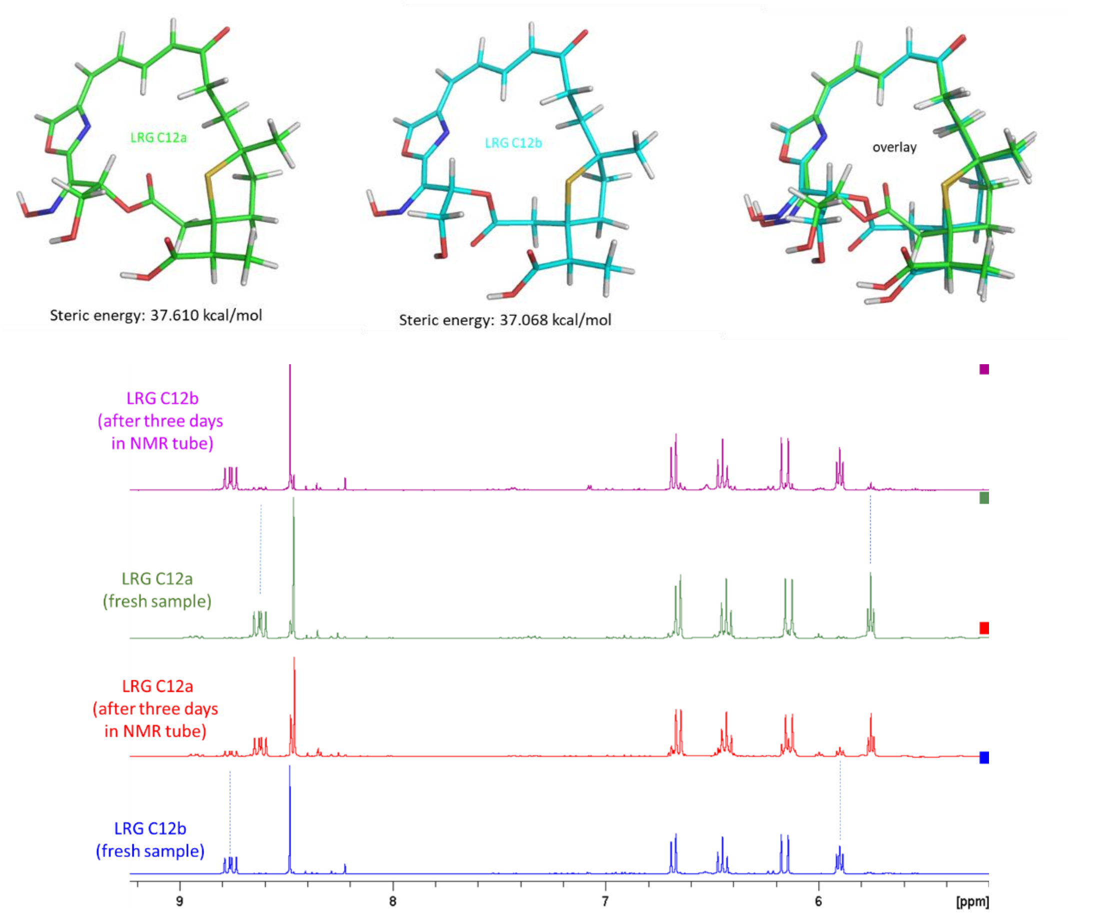

Table S1. Oligonucleotides used for PCR amplification

PRIMERS	SEQUENCE 5'-3'
Cit26I up	ATAGAATTCACTCCAGGCCCAGCGACT
Cit26I rp	AATAAGCTTGGCTGTGATGACCGGTC
Cit26D up	AAAGATATCTGGTCGAGCTGCCGGTC
Cit26D rp	TAATCTAGAGAGCCGGGGGTGGTGAT
ermECit26 up	GCAACTAGTTTCAGCCGGTCTAGGTTCG
ermECit26 rp	ATAGCTAGCATCCGGTGCGGGTCAGG
Cit26c up	GAGCCGACCTGCCGAGAA
Cit26c rp	TGCCAGGTGGTGCGGAA

Table S2. ^1H NMR (500 MHz) and ^{13}C NMR (125 MHz) data of LRG C11 (**1**) in DMSO-d_6.[a]

LRG C11		
**Position**	**δ_C, type**	**δ_H (*J* in Hz)**
1	172.5 C	
2	n. d., CH_2	a. 2.82, m<br>b. 2.76, m
3	62.8, C	
4	37.1, CH_2	a. 2.28, m<br>b. 2.17, m
5	42.3, CH_2	a. 2.18, m<br>b. 2.02, m
6	57.4, C	
7	152.4, CH	7.00, d (15.7)
8	124.2, CH	6.44, d (15.7)
9	189.1, C	
10	129.8, CH	6.77, d (15.3)
11	142.8, CH	7.46, ddd (15.3, 8.4, 2.0)
12	129.2, CH	7.11,dd (15.2, 8.5)
13	129.2, CH	7.10, overlap
14	138.7, C	
15	139.4, CH	8.42, s
16	154.8, C	
17	145.2, C	
18	72.5, CH	4.57, br dd (10.0, 5.3)
19	63.8, CH_2	a. 3.69, m<br>b. 3.63, m
20	28.0, CH_3	1.54, s
21	46.9, CH	2.94, m
22	14.8, CH_3	1.17, d (7.0)
23	176.1, C	

[a] ^{13}C chemical shifts determined from the indirect dimension of HSQC and HMBC spectra

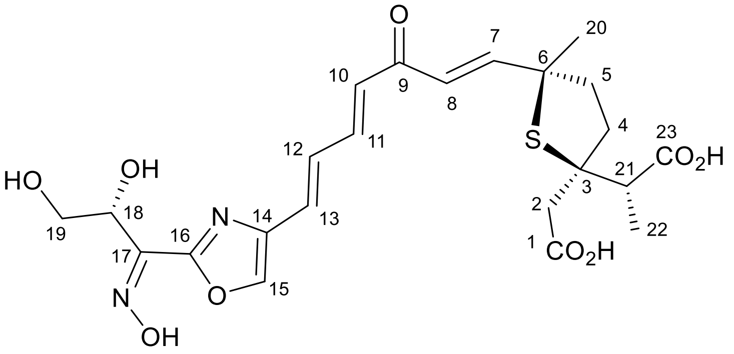

LRG C11

Table S3. ^{1}H NMR (500 MHz) and ^{13}C NMR (125 MHz) data of LRG C12a (**2**) in DMSO-d_6.[a]

LRG C12a		
**Position**	**δ_C, type**	**δ_H (*J* in Hz)**
1	170.5, C	
2	43.4, CH_2	a. 3.04, d (16.0)<br>b. 2.81, d (16.0)
3	60.7, C	
4	36.4, CH_2	a. 2.15, m<br>b. 2.00, m
5	40.1, CH_2	a. 1.89, m<br>b. 1.60, m
6	57.8, C	
7	35.2, CH_2	a. 1.98, m<br>b. 1.58, m
8	34.0, CH_2	a. 2.96, m<br>b. 2.75, m
9	201.2, C	
10	134.1, CH	6.13, d (16.0)
11	140.8, CH	8.61, dd (16.0, 11.3)
12	129.0, CH	6.43, t (11.2)
13	123.9, CH	6.65, d (11.1)
14	138.8, C	
15	141.5, CH	8.45, s
16	154.2, C	
17	142.2, C	
18	74.2, CH	5.75, t (6.7)
19	61.7, CH_2	3.64, d (6.7)
20	32.9, CH_3	1.41, s
21	46.8, CH	3.03, overlap
22	14.7, CH3	1.24, d (6.8)
23	176.3, C	

[a] ^{13}C chemical shifts determined from the indirect dimension of HSQC and HMBC spectra

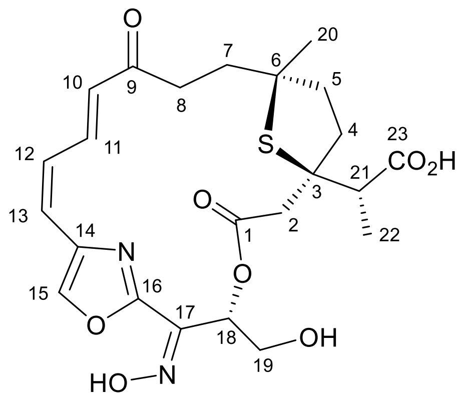

LRG C12a

Table S4. ^1H NMR (500 MHz) and ^{13}C NMR (125 MHz) data of LRG C12b (**3**) in DMSO-d_6.[a]

LRG C12b		
**Position**	**δ_C, type**	**δ_H (J in Hz)**
1	170.5, C	
2	43.5, CH_2	a. 2.88, d (14.2)<br>b. 2.70, d (14.2)
3	61.7, C	
4	36.3, CH_2	a. 2.17, ddd (12.9, 12.9, 5.6)<br>b. 2.02, br dd (13.2, 4.6)
5	39.3, CH_2	a. 1.92, ddd (12.9, 12.9, 5.7)<br>b. 1.51, br dd (13.2, 4.6)
6	57.7, C	
7	33.7, CH_2	a. 1.85, ddd (14.5, 5.4, 5.4)<br>b. 1.77, ddd (14.5, 10.2, 5.1)
8	33.4, CH_2	a. 3.02, ddd (13.2, 10.1, 5.2)<br>b. 2.90, overlap
9	201.0, C	
10	134.2, CH	6.15, d (16.1)
11	140.4, CH	8.75, dd (16.1, 11.3)
12	129.1, CH	6.44, t (11.3)
13	123.6, CH	6.67, d (11.3)
14	138.6, C	
15	141.4, CH	8.47, s
16	154.0, C	
17	140.4, C	
18	74.7, CH	5.89, dd (7.9, 6.8)
19	60.9, CH_2	a. 4.05, dd (10.0, 7.9)<br>b. 3.75, dd (10.0, 6.8)
20	33.2, CH_3	1.40, s
21	47.6, CH	3.16, q (7.2)
22	14.7, CH3	1.27, d (7.2)
23	176.2, C	

[a] ^{13}C chemical shifts determined from the indirect dimension of HSQC and HMBC spectra

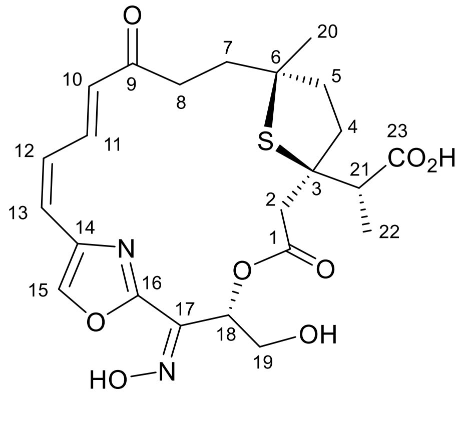

LRG C12b

Table S5. ^{1}H NMR (500 MHz) and ^{13}C NMR (125 MHz) data of LRG C22 (**4**) in CD_3OD.[a]

	LRG C22	
**Position**	**δ_C, type**	**δ_H (J in Hz)**
1	169.9, C	
2	45.8, CH_2	a. 3.23, d (15.4)<br>b. 3.09, d (15.4)
3	49.0, C	
4	34.9, CH_2	a. 1.89, ddd (12.9, 9.3, 7.5)<br>b. 1.77, ddd (12.5, 12.5, 5.2)
5	36.6, CH_2	2.06, br t (7.8)
6	135.0, C	
7	125.1, CH	5.36, dd (9.8, 2.5)
8	33.2, CH_2	a. 3.20, dd (14.9, 9.7)<br>b. 2.97, dd (14.9, 2.4)
9	147.0, C	
10	137.6, CH	6.37, d (15.7)
11	128.0, CH	7.53, dd (15.7, 11.3)
12	132.3, CH	6.30, t (11.3)
13	116.7, CH	6.18, d (11.3)
14	140.0, C	
15	138.0, CH	7.99, s
16	153.2, C	
17	140.8, C	
18	75.4, CH	5.95, dd (7.7, 5.8)
19	61.3, CH_2	a. 4.19, dd (11.6, 5.8)<br>b. 4.07, dd (11.6, 7.7)
20	117.9, CH_2	a. 5.09, br s<br>b. 5.05, br s
21	15.7, CH_3	1.73, s
22	71.8, CH	4.29, q (7.4)
23	10.4, CH_3	1.14, d (7.4)
24	194.5, C	

[a] ^{13}C chemical shifts determined from the indirect dimension of HSQC and HMBC spectra

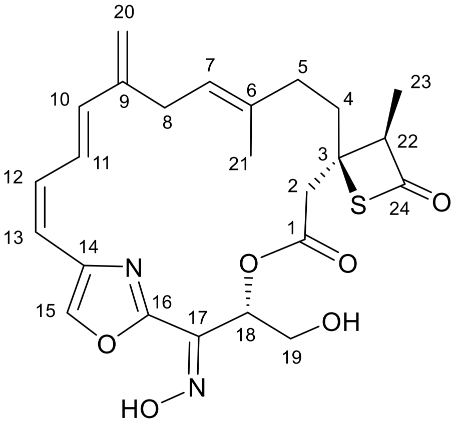

LRG C22

Table S6. ^{1}H NMR (500 MHz) and ^{13}C NMR (125 MHz) data of LRG C23 (**5**) in CD_3OD.[a]

	**LRG C23**	
**Position**	**δ_C, type**	**δ_H (*J* in Hz)**
1	170.4, C	
2	45.3, CH_2	a. 3.30, d (15.4)<br>b. 3.02, d (15.4)
3	49.6, C	
4	34.6, CH_2	a. 1.91, ddd (14.0, 9.3, 7.5)<br>b. 1.75, ddd (14.0, 8.9, 5.3)
5	37.0, CH_2	2.09, m
6	135.4, C	
7	125.4, CH	5.41, dd (9.8, 2.5)
8	32.9, CH_2	a. 3.19, dd (14.5, 9.9)<br>b. 2.97, dd (14.5, 2.4)
9	147.1, C	
10	137.1, CH	6.36, d (15.6)
11	128.2, CH	7.55, dd (15.6, 11.3)
12	132.1, CH	6.30, t (11.3)
13	117.0, CH	6.16, d (11.3)
14	140.3, C	
15	138.3, CH	7.90, s
16	156.6, C	
17	144.8, C	
18	67.8, CH	6.64, dd (7.6, 5.7)
19	61.3, CH_2	a. 4.19, dd (11.3, 5.7)<br>b. 4.15, dd (11.3, 7.6)
20	117.7, CH_2	a. 5.08, br s<br>b. 5.04, br s
21	15.3, CH_3	1.67, s
22	71.7, CH	4.37, q (7.5)
23	10.4, CH_3	1.27, d (7.5)
24	194.7, C	

[a] ^{13}C chemical shifts determined from the indirect dimension of HSQC and HMBC spectra

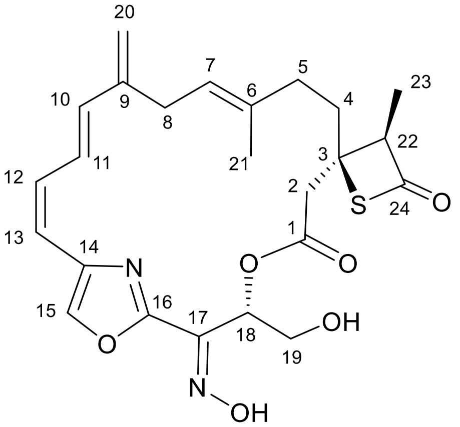

LRG C23

Table S7. ^{1}H NMR (500 MHz) and ^{13}C NMR (125 MHz) data of LRG C24 (**6**) in DMSO-d_6.[a]

	**LRG C24**	
**Position**	**δ_C, type**	**δ_H (*J* in Hz)**
1	170.2, C	
2	44.6, CH_2	a. 3.20, d (15.3)<br>b. 3.14, d (15.3)
3	49.9, C	
4	34.4, CH_2	a. 2.10, m<br>b. 2.00, overlap
5	36.8, CH_2	a. 2.23, m<br>b. 1.99, overlap
6	136.2, C	
7	123.0, CH	5.61, br t (7.7)
8	32.1, CH_2	a. 3.02, dd (14.4, 8.7)<br>b. 2.90, dd (14.3, 6.4)
9	145.9, C	
10	137.5, CH	6.41, d (15.7)
11	127.9, CH	7.88, dd (15.7, 9.5)
12	131.3, CH	6.29, t (11.3)
13	116.8, CH	6.26, d (11.3)
14	139.3, C	
15	139.9, CH	8.32, s
16	154.6, C	
17	142.7, C	
18	69.0, CH	5.10, overlap
19	65.8, CH_2	a. 4.35, overlap<br>b. 4.29, dd (10.7, 7.2)
20	118.6, CH_2	a. 5.10, br s<br>b. 5.09, br s
21	15.9, CH_3	1.67, s
22	71.0, CH	4.35, overlap
23	10.8, CH_3	1.20, d (7.6)
24	194.4, C	

[a] ^{13}C chemical shifts determined from the indirect dimension of HSQC and HMBC spectra

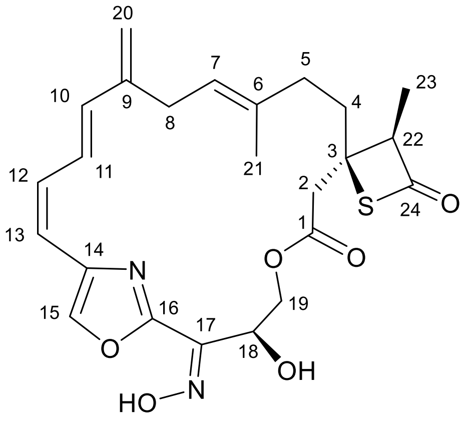

LRG C24

Table S8. ^1H NMR (500 MHz) and ^{13}C NMR (125 MHz) data of LRG C25 (**7**) in DMSO-d_6.[a]

	**LRG C25**	
**Position**	**δ_C, type**	**δ_H (*J* in Hz)**
1	170.4, C	
2	44.4, CH_2	a. 3.19, br t (15.0)
3	50.2, C	
4	33.8, CH_2	a. 2.18, overlap<br>b. 2.08, overlap
5	36.6, CH_2	a. 2.19, overlap<br>b. 2.09, overlap
6	136.1, C	
7	123.1, CH	5.52, br t (7.2)
8	31.6, CH_2	a. 2.99, dd (15.1, 8.1)<br>b. 2.90, dd (15.1, 6.8)
9	145.7, C	
10	137.2, CH	6.42, d (15.7)
11	127.8, CH	7.87, dd (15.7, 10.7)
12	131.0, CH	6.29, t (11.3)
13	117.0, CH	6.24, d (11.3)
14	139.7, C	
15	139.4, CH	8.22, s
16	157.3, C	
17	146.8, C	
18	65.3, CH	5.32, br t (5.2)
19	65.4, CH_2	a. 4.63, dd (11.7, 8.8)<br>b. 4.30, overlap
20	118.0, CH_2	a. 5.09, br s<br>b. 5.04, br s
21	15.9, CH_3	1.63, s
22	71.0, CH	4.33, overlap
23	10.8, CH_3	1.21, d (7.6)
24	194.4, C	

[a] ^{13}C chemical shifts determined from the indirect dimension of HSQC and HMBC spectra

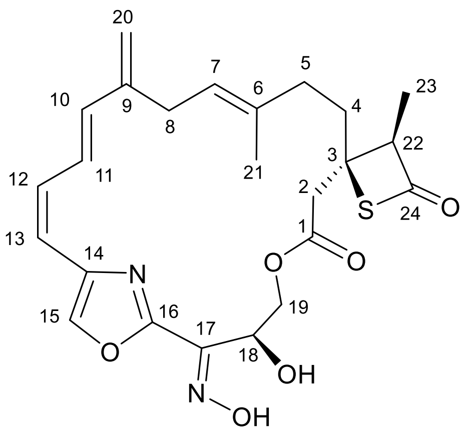

LRG C25

References

(1) Becerril, A.; Pérez-Victoria, I.; Ye, S.; Braña, A. F.; Martín, J.; Reyes, F.; Salas, J. A.; Méndez, C. *ACS Chem Biol* **2020**, 15 (6), 1541-1553.
(2) Becerril, A.; Pérez-Victoria, I.; Martín, J. M.; Reyes, F.; Salas, J. A.; Méndez, C. *ACS Chem Biol* **2022**, 17 (8), 2320-2331.
(3) Huang, S. X.; Yun, B. S.; Ma, M.; Basu, H. S.; Church, D. R.; Ingenhorst, G.; Huang, Y.; Yang, D.; Lohman, J. R.; Tang, G. L.; Ju, J.; Liu, T.; Wilding, G.; Shen, B. *Proc Natl Acad Sci USA* **2015**, 112 (27), 8278-8283.
(4) Lee, H. B.; Park, H. Y.; Lee, B. S.; Kim, Y. G. *Magn Reson Chem* **2000**, 38 (6), 468-471.
(5) Hawkes, G. E.; Herwig, K.; Roberts, J. D. *J Org Chem* **1974**, 39 (8), 1017-1028.
